# What do women want? An analysis of preferences of women, involvement of men, and decision-making in maternal and newborn health care in rural Bangladesh

**DOI:** 10.1186/s12884-020-2854-x

**Published:** 2020-03-18

**Authors:** Ahmed Ehsanur Rahman, Janet Perkins, Shumona Sharmin Salam, Shema Mhajabin, Aniqa Tasnim Hossain, Tapas Mazumder, Shams EL Arifeen

**Affiliations:** 1 Maternal and Child Health Division, icddr,b, Dhaka, Bangladesh; 2 University of Edinburgh, Edinburgh, UK

**Keywords:** Maternal health, Newborn health, Pregnant women, Respectful maternity care, Rights, Male involvement

## Abstract

**Background:**

To improve the utilization of maternal and newborn health (MNH) care and to improve the quality of care, the World Health Organization (WHO) has strongly recommended men’s involvement in pregnancy, childbirth, and after birth. In this article, we examine women’s preferences for men’s involvement in MNH care in rural Bangladesh and how this compares to husbands’ reported involvement by women.

**Methods:**

A cross-sectional household survey of 1367 women was administered in 2018 in the district of Brahmanbaria. Outcomes of interest included supporting self-care during pregnancy, participation in birth planning, presence during antenatal care, childbirth, and postnatal care, and participation in newborn care. Binary and multiple logistic regressions were done to understand the associations between the outcomes of interest and background characteristics.

**Results:**

Although women preferred a high level of involvement of their husbands in MNH care, husbands’ reported involvement varied across different categories of involvement. However, women’s preferences were closely associated with husbands’ reported involvement. Around three-quarters of the women reported having been the primary decision makers or reported that they made the decisions jointly with their husbands. The likelihood of women reporting their husbands were actively involved in MNH care was 2.89 times higher when the women preferred their husbands to be involved in 3–4 aspects of MNH care. The likelihood increased to 3.65 times when the women preferred their husbands to be involved in 5–6 aspects. Similarly, the likelihood of husbands’ reported active involvement was 1.43 times higher when they jointly participated in 1–2 categories of decision-making. The likelihood increased to 2.02 times when they jointly participated in all three categories.

**Conclusion:**

The findings of our study suggest that women in rural Bangladesh do indeed desire to have their husbands involved in their care during pregnancy, birth and following birth. Moreover, their preferences were closely associated with husbands’ reported involvement in MNH care; that is to say, when women wanted their husbands to be involved, they were more likely to do so. Programmes and initiatives should acknowledge this, recognizing the many ways in which men are already involved and further allow women’s preferences to be realized by creating an enabling environment at home and in health facilities for husbands to participate in MNH care.

## Background

The International Conference on Population and Development (ICPD), held in Cairo in 1994, represented a critical turning point in the global discourses around sexual and reproductive health [1, 2]. Among the paradigm shifts consolidated during the conference and in its programme of action was a nascent emphasis on the importance of engaging men along with women in reproductive, maternal, newborn and child health (RMNCH) [3]. In response, an increasing number of initiatives began to target and involve men in RMNCH programming globally [4–8]. In 2015, the World Health Organization (WHO) reviewed the existing evidence and issued a strong recommendation in favour of promoting men’s involvement for improving the care of women during pregnancy, childbirth and following birth, and for increasing the utilisation of skilled health services. As a result, the role of men has gained a renewed focus and prominence in maternal and newborn health (MNH)-specific policies and programming globally [5, 9].

Women residing in rural Bangladesh, as well as South Asia more generally, are often portrayed as living under a system of classic patriarchy, much of which plays out within the household [10–12]. Within this representation, women’s preferences within the household hold minimal importance, with men serving as primary decision-makers and household gatekeepers, in many ways determining women’s movement, conditions and access to resources, including during the perinatal period [13–18]. This has been suggested as a potential contributor to poor care of pregnant women, with pregnancy and childbirth being considered primarily as women’s issues, further inhibiting women in discussing their reproductive health needs and availing MNH services [18–23]. Low use of key MNH services is often cited as a manifestation of this situation, as only 37% of pregnant women attend at least four antenatal care (ANC) contacts and just half of births occur in health facilities [24].

Enthusiasm in favour of social projects aiming to influence gender dynamics concerning MNH care is high in Bangladesh as a potential strategy for improving the care of pregnant women and newborns, which is reflected in national policies and programming [25–27]. Indeed, some studies have suggested that effectively engaging men may be a promising strategy for increasing use of skilled services in the context of rural Bangladesh [9, 20]. However, caution is required, as some studies from South Asia have found that promoting men’s involvement in maternal health can lead to unintended negative consequences, potentially compromising women’s decision-making and reinforcing unbalanced gender power dynamics. Moreover, programmes may risk undermining the preferences and agency of women by promoting involvement of men which is not desired [15, 28].

It is therefore critical to gain a greater understanding of women’s preferences, as well as the reported involvement of men in MNH in rural Bangladesh in order to avoid negative consequences in the midst of enthusiasm pushing interventions promoting the involvement of men in MNH forward. In this article, we examine the preferences of women from rural Bangladesh related to the involvement of their husbands in MNH care and how this plays out within households. From here on, we privilege the term “husband” rather than “men” in order to reflect the local context. In rural Bangladesh male partners continue to be exclusively husbands and using the term male partner rather than husband has a socially negative connotation. We begin by presenting how women would like their husbands to be involved in their care during pregnancy, birth and following birth. We then explore how these preferences compare to the husbands’ reported involvement in care. Finally, we look at how decision-making related to MNH is occurring and how this influences the involvement of men.

## Methods

### Study design and settings

A cross-sectional survey was conducted in the households of three sub-districts of Brahmanbaria district in Bangladesh in 2018. Brahmanbaria district is located in the central-east region, which is 102 km away from Dhaka, the capital of Bangladesh. The three sub-districts which were selected in this study were Bijoynagar, Kasba and Sarail; each with an approximate population of 300,000 [29]. Population, administrative structure and health systems details of the three sub-districts are summarised in additional table 1.

### Study population, sample size and sampling

Women who had given birth in the 12 months preceding the survey and who were permanent residents of the study sub-districts were considered to be eligible and were identified through stratified cluster sampling. The three sub-districts were considered as the strata, and the villages, with an approximate population of 1000, were regarded as the clusters [30, 31]. Probability proportional to size (PPS) sampling was adopted to select 20 villages/clusters from each sub-district (strata) [31–33]. A sketch map was drawn for the selected villages indicating village boundaries, household locations, and important landmarks. Then all households were enumerated and listed, following which all women who had a birth in the past 12 months were identified within each household using a screening form. All eligible women from the selected villages/clusters were invited to participate in the survey. A total of 1367 women were successfully interviewed using an interviewer-administered structured questionnaire. The non-response rate was less than 1%. This was achieved by making up to three consecutive visits to households at different times and on different days of the week when women were not available at the first and second attempts. In addition, the data collectors were locally recruited, which facilitated their access to the communities and their ability to build rapport with the respondents.

### Data collection

Data were collected between March and May of 2018. All eligible women were approached for an interview by trained interviewers (20 females and 13 male) who administered the structured questionnaire. Women were interviewed in their households alone, without the presence of their husbands or other family members.

The questionnaire included a number of questions regarding knowledge, preference and practices of women, their husbands and families around MNH including husband involvement. The data collection tools were pre-tested in the non-selected clusters/villages of the sub-districts, and then the tools were revised based on the pre-test findings.

Data collectors were recruited locally so that they would be familiar with the local language, culture, and norms. They received 3 days of extensive training by master trainers of icddr,b (formerly known as the International Centre of Diarrhoeal Disease Research, Bangladesh), followed by 4 days of field practice. Furthermore, bi-weekly refresher training was conducted during the data collection.

### Data management and analysis

Data analysis was performed using STATA 14.0 (StataCorp. 2015. Stata Statistical Software: Release 14. College Station, TX: StataCorp LP.).

Socio-demographic characteristics, e.g., age, educational attainment, and parity, were transformed into categorical variables. Due to small numbers, all other religions except ‘Muslim’ were grouped into one category and coded as ‘other’. We used the standard steps of principal component analysis to generate the socio-economic index of households that were interviewed, based on which the wealth quintile was generated [34, 35]. Household-level variables such as household possessions; materials used for the construction of floor, wall, and roof; drinking water source; toilet facilities; and ownership of land and domestic animals were used to generate this index.

Husbands’ involvement in care during pregnancy, childbirth, and following birth were considered as the main outcomes of interest in this paper. The following indicators, as reported by women, were used to denote husbands’ involvement:

I. Supporting women in self-care during pregnancy: Husbands were very supportive or somewhat supportive in assisting their wives in taking care of themselves during pregnancy.
II. Participation in birth preparedness and complication readiness (BPCR): Women discussed about BPCR with their husbands during most recent pregnancy
III. Being present during ANC contact: Husbands travelled with their wives when they sought ANC or they were present with their wives during an ANC contact.
IV. Being present during delivery: Husbands travelled with their wives for childbirth or they were present with their wives during childbirth.
V. Being present during postnatal care (PNC) visit: Husbands travelled with their wives when they sought PNC or they were present with their wives during PNC contact.
VI. Participation in newborn care: Husbands provided sufficient support to their wives to take care of the newborn

The above mentioned six indicators were given a score of 1 (yes) or 0 (no), and a composite score of 0–6 was created. Then the composite scores were categorised as non-active involvement (0–3 aspects) and active involvement (4–6 aspects). The composite scores were also categorized as 1–2, 3–4, 5–6 to understand the minute changes. Similar indicators were used to assess the preferences of the women regarding their husbands’ involvement. Frequency with confidence intervals for the above mentioned six indicators for women’s preferences and husbands’ reported involvement are presented in additional table 2. Three components of MNH-related decision-making were categorised as joint decision making (women and husband jointly), women only and husband only and prevalence was explored. Then, the decision making indicator was re-categorized as joint decision making (women and husband jointly) and not joint decision making (women only and husband only) for further exploration.

Chi-square tests were initially used to explore whether there was an association between explanatory variables (women’s preferences, husbands’ participation in decision-making, background characteristics, etc.) and outcomes of interest (husband’s involvement during pregnancy, labour, and childbirth). Then measures of association between different explanatory variables were tested through binary logistic regression. The effect of covariates and known confounders were adjusted by multiple logistic regression models for the following factors: age, education, religion, parity, husband’s living status, women’s involvement in income generating activities, wealth quintile, joint decision-making and women’s preferences [36–39]. All odds ratios (ORs) and adjusted odds ratios (AORs) were reported with 95% confidence intervals (CI). An association (OR or AOR) was considered significant if both the lower and the upper limit of the CI were more or less than one.

### Findings

Table 1 represents the background characteristics of the women who had a history of childbirth in the past 12-month preceding the survey. Around half of the women were under 25 years of age, whereas only 8% of the respondents were over 34 years of age. Sixty-two percent women had 6 or more years of schooling. Around 40% of women had completed primary schooling. Nearly all of the respondents were Muslim (97%). Around 70% of the respondents were multiparous. Just over half of the respondents reported that their husbands lived with them whereas the other half reported that their husbands lived in another place within the country or lived abroad. A negligible proportion of the women (less than 5%) had been involved in any kind of income-generating activities in the previous 12 months of the survey period.

**Table 1:** Background characteristics of women with a history of childbirth in the past 12-month period preceding the survey

Background characteristics	Women
	(***N*** = 1367)
	%
**Age**	
15–24 years	48.4
25 or more years	51.6
Mean age in years (SD)	25.3 (5.2)
**Education**	
0–5 years of schooling	37.5
6 or more years of schooling	62.5
Mean years of schooling (SD)	7.4 (2.8)
**Religion**	
Muslim	97.4
Others (Hindu/Christian etc.)	2.6
**Parity**	
Primipara	30.2
Multipara	69.8
**Husband living status**	
Lives with wife	55.5
Lives outside the home (within country or abroad)	44.5
**Women involved in income-generating activities**	
Yes	4.2
**Wealth quintile**	
Lowest	20.0
Second	19.9
Middle	20.0
Fourth	19.9
Highest	19.9

**Fig. 1:**
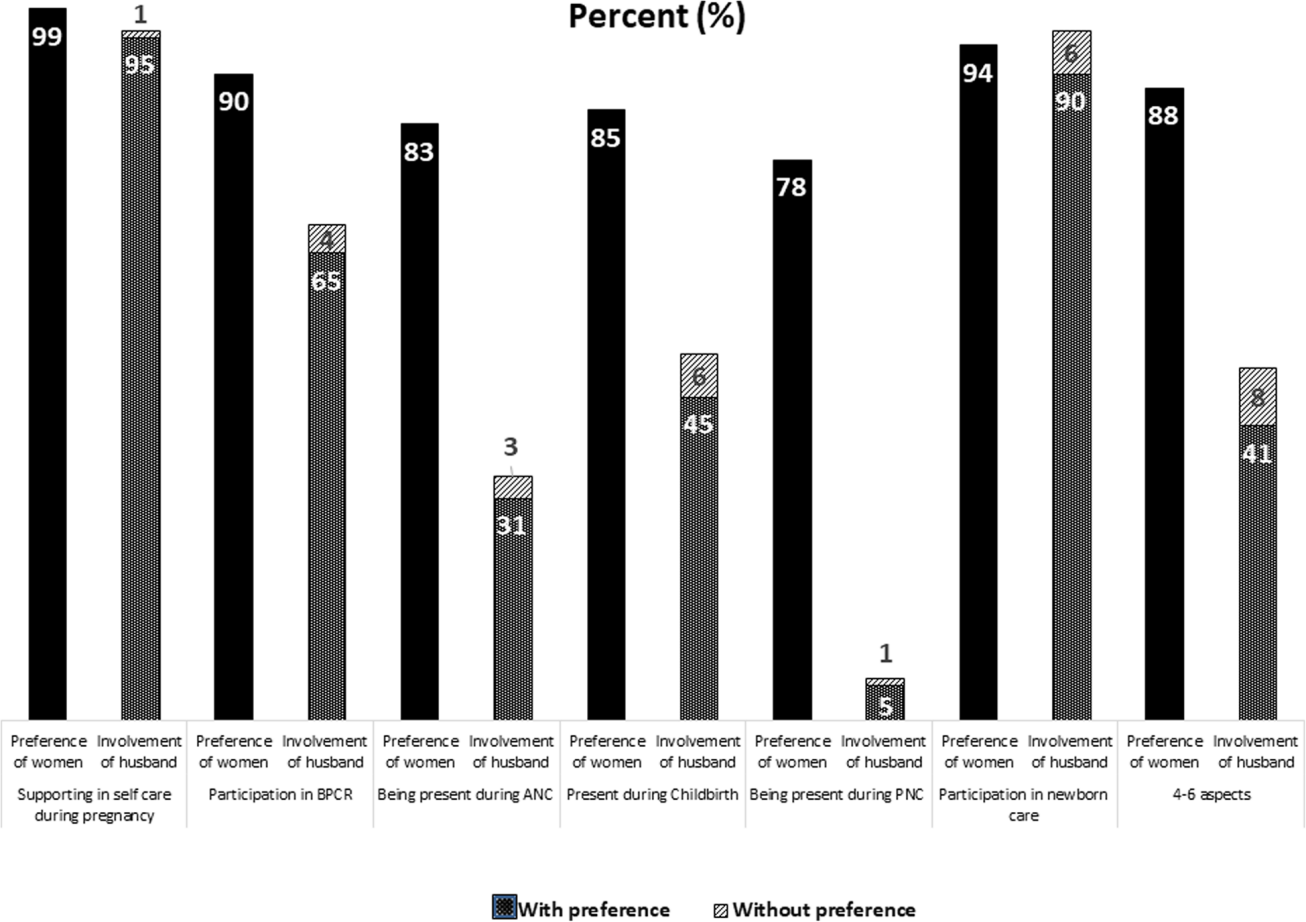
Involvement of husbands during pregnancy, childbirth, and following birth: preferences of women and engagement of husbands (*N* = 1367)

Figure 1 illustrates women’s preferences regarding the six aspects of their husbands’ involvement during pregnancy, childbirth and after birth and husbands’ reported involvement by the women. The first column shows the preference of women (i.e. whether or not she reports that she wanted her husband to be involved in that aspect of MNH care). The stacked bars in the second column presents husbands’ reported involvement, differentiating between involvement when the women reported desiring the particular category of involvement and when the women did not. Nearly all women reported that they wanted their husbands to support them during pregnancy and nearly all women reported that their husbands were very supportive or somewhat supportive in taking care of the women during pregnancy. However, difference between the proportion of women’s preference and involvement of husband is statistically significant (*P*-value 0.004). While more than 90% of women wanted their husbands’ involvement in BPCR, only 69% of husbands were reported to have participated in BPCR (*P*-value 0.000). Similarly, around 83% of women would have liked for their husbands to be present during an ANC visit, yet only one-third reported that their husband was present during any ANC contact. Most (85%) women reported that they would have liked their husband to be present during childbirth, but only half were present (*P*-value 0.000). Although 78% of women desired their husbands’ presence during PNC, only 6% of husbands accompanied their wives during this contact (*P*-value 0.000). The preference of women in favour of husbands’ involvement in newborn care was near-universal (96%), and around 88% husbands actively participated in newborn care practices (*P*-value 0.000). Altogether most women (88%) desired their husbands’ involvement in 4–6 aspects on MNH care, less than half of the husbands exhibited such engagement in the composite score. Moreover, there was significant difference between the proportion of women’s preference and the proportion husbands’ involvement in 4–6 aspects of MNH care (*P*-value 0.000). Across all aspects, a negligible proportion of husbands were involved in MNH care when it was not desired by their wives.

**Fig. 2:**
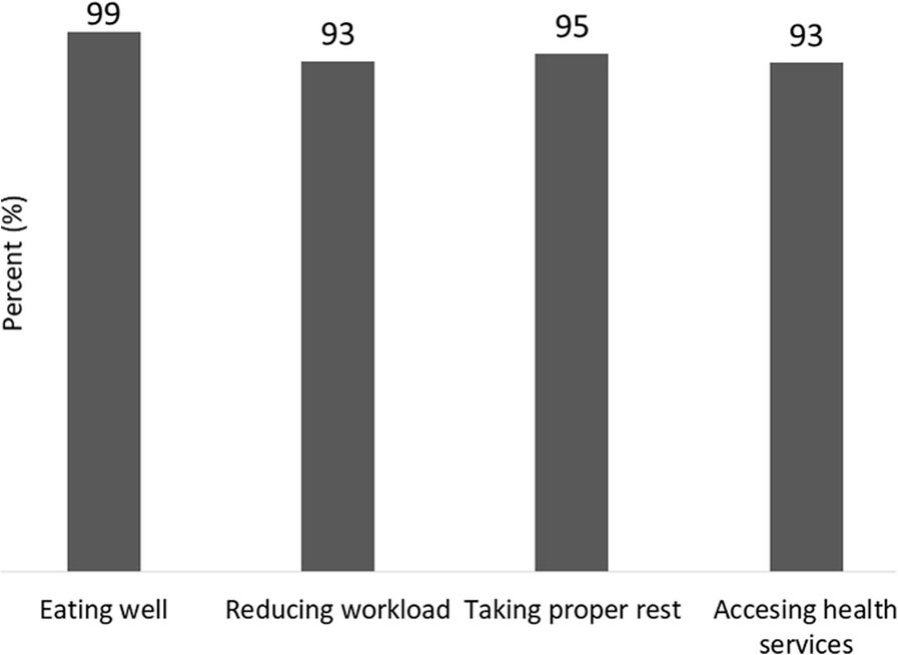
Specific support of husbands to women for self-care during pregnancy (*N* = 1323)

Figure 2 presents the types of physical or emotional support husbands provided to their wives for self-care during pregnancy as reported by women. Almost all of the women reported that their husbands supported them to eat well. Around 95% reported their husband’s support in reducing workload, taking proper rest, and accessing health services.

**Fig. 3:**
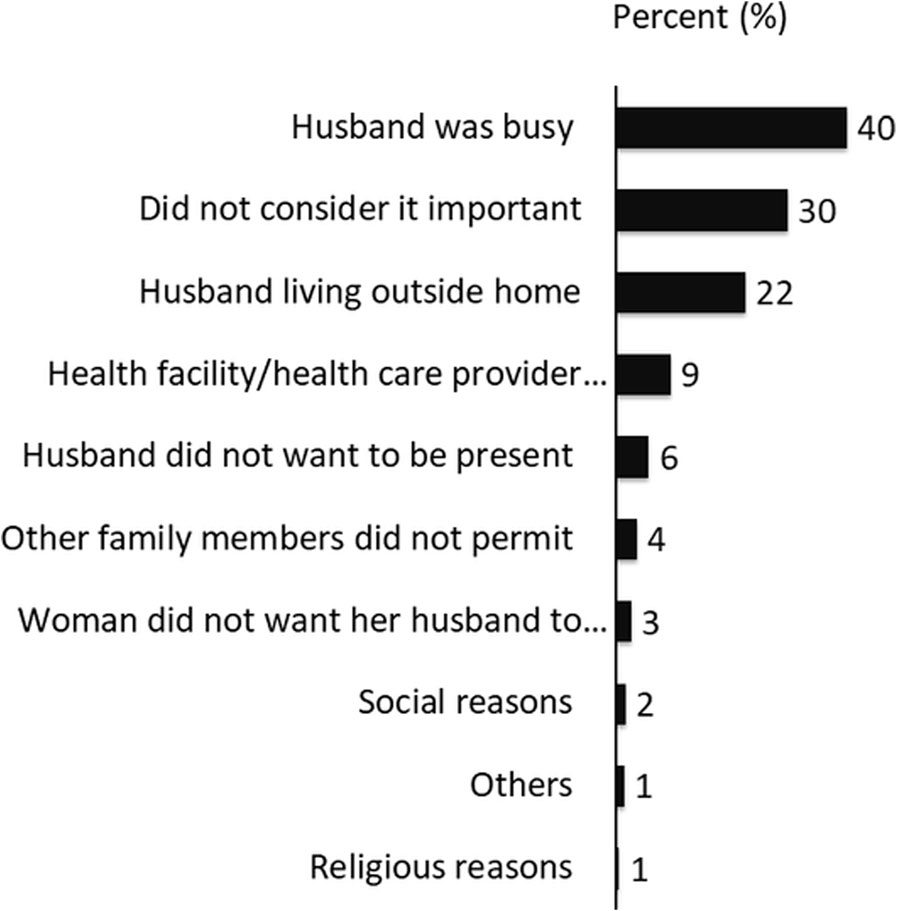
Reasons for husbands’ reported absence during childbirth (Multiple responses considered) (*N* = 917)

Figure 3 summarizes the reasons for husbands being physically absent during childbirth as reported by women. Out of the husbands who were not present during childbirth (*n* = 917), around 40% of them were busy with their work, and 30% did not consider their presence to be important. Around one-fourth were absent as they lived in other places (within the country or abroad). A further 9% could not be present as the health facility or the birth attendant did not permit them to be present during childbirth. Only 3% of women reported that they did not want their husbands’ to be present during the childbirth.

**Fig. 4:**
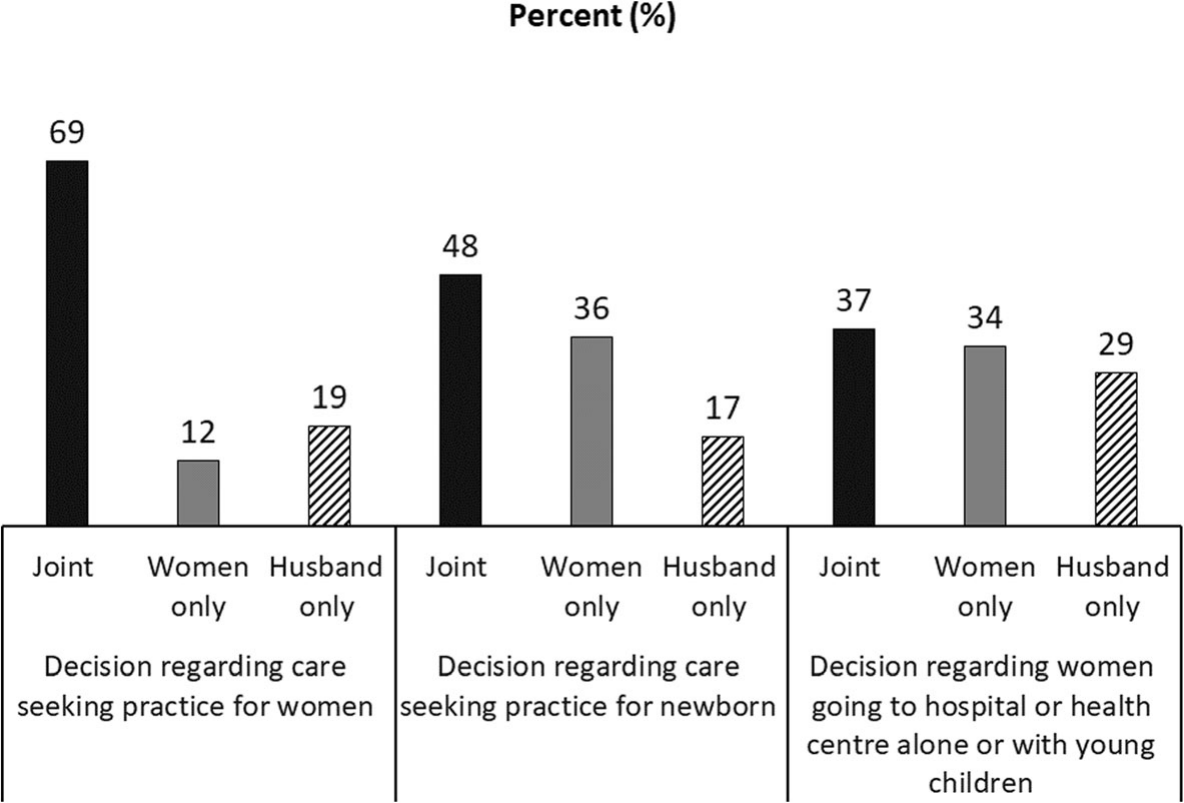
Husbands’ role and involvement in decision-making regarding MNH care-seeking (*N* = 1367)

Figure 4 illustrates husbands’ roles and participation in decision-making related to MNH care-seeking as reported by the women. Two-thirds of women reported taking joint decisions with their husbands (about their care), but around 20% of women reported that their husbands took decisions on their own. Although joint decision-making regarding newborn health was slightly lower (48%), a similar proportion of men (17%) took decisions regarding newborn health on their own. Around 37% of women took joint decisions with their husbands regarding whether the women could go outside home alone for MNH care. Around 29% of the husbands took this decision on their own.

Table 2 presents the relationship between husbands’ active involvement (four or more out of six aspects) with women’s preferences regarding husbands’ involvement and the husband’s roles in decision-making for MNH case-seeking practices controlling for the effect of other background characteristics. Women’s preferences (4–6 aspects) were strongly associated with husbands’ active involvement (AOR 2.94, *p* = 0.00). Similarly, if the husbands were jointly participating with their wives in decision-making for any of the three categories of MNH care seeking practices, the likelihood of their involvement in MNH care during their wives’ pregnancy, childbirth and after birth increases twofold (AOR 1.75, *p* = 0.00), compared to sole decision-making by the woman or sole decision-making by the man. Husbands who lived with their wives were also more likely to be involved in MNH care (AOR 5.07, *p* = 0.00).

**Table 2:** Relationship between different aspects and husbands’ involvement in MNH care during pregnancy, childbirth and after birth (*N* = 1367)

Background characteristics	Husbands involvement (4–6 aspects)				
	%	OR	***P*** value	AOR	***P*** value
**Age**					
15–24 years	48.5	Ref		Ref	
25 or more years	49.1	1.02	0.85	1.02	0.877
**Education**					
0–5 years of schooling	53.4	Ref		Ref	
6 or more years of schooling	48	0.81	0.084	0.99	0.928
**Religion**					
Muslim	48.7	Ref		Ref	
Others (Hindu/Christian etc.)	55.6	1.32	0.42	1.28	0.519
**Parity**					
Primipara	48.1	Ref		Ref	
Multipara	49.3	1.05	0.682	0.93	0.606
**Husband living status**					
Lives in within country or abroad	29.2	Ref		Ref	
Lives with wife	64.5	4.39	0.000	5.07	0.000
**Women involved in income-generating activities**					
No	48.8	Ref		Ref	
Yes	50.9	1.09	0.76	1.27	0.452
**Wealth quintile**					
Lowest	49.5	Ref		Ref	
Second	53.5	1.18	0.346	1.00	0.994
Middle	51.1	1.07	0.701	1.19	0.421
Fourth	46.3	0.88	0.465	1.21	0.394
Highest	44.1	0.81	0.212	1.25	0.338
**Joint decision** (any of 3 decision aspects taken jointly)					
No	40.4	Ref		Ref	
Yes	51.3	1.56	0.001	1.75	0.000
**Women’s preference (4–6 aspects)**					
No	27.3	Ref		Ref	
Yes	51.9	2.87	0.000	2.94	0.000

**Fig. 5:**
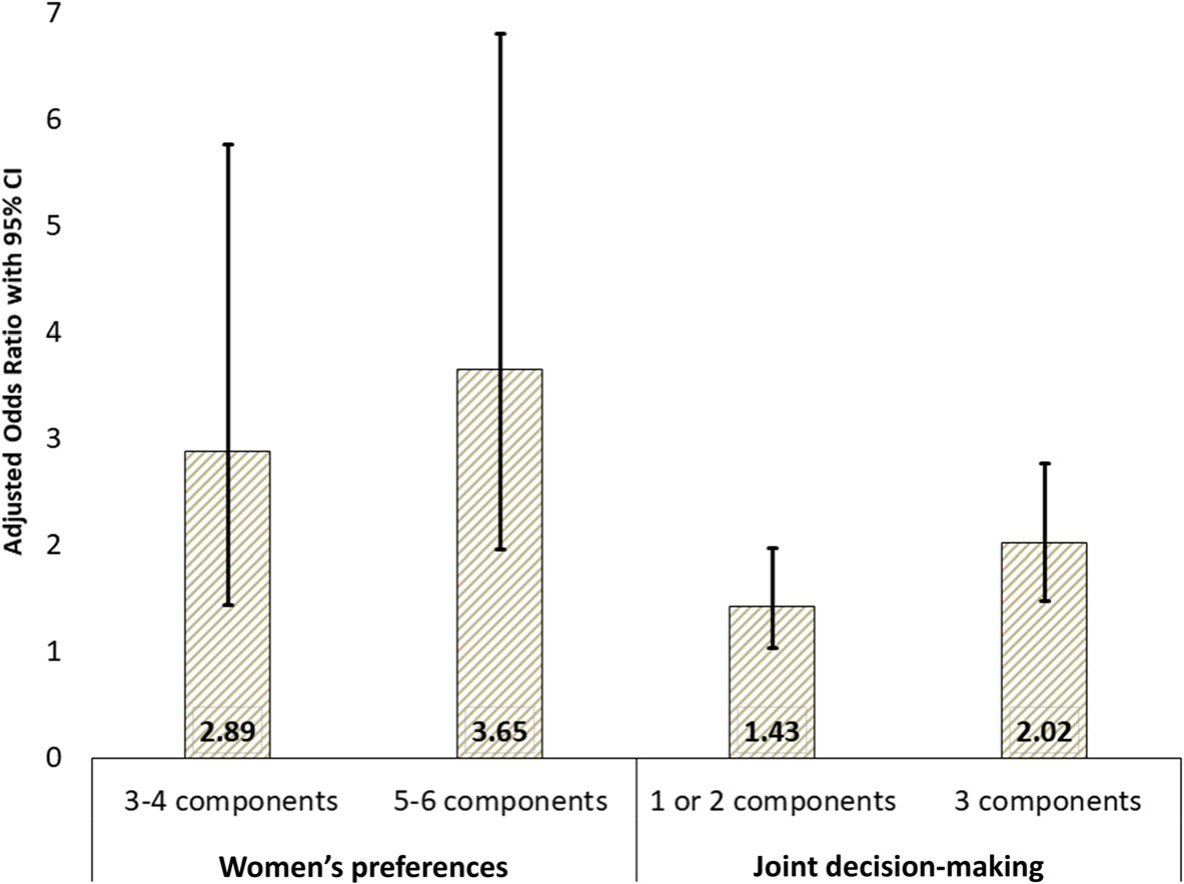
Associations between women’s preferences, the role of husband in decision-making for MNH care-seeking and husbands’ involvement in MNH care (*N* = 1367)

Figure 5 demonstrates the relationship between women’s preferences for their husbands’ involvement in MNH care and husbands’ reported involvement in MNH care. It also presents the relationship between joint decision-making for MNH care-seeking and husbands’ reported involvement in MNH care. The likelihood of women reporting their husbands were involved in 3–4 aspects of MNH care was 2.89 times higher when the women preferred their husbands to be involved in 3–4 aspects of MNH care. The likelihood increased to 3.65 times when the women preferred their husbands to be involved in 5–6 aspects. Similarly, the likelihood of husbands’ reported involvement in 3–6 aspects of MNH care was 1.43 times higher when they jointly participate in 1–2 categories of decision-making. The likelihood increased to 2.02 times when they jointly participate in all three categories.

## Discussion

WHO recommends involving men in MNH care and numerous initiatives have been implemented to promote their engagement through various approaches. Some studies have suggested that men’s involvement increases the likelihood of women’s use of skilled health services during pregnancy and birth [23, 40, 41]. However, few studies to date have examined the preferences of women regarding their husbands’/male partners’ involvement in MNH care and how husbands’ reported involvement compare to women’s preferences, and the decision-making dynamics within the household.

Bangladesh tends to be characterized by being a patriarchal society in which pregnancy and childbirth are typically relegated to the women’s sphere [18, 23, 36]. However, the results of our study indicate that women in rural Bangladesh would like to enjoy a high level of involvement of their husbands across various areas of MNH care which include assisting in self-care during pregnancy, participating in planning for birth and potential complications, accompanying to health services, and caring for the newborn. Such preferences of women in favour of the involvement of their husbands in pregnancy and childbirth may reflect the changing norms in rural Bangladesh, due at least in part to a rapid rise in women’s participation in formal education and transitions in the extended family structures in rural Bangladesh over the past few decades [42–44]. Exposure to new forms of information may have also contributed to changing women’s ideas about the potential roles of their husbands as access to smartphones in rural households has increased dramatically over the past decade [24].

Somewhat surprisingly, the women in our study overwhelmingly reported that they would like their husbands to be present during the childbirth. While this is a practice that has become common in many high-income contexts [45], it is often less common in low- and middle-income countries [46]. Previous studies conducted in such settings have revealed an ambivalence among women about the presence of their husbands at the time of birth [47, 48]. While some women have expressed the desire to have the support of their husbands/male partners at the time of birth, others have expressed the contrary, citing reasons such as embarrassment and discomfort, or the sentiment that the man would not be able to provide constructive support [49, 50]. Indeed, historically, women have benefitted overwhelmingly from their female social network at this time of birth. Our results indicate, however, that there is a desire on the part of women to include their husbands at this momentous occasion in rural Bangladesh.

Despite the high levels of preference regarding husband involvement in MNH care across all measured categories, the husbands’ reported involvement was significantly less than preferred. Men were most often involved in supporting women in self-care during pregnancy, such as in eating well, taking proper rest, and reducing workload during pregnancy, with nearly all women reporting that their husbands had supported them in caring for themselves during pregnancy. This high level of support during pregnancy is promising, suggesting that specific strategies could be employed to increase men’s awareness of positive care practices during pregnancy and a means of improving the care of women. Indeed, some studies in low-resource settings have demonstrated the MNH knowledge and awareness of men to be suboptimal [51]. Around two-thirds of husbands in our study were involved in planning for birth and potential complications which is consistent with some other studies conducted in Bangladesh [36, 52].

In contrast, rates of husband’s involvement in accompanying women to health services were lower. Reaching men through ANC can provide a valuable platform for educating men on care practices for women and newborns and the importance of seeking and utilising skilled health services. Only one-third of the respondents in our study were accompanied during at least one ANC contact by their husband. This low level is similar to what has been observed in Nepal [53], as well as in Ghana and other countries in Africa [54, 55]. Other studies in Myanmar [56] and Uganda [57] have found a higher level of male participation during ANC contacts. Indeed, this seems to be very context-specific and variable within regions and even countries. Our respondents reported lower levels of being accompanied by their husbands than that of which has been found in other areas of Bangladesh [36]. Men were much less likely to be present during PNC visits. Indeed, while the majority of women expressed that they would have liked for their husband to accompany them during PNC, only 5% were present during at least one PNC visit. However, this may primarily reflect the fewer number of opportunities for men to participate during these contacts and lower rates of women attending any PNC.

Other studies have identified various barriers discouraging men from accompanying women when they seek health services. These include social stigma, shyness and embarrassment, lack of men’s availability due to job responsibilities, and structural issues within the health services such as the availability, readiness and accessibility of health facilities, maternal health services which are not male-friendly and hospital policy restrictions [58–60]. Nearly 1 in 10 women mentioned that the health service provider did not permit the husband to be present during birth as a reason for their reported absence, indicating the potential benefit of a policy intervention to ensure that health services are welcoming and prepared to involve men.

Moreover, despite the high percentage of women expressing a desire for their husbands to be present at the time of birth, significantly fewer were present at this time. Women whose husbands were not present during birth provide various explanations for their reported absence. One-third of women in this situation felt like their husbands perceived their presence to be not necessary, suggesting a disconnect between the preferences of women and men. Around 70% of women mentioned that their husband was not available to be present at the time of birth. This is comparable to what has been found in Guatemala, where lack of the availability of men presented a key barrier to men’s participation at the time of birth [61]. This is perhaps not surprising, given that a high percentage of men among our study population who live outside of the home, either in another city in Bangladesh or abroad. Indeed trends toward the migration of men have a long history in Bangladesh, with many men travelling to urban areas, the Middle East and Europe to secure work [24, 62]. However, it is highly likely that men would return home around the time of childbirth, irrespective of their general residency status, which could explain why husbands are still able to be involved in MNH care, particularly in care for the newborn. Women’s preferences can also be influenced by the husband’s presence at home, which can eventually motivate their husbands’ MNH care practice.

In terms of discussions around maternal and newborn health in the household and decision-making, men played a relatively important role among our respondents, though not an exclusive role. In most cases, women reported themselves as the primary decision-maker or as participating in the decision. The most common scenario was joint decision/making, which is promising as couples’ decision-making has been found to be associated with increased care-seeking during pregnancy and birth in other studies [17, 52]. It is important to note that while in our study women reported joint decision-making, this should be interpreted with caution. Indeed, conceptualizations around what it means to be involved in decision-making can vary based on preferences and the specific societal, religious, economic factors. Therefore, it should not necessarily be interpreted that this reporting is reflective of women’s autonomy in decision making. However, while not a majority, many women reported that their husband took a decision without consulting them, which was not associated with women’s age, education, socio-economic status or parity. Further research should be conducted to better understand this phenomenon.

## Limitations

The study was cross-sectional and we are limited with the design to infer causality for the associations that we have presented in this paper. However, we have conducted multiple logistic regression to adjust for the potential effect of confounders and covariates. A prefixed model was used for these adjustments which was developed based on the literature review around men’s involvement in MNH. Another potential limitation of our study is recall bias as the childbirth experience may have influenced women’s reporting of their preferences and husbands’ practice in MNH care. Also, recall error is a limitation of this study, as we accepted up to 12 months of recall. We tried to minimise this by ensuring that the data collectors received extensive training to clarify different elements of the questionnaire to the respondents for their proper understanding and appropriate recall. Also, this recall period is much shorter than the 3 to 5 years recall period that is accepted by other surveys generating national estimates [24, 63, 64]. Another potential limitation could be social desirability bias, which we tried to address by recruiting data collectors from local communities who are familiar with the local culture, language and norms. Moreover, rigorous pre-testing of the questionnaire was done to address this bias.

This study has not considered any interaction effects in the regression model. However, strong effect modifiers could be present in this context. Further analyses should be conducted in order to understand the interaction effect of multiple factors to explain husbands’ MNH care practices. Furthermore, husbands’ involvement could also influence the decision-making dynamics and women’s preferences on MNH care practices. However, no reverse causality has been verified in this study. Additional investigation is needed to assess this circular relation.

Finally, it is important to recognise the limitations of what we can learn regarding gender and other social phenomena through such quantitative approaches. We call for future research to better understand these dynamics through qualitative, and particularly ethnographic, approaches.

## Conclusion

The results of our study suggest a picture which diverges from common representations of rural life in South Asia. Rather than being considered a women’s issue in which men are not preferred to be involved, we found that women consistently desired their husbands’ engagement in care during pregnancy, birth and following birth. We also found women’s preferences to be closely associated with husbands’ reported involvement in MNH care; that is to say, women’s preferences matter. Programmes and initiatives should acknowledge this and create an enabling environment and platform at home and in health facilities for husbands to participate in MNH care. Finally, the majority of women were engaged in MNH decision-making, either as a sole or joint decision-maker. Further research should explore in more depth intrahousehold decision-making dynamics and scrutinise the assumption of men as gatekeepers for perinatal care.

## Supplementary information

**Additional file 1: Table 1.** Summary of three sub-districts’ population, administrative structure and health systems details.

**Additional file 2: Table 2.** Involvement of husbands during pregnancy, childbirth, and following birth: preferences of women and engagement of husbands (*N* = 1367).

**Additional file 3**: Questionnaire used in this study. 

## Data Availability

The datasets generated during and/or analysed during the current study will be made available upon a valid request to the corresponding author (Ahmed Ehsanur Rahman, ehsanur@icddrb.org).

## Abbreviations

ANC: Antenatal Care
AOR: Adjusted Odds Ratio
BDHS: Bangladesh Demographic Health Survey
BMMS: Bangladesh Maternal Mortality Survey
BPCR: Birth Preparedness and Complication Readiness
CI: Confidence Interval
ERC: Ethical Review Committee
icddr, b: International Centre for Diarrhoeal Disease Research, Bangladesh
ICPD: International Conference on Population and Development
MICS: Multiple Indicator Cluster Surveys
MNH: Maternal and Newborn Health
OR: Odds Ratio
PNC: Postnatal Care
PPS: Probability Proportional to Size
RMNCH: Reproductive, Maternal, Newborn, and Child Health
RRC: Research Review Committee
WHO: World Health Organization

## References

[1] Greene ME, Biddlecom AE (2000). Absent and problematic men: demographic accounts of male reproductive roles. Popul Dev Rev.

[2] Bankole A, Singh S (1998). Correction: Couples' Fertility and Contraceptive Decision-Making in Developing Countries: Hearing the Man's Voice. International Family Planning Perspectives.

[3] Platiner M (1995). Status of Women under International Human Rights Law and the 1995 UN World Conference on Women, Beijing, China. Ky LJ.

[4] Davis J, Luchters S, Holmes W (2012). Men and maternal and newborn health: benefits, harms, challenges and potential strategies for engaging men.

[5] Santarelli C (2003). Working with individuals families and communities to improve maternal and newborn health.

[6] Walston N (2005). Challenges and opportunities for male involvement in reproductive health in Cambodia. Policy Project Washington, DC, USAID.

[7] Sternberg P, Hubley J (2004). Evaluating men's involvement as a strategy in sexual and reproductive health promotion. Health Promot Int.

[8] Dudgeon MR, Inhorn MC (2009). Men’s influences on women’s reproductive health: medical anthropological perspectives. Reconceiving the second sex: Men, masculinity, and reproduction.

[9] World Health Organization (2015). WHO recommendations on health promotion interventions for maternal and newborn health 2015: World Health Organization.

[10] Feldman Shelley (2001). Exploring Theories of Patriarchy: A Perspective from Contemporary Bangladesh. Signs: Journal of Women in Culture and Society.

[11] Moghadam VM (1994). Development and Patriarchy: The Middle East and North Africa in Economic and Demographic Transition 1994.

[12] Kandiyoti D (1988). Bargaining with patriarchy. Gend Soc.

[13] Senarath U, Gunawardena NS (2009). Women's autonomy in decision making for health care in South Asia. Asia Pac J Public Health.

[14] Karim R, Lindberg L, Wamala S, Emmelin M (2018). Men’s perceptions of Women’s participation in development initiatives in rural Bangladesh. Am J Mens Health.

[15] Mullany BC, Hindin MJ, Becker S (2005). Can women's autonomy impede male involvement in pregnancy health in Katmandu, Nepal?. Soc Sci Med.

[16] Comrie-Thomson L, Tokhi M, Ampt F, Portela A, Chersich M, Khanna R (2015). Challenging gender inequity through male involvement in maternal and newborn health: critical assessment of an emerging evidence base. Cult Health Sex.

[17] Story WT, Burgard SA (2012). Couples’ reports of household decision-making and the utilization of maternal health services in Bangladesh. Soc Sci Med.

[18] Story WT, Burgard SA, Lori JR, Taleb F, Ali NA, Hoque DE (2012). Husbands' involvement in delivery care utilization in rural Bangladesh: a qualitative study. BMC Pregnancy Childbirth.

[19] Sarker Bidhan Krishna, Rahman Musfikur, Rahman Tawhidur, Hossain Jahangir, Reichenbach Laura, Mitra Dipak Kumar (2016). Reasons for Preference of Home Delivery with Traditional Birth Attendants (TBAs) in Rural Bangladesh: A Qualitative Exploration. PLOS ONE.

[20] Banik BK (2016). Barriers to access in maternal healthcare services in the northern Bangladesh. South East Asia J Public Health.

[21] Mahumud RA, Alamgir NI, Hossain M, Baruwa E, Sultana M, Gow J (2019). Women’s preferences for maternal healthcare services in Bangladesh: evidence from a discrete choice experiment. J Clin Med.

[22] Hossain M, Kabir M (2001). Purdah, mobility and women's empowerment and reproductive behaviour in rural Bangladesh. Social Change.

[23] Chattopadhyay A (2012). Men in maternal care: evidence from India. J Biosoc Sci.

[24] National Institute of Population Research and Training (NIPORT), Mitra and Associates, ICF International (2016). Bangladesh Demographic and Health Survey 2014.

[25] Bangladesh; GoTPsRo (2018). Bangladesh National Strategy for Maternal Health 2015–2030.

[26] El Arifeen S, Christou A, Reichenbach L, Osman FA, Azad K, Islam KS (2013). Community-based approaches and partnerships: innovations in health-service delivery in Bangladesh. Lancet.

[27] Das P, Horton R (2013). Bangladesh: innovating for health. Lancet.

[28] Char A (2011). Male involvement in family planning and reproductive health in rural Central India: Tampere University press.

[29] Bangladesh Bureau of Statistics (BBS) (2013). District Statistics 2011 Brahmanbaria.

[30] Gordis L. Epidemiology Fourth Edition. Philadelphia: Elsevier Saunders Publication; 2005.

[31] Croft TN, Marshall AMJ, Allen CK, et al. "Guide to DHS statistics." Rockville: ICF; 2018.

[32] Lavrakas P (2019). Encyclopedia of survey research methods.

[33] Skinner CJ (2014). Probability proportional to size (PPS) sampling. Wiley StatsRef: Statistics Reference Online.

[34] Vyas S, Kumaranayake L (2006). Constructing socio-economic status indices: how to use principal components analysis. Health Policy Plan.

[35] Wold S, Esbensen K, Geladi P (1987). Principal component analysis. Chemom Intell Lab Syst.

[36] Rahman AE, Perkins J, Islam S, Siddique AB, Moinuddin M, Anwar MR (2018). Knowledge and involvement of husbands in maternal and newborn health in rural Bangladesh. BMC Pregnancy Childbirth.

[37] Ibrahim MS, MaB S, Idris SH, Asuke S, Yahaya SS, Olorukooba AA (2014). Effect of a behavioral intervention on male involvement in birth preparedness in a rural community in Northern Nigerian. Ann Nig Med.

[38] Mersha AG (2018). Male involvement in the maternal health care system: implication towards decreasing the high burden of maternal mortality. BMC Pregnancy Childbirth.

[39] Craymah Joshua Panyin, Oppong Robert Kwame, Tuoyire Derek Anamaale (2017). Male Involvement in Maternal Health Care at Anomabo, Central Region, Ghana. International Journal of Reproductive Medicine.

[40] Yargawa J, Leonardi-Bee J (2015). Male involvement and maternal health outcomes: systematic review and meta-analysis. J Epidemiol Community Health.

[41] Zamawe C, Banda M, Dube A (2015). The effect of mass media campaign on Men’s participation in maternal health: a cross-sectional study in Malawi. Reprod Health.

[42] Statistics BBO (2011). Statistical yearbook of Bangladesh. Statistics Division, Ministry of Planning, Dhaka, Government of the People’s Republic of Bangladesh.

[43] Statistics BBO (2001). Statistical yearbook of Bangladesh. Statistics Division, Ministry of Planning, Dhaka, Government of the People’s Republic of Bangladesh.

[44] National Institute of Population Research and Training (NIPORT), Mitra and Associates, ICF International (2013). Bangladesh Demographic and Health Survey 2011.

[45] Bohren MA, Berger BO, Munthe-Kaas H, Tunçalp Ö (2019). Perceptions and experiences of labour companionship: a qualitative evidence synthesis. Cochrane Database Syst Rev.

[46] Hodnett ED, Gates S, Hofmeyr GJ, Sakala C (2012). Continuous support for women during childbirth. Cochrane Database Syst Rev.

[47] Lewis S, Lee A, Simkhada P (2015). The role of husbands in maternal health and safe childbirth in rural Nepal: a qualitative study. BMC Pregnancy Childbirth.

[48] Adeniran AS, Aboyeji AP, Fawole AA, Balogun OR, Adesina KT, Adeniran PI (2015). Male Partner’s role during pregnancy, labour and delivery: expectations of pregnant women in Nigeria. Int J Health Sci (Qassim).

[49] Afulani P, Kusi C, Kirumbi L, Walker D (2018). Companionship during facility-based childbirth: results from a mixed-methods study with recently delivered women and providers in Kenya. BMC Pregnancy Childbirth.

[50] Kabakian-Khasholian T, Portela A (2017). Companion of choice at birth: factors affecting implementation. BMC Pregnancy Childbirth.

[51] Bougangue B, Ling HK (2017). Male involvement in maternal healthcare through community-based health planning and services: the views of the men in rural Ghana. BMC Public Health.

[52] Islam S, Perkins J, Siddique MAB, Mazumder T, Haider MR, Rahman MM (2018). Birth preparedness and complication readiness among women and couples and its association with skilled birth attendance in rural Bangladesh. PLoS One.

[53] Bhatta DN (2013). Involvement of males in antenatal care, birth preparedness, exclusive breast feeding and immunizations for children in Kathmandu, Nepal. BMC Pregnancy Childbirth.

[54] Ganle JK, Dery I (2015). ‘What men don’t know can hurt women’s health’: a qualitative study of the barriers to and opportunities for men’s involvement in maternal healthcare in Ghana. Reproductive Health.

[55] Jennings L, Na M, Cherewick M, Hindin M, Mullany B, Ahmed S (2014). Women’s empowerment and male involvement in antenatal care: analyses of demographic and health surveys (DHS) in selected African countries. BMC Pregnancy Childbirth.

[56] Wai KM, Shibanuma A, Oo NN, Fillman TJ, Saw YM, Jimba M (2015). Are husbands involving in their spouses’ utilization of maternal care services?: a cross-sectional study in Yangon, Myanmar. PloS one.

[57] Tweheyo R, Konde-Lule J, Tumwesigye NM, Sekandi JN (2010). Male partner attendance of skilled antenatal care in peri-urban Gulu district, northern Uganda. BMC Pregnancy Childbirth.

[58] Barker G (2000). Gender equitable boys in a gender inequitable world: reflections from qualitative research and programme development in Rio de Janeiro. Sex Relatsh Ther.

[59] Ibrahim SM, Isa B, Ibrahim HA, Kullima AA, Geidam AD. Are men adequately involved in maternal healthcare? Int J Curr Res. 2016;8(8):36602–6.

[60] Davis J, Vyankandondera J, Luchters S, Simon D, Holmes W (2016). Male involvement in reproductive, maternal and child health: a qualitative study of policymaker and practitioner perspectives in the Pacific. Reprod Health.

[61] Carter M (2002). Husbands and maternal health matters in rural Guatemala: wives’ reports on their spouses’ involvement in pregnancy and birth. Soc Sci Med.

[62] Farid KS, Mozumdar L, Kabir MS, Hossain KB (2009). Trends in international migration and remittance flows: case of Bangladesh. J Bangladesh Agric Univ.

[63] MEASURE Evaluation, DHS. "ICF International. Guide to DHS Statistics: Demographic and Health Surveys Methodology". Rockville: ICF; 2006.

[64] National Institute of Population Research and Training (NIPORT), International Centre for Diarrhoeal Disease Research Bangladesh icddr b, MEASURE Evaluation. Bangladesh Maternal Mortality and Health Care Survey 2016: Preliminary Report. Dhaka and Chapel Hill: NIPORT, icddr,b, and MEASURE Evaluation; 2017.

