# Supplementary material for: What do women want? An analysis of preferences of women, involvement of men, and decision-making in maternal and newborn health care in rural Bangladesh
Source: BMC Pregnancy Childbirth. 2020 Mar 18;20:169. doi: 10.1186/s12884-020-2854-x (PMC7079480; doi:10.1186/s12884-020-2854-x)
Supplement: Supplementary file 1 — Additional file 1: Table 1. Summary of three sub-districts’ population, administrative structure and health systems details. [file 12884_2020_2854_MOESM1_ESM.docx]

Additional Table 1: Summary of three sub-districts’ population, administrative structure and health systems details

|  | **Sarail** | **Kasba** | **Bijoynagar** |
| --- | --- | --- | --- |
|  | **#** | **#** | **#** |
| Population | 327311 | 331937 | 267041 |
| Union (Smallest administrative unit) | 09 | 10 | 10 |
| Village | 141 | 209 | 225 |
| Average Household (HH) size | 5.36 | 5.23 | 5.28 |
| Primary Care Referral Hospital-Upazila Health Complex (50 beds) | 1 | 1 | 1 |
| Primary Care Facility-Union Health and Family Welfare Centre | 4 | 7 | 10 |
| Primary Care Outpost-Community Clinic | 21 | 32 | 28 |
| Community Health Worker-Health Assistant | 44 | 32 | 25 |
| Community Health Worker-Family Welfare Assistant | 50 | 50 | 31 |
