# Supplementary material for: What do women want? An analysis of preferences of women, involvement of men, and decision-making in maternal and newborn health care in rural Bangladesh
Source: BMC Pregnancy Childbirth. 2020 Mar 18;20:169. doi: 10.1186/s12884-020-2854-x (PMC7079480; doi:10.1186/s12884-020-2854-x)
Supplement: Supplementary file 2 — Additional file 2: Table 2. Involvement of husbands during pregnancy, childbirth, and following birth: preferences of women and engagement of husbands (N = 1367). [file 12884_2020_2854_MOESM2_ESM.docx]

Additional Table 2: Involvement of husbands during pregnancy, childbirth, and following birth: preferences of women and engagement of husbands (N=1367)

|  | | Frequency | Percentage | Confidence intervals | |
| --- | --- | --- | --- | --- | --- |
|  |  |  |  | Lower | Upper |
| Supporting in self-care during pregnancy | Preference of women | 1346 | 98.5% | 97.7% | 99.0% |
|  | Involvement of husband | 1323 | 96.8% | 95.7% | 97.6% |
| Participation in BPCR | Preference of women | 1235 | 90.3% | 88.7% | 91.8% |
|  | Involvement of husband | 948 | 69.3% | 66.8% | 71.7% |
| Being present during ANC | Preference of women | 1137 | 83.2% | 81.1% | 85.1% |
|  | Involvement of husband | 464 | 33.9% | 31.5% | 36.5% |
| Present during Childbirth | Preference of women | 1159 | 84.8% | 82.8% | 86.6% |
|  | Involvement of husband | 699 | 51.1% | 48.5% | 53.8% |
| Being present during PNC | Preference of women | 1069 | 78.2% | 75.9% | 80.3% |
|  | Involvement of husband | 72 | 5.3% | 4.2% | 6.6% |
| Participation in newborn care | Preference of women | 1279 | 93.6% | 92.1% | 94.7% |
|  | Involvement of husband | 1314 | 96.3% | 95.2% | 97.2% |
| 4-6 aspects | Preference of women | 1202 | 87.9% | 86.1% | 89.6% |
|  | Involvement of husband | 667 | 48.9% | 46.2% | 51.6% |
