# Supplementary material for: What do women want? An analysis of preferences of women, involvement of men, and decision-making in maternal and newborn health care in rural Bangladesh
Source: BMC Pregnancy Childbirth. 2020 Mar 18;20:169. doi: 10.1186/s12884-020-2854-x (PMC7079480; doi:10.1186/s12884-020-2854-x)
Supplement: Supplementary file 3 — Additional file 3: Questionnaire used in this study. [file 12884_2020_2854_MOESM3_ESM.pdf]

Testing a package of health promotion and community engagement interventions based on the WHO's Individuals, Families and Communities (IFC) Framework for improving MNH in selected upazilas of Bangladesh.
[Protocol No.17088]

Module W

Maternal and Newborn Module

১ লা জানুয়ারী, ২০১৭ থেকে ৩১ অক্টোবর ২০১৭ এর মধ্যে গর্ভ ফলাফল হয়েছে এমন মহিলাকে এই প্রশ্নগুলো করতে হবে।

ALL QUESTIONS ARE TO BE ADDRESSED TO WOMEN WITH A PREGNANCY OUTCOME FROM 01 JANUARY 2017 TO 31 OCTOBER 2017

	Name	Code	
**DISTRICT**	Brahmanbaria	12	সাক্ষাৎকার শুরু হবার সময়<br>[Interview start time]<br>*24 hours time*<br>|__|__| : |__|__|
**UPAZILA**		☐☐	
**UNION**		☐☐	
**CLUSTER**		☐☐☐	
**VILLAGE**		☐☐☐☐☐☐	
**BARI**		☐☐☐	সাক্ষাৎকার শেষ করার সময়<br>[Interview end time]<br>|__|__| : |__|__|
**HOUSEHOLD ID**		☐☐	
**WOMAN**		☐☐	
**HUSBAND**			
**CHILD**		☐☐	
**ADDRESS**			

Interviewer's Visit

	Visit 1	Visit 2	Visit 3	Final Visit	
**Date**	|__|__|-|__|__| 2018	|__|__|-|__|__| 2018	|__|__|-|__|__| 2018	**Date**	|__|__|-|__|__|-|__|__|
**Interviewer's Name**				**Interviewer's code**	|__|__|__|__|__|
**Result code***	|__||__|	|__||__|	|__||__|	**Result code**	|__|__|
**Next Visit**	|__|__|-|__|__| 2018	|__|__|-|__|__| 2018	|__|__|-|__|__| 2018	**Total # of visits**	|__|

Fill up the form if the recently delivered woman belongs to the following category:

☐ History of a live birth from 01 January 2017 to 31 October 2017

মন্তব্য (Comments)

***Result Codes**
01. Interview complete
02. Interview incomplete
03. Respondant absent
04. Refused to give interview
05. Migrated
06. Household destroyed
07. Others____________________

**Supervision**	নাম<br>**Name**	কোড<br>**Code**	তারিখ<br>**Date**
**Reviewed by QC Team**		|__|__|__||__||__|	|__|__|-|__|__| 2018
**Reviewed by FRO**		|__|__||__||__||__|	|__|__|-|__|__| 2018
**Data Entered by**		|__|__||__||__|	|__|__|-|__|__| 2018

Consent form for woman with a recent birth history

প্রধান গবেষক: আহমেদ এহসানুর রহমান

গবেষনাকারী প্রতিষ্ঠান: আই সি ডি ডি আর, বি (কলেরা হাসপাতাল), বাংলাদেশ সরকারের স্বাস্থ্য মন্ত্রনালয়ের এনএনএইচপি এন্ড আইএমসিআই সেকশন এবং ইডিএম এর যৌথ প্রচেষ্টায় পরিচালিত একটি গবেষণা প্রকল্প।

গবেষণার উদ্দেশ্য

আস্‌সালামুআলাইকুম/ আদাব, আমরা আই সি ডি ডি আর, বি, (কলেরা হাসপাতাল) থেকে এসেছি। উপজেলা পর্যায়ে মা, নবজাতক ও শিশু স্বাস্থ্য সেবার মান উন্নয়নে বাংলাদেশ সরকারকে সাহায্য করার উদ্দেশ্যে আই সি ডি ডি আর, বি, বাংলাদেশ সরকারের স্বাস্থ্য মন্ত্রনালয়ের এনএনএইচপি এন্ড আইএমসিআই সেকশন এবং ইডিএম এর এর যৌথ উদ্যোগে "ইন্ডিভিজ্যুয়ালস, ফ্যামিলিজ এন্ড কমিউনিটিজ প্রোগ্রাম ইন বাংলাদেশ ফর ইম্প্রুভমেন্ট অফ ম্যাটারনাল এন্ড নিউবর্ণ হেলথ" নামে ব্রাক্ষণবাড়িয়া জেলায় এ গবেষণা প্রকল্প চালাচ্ছে। এ কারণে আমরা ব্রাক্ষণবাড়িয়া জেলার মোট তিনটি উপজেলা সরাইল, কসবা ও বিজয়নগরে একটি জরিপ করছি। গবেষণাটির উদ্দেশ্য হলো এই জায়গাগুলোতে মা ও নবজাতকের স্বাস্থ্যের সার্বিক মান উন্নয়ন। এই গবেষণায় প্রাপ্ত তথ্যগুলো বাংলাদেশ সরকারকে আরো ভালো স্বাস্থ্য সেবা দিতে সহায়তা করবে।

আপনাকে কেন এই গবেষণায় অন্তর্ভুক্ত করা হল

এই গবেষণাটি মা ও নবজাতকের সেবার ক্ষেত্রে অংশগ্রহণ ও ব্যবহারে ব্যাক্তি, পরিবার ও কমিউনিটির সক্ষমতা বৃদ্ধির লক্ষে পরিচালিত হচ্ছে। তাই এ সম্পর্কিত অত্র এলাকার বর্তমান অবস্থা কি তা জানা প্রয়োজন। সে লক্ষেই এই জরিপ টি পরিচালিত হচ্ছে। আপনি যেহেতু একজন মা এবং এই এলাকায় স্থায়ীভাবে বসবাস করেন তাই আপনি গর্ভকালীন, প্রসব কালীন, প্রসব পরবর্তী ও নবজাতকের বিভিন্ন সেবা সর্ম্পকে অবহিত আছেন। তাই দৈব চয়নের মাধম্যে আপনাকে বাছাই করা হয়েছে। এই গবেষণায় আমাদের সহায়তার জন্য আমরা আপনাকে আমন্ত্রন জানাচ্ছি।

কার্যপদ্ধতি ও প্রক্রিয়া

যদি আপনি এই গবেষণায় অংশগ্রহন করতে রাজী থাকেন তাহলে আমরা আপনাকে কিছু প্রশ্ন করব যেমন: গর্ভকালীন, প্রসব কালীন, প্রসব পরবর্তী ও নবজাতকের বিভিন্ন সেবা গ্রহন, এ বিষয়ে আপনার স্বামী /পরিবার /এলাকার লোকজনের দায়িত্ব এবং আপনার আর্থসামাজিক অবস্থা।

আপনি যদি রাজি থাকেন তাহলে আমরা একটি সাক্ষাতকার নিব, যদি সম্ভব হয় তাহলে সুষ্ঠুভাবে সাক্ষাতকার সম্পন্ন করার জন্য পরবর্তীতে আমাদের আসতে হতে পারে। সাক্ষাতকার চলাকালীন সময়ে আমরা প্রশ্নাবলীগুলো পূরণ করব এবং কিছু মন্তব্য লিখে রাখব। এই জন্য ৩০ - ৪০ মিনিটের মত সময় লাগতে পারে।

ঝুঁকি এবং সুবিধা

এই গবেষণায় অংশগ্রহণের জন্য আপনার ঝুঁকির সম্ভাবনা খুবই কম। আপনি যে তথ্য দিবেন সেগুলো সম্পূর্ণ গোপন রাখা হবে এবং শুধুমাত্র গবেষণার কাজে ব্যবহার করা হবে। এই গবেষণায় অংশগ্রহন করে আপনি সরাসরি লাভবান নাও হতে পারেন কিন্তু আপনার দেওয়া তথ্যের কার্যকারীতা ব্যাপক যা আপনার এলাকায় আরো ভালোভাবে কার্যক্রম চালাতে সহায়তা করবে যা পরবর্তীতে বাংলাদেশ ও অন্যান্য স্থানে মা, নবজাতক ও শিশুদের স্বাস্থ্যের উন্নয়নে কাজে লাগবে।

গোপনীয়তা এবং বিশ্বস্ততা

আপনাকে নিশ্চয়তা প্রদান করা হচ্ছে যে, আপনাকে চিহ্নিত করার মত সকল তথ্য বিশ্বস্ততার সাথে সম্পূর্ণ গোপন রাখা হবে। আপনি আমাদেরকে যে সকল তথ্য প্রদান করবেন তার সবকিছুই গোপন থাকবে এবং তালাচাবি দিয়ে আটকানো থাকবে। গবেষণার গবেষকবৃন্দ, এবং বিশেষ প্রয়োজনে আইন-শৃংখলা রক্ষাকারী সংস্থা ছাড়া অন্য কেউই আপনার দেওয়া তথ্য জানতে পারবে না। গবেষণার তথ্যগুলো হয়ত বিশ্লেষণের জন্য দেশের বাইরে পাঠানো হতে পারে (কেবলমাত্র যখন প্রযোজ্য), তবে আপনাকে চিহ্নিত করার মত সকল তথ্য খুবই সাবধানতার সাথে ব্যবহার করা হবে, এবং নগণ্য সংখ্যক ব্যক্তির বাইরের কেউই তা জানতে পারবেন না। এমন স্থানে আপনার সাক্ষাৎকার নেয়া হবে যাতে অন্য কেউ কথোপকথন শুনতে না পারে। এই গবেষণা সংক্রান্ত যে কোন প্রশ্নের উত্তর আমরা সানন্দে দেব।

তথ্যের ভবিষ্যৎ ব্যবহার

এই গবেষণায় প্রাপ্ত তথ্য পরবর্তীতে দেশে বা দেশের বাইরে অন্য প্রতিষ্ঠানে পুনঃনিরীক্ষণের জন্য পাঠানো হতে পারে। সেক্ষেত্রেও আপনার গোপনীয়তা ও ব্যক্তিগত তথ্য সংরক্ষণ করা হবে যাতে আপনাকে সনাক্ত করা সম্ভব না হয়।

স্বেচ্ছা- সম্মতি

আপনি এই গবেষণা কার্যক্রমে অংশগ্রহণ করবেন কি করবেন না তা সম্পূর্ণ আপনার সিদ্ধান্ত। অংশগ্রহণ করলেও এই গবেষণা কার্যক্রম চলাকালীন যেকোন সময় আপনি নিজেকে প্রত্যাহার করতে পারেন। এর জন্য আপনাকে কোন কারণ দর্শাতে হবে না। এমনকি আপনি যদি অংশগ্রহণ নাও করেন তারপরেও আপনার বা আপনার পরিবারের অন্য যে কোন সদস্য তেমন স্বাস্থ্যসেবাই পাবেন যেমনটি আগে পেতেন। আমরা আপনার কাছে আবারও আসতে পারি। সেক্ষেত্রেও দৈব বিবেচনাতেই আসবো। এমন কোন বাধ্যবাধকতা নাই যে প্রথমবার অংশগ্রহণ করলে পরবর্তীতেও অংশগ্রহণ করতে হবে।

ক্ষতিপূরণের নীতি

আগেই বলা হয়েছে যে এই কাজে অংশগ্রহণের সিদ্ধান্ত একান্তই আপনার এবং এই গবেষণায় অংশগ্রহণের জন্য আপনাকে কোন প্রকার খরচ প্রদান করা হবে না।

যোগাযোগের ঠিকানা

এই গবেষণা কার্যক্রম সংক্রান্ত আপনার যদি কোন প্রশ্ন থেকে থাকে অথবা আপনার কোন অভিযোগ থাকে তাহলে প্রধান গবেষকের সাথেযোগাযোগ করতে পারেন। যোগাযোগের ঠিকানা আহমেদ এহসানুর রহমান, মেটারনাল এন্ড চাইল্ড হেল্থ ডিভিশন, ফোন: +৮৮০৯৬৬৬৭৭১১০০, এক্সটেনশন: ৩৮২৯, মোবাইল ঃ ০১৭২৭০১০০৫০. এছাড়াও আপনি যোগাযোগ করতে পারেন, আই সি ডি ডি আর, বি, সেকরেটারিয়েট, মি: এম এ সালাম খান, (ফোন: +৮৮ ০২ ৯৮২৭০৮৪ অথবা পিএবিএক্স +৮৮ ০২ ৯৮২৭০০১-১০, এক্সটেনশন: ৩২০৬)

আপনি এই গবেষণায় অংশ নিতে রাজী থাকলে সেটা স্বাক্ষর করে বা বুড়ো আঙ্গুলের ছাপ দিয়ে সম্মতি দিন।
সহযোগিতার জন্য ধন্যবাদ।
অংশগ্রহনকারী: উপরোক্ত তথ্যাবলী আমার কাছে যথাযথভাবে বর্ণিত হয়েছে এবং আমি বর্ণিত তথ্যাবলী বুঝতে পেরেছি

____
তথ্য প্রদানকারীর স্বাক্ষর   তারিখ

____
প্রধান গবেষকের স্বাক্ষর অথবা তাঁর প্রতিনিধি   তারিখ

Section A: Maternal Care
মাতৃত্বকালীন সেবা

NO	Questions And Filters	Coding Categories	Skip
w01.	প্রতিটি মানুষের কিছু মৌলিক অধিকার রয়েছে, যেমন: খাদ্য, বস্ত্র, বাসস্থান, চিকিৎসা ও শিক্ষা। এই মৌলিক অধিকার ছাড়াও গর্ভকালীন, প্রসবকালীন এবং প্রসবপরবর্তী সময়ে একজন মহিলার বিশেষ অধিকার আছে বলে আপনি মনে করেন কি?<br>Every human being has some basic rights, such as: food, clothing, shelter, health and education. Do you think/feel apart from these a woman has special/particular rights during pregnancy, childbirth and afterbirth?	হ্যাঁ Yes.. 1<br>না No... 2<br>জানি না/ মনে নাই Don't know/Can't remember.................... 9	**2/9 → w13**

NO	Questions And Filters		Unprompted Yes	Prompted Yes	No	Skip
w02.	গর্ভকালীন, প্রসবকালীন এবং প্রসবপরবর্তী সময়ে একজন মহিলার বিশেষ এ অধিকারগুলো কি কি?<br><u>[জিজ্ঞেস করুন, আরও কিছু? উত্তরদাতার নিজে থেকে দেয়া সবগুলো উত্তর প্রথমেই বৃত্তায়িত করুন। উত্তরগুলো পড়ে শুনাবেন না। একাধিক উত্তর হতে পারে। এরপর বাকি উত্তরগুলো পড়ে শুনান]</u><br>What are these special rights during pregnancy, childbirth and afterbirth?<br><u>[Do not read out the options. Keep asking what else. Record all the answers first. Then read out the remaining options]</u>	মাতৃ ও নবজাতকের মানসম্মত স্বাস্থ্যসেবা পাওয়ার অধিকার<br>Right to access quality maternal and newborn health services ....A	1	2	3	
		নিজের জন্য ও নবজাতকের জন্য চিকিৎসা নেওয়ার ক্ষেত্রে স্বাধীনভাবে সিদ্ধান্ত নেয়ার অধিকার<br>Right to make decisions to seek health services for herself or newborn ....B	1	2	3	
		স্বাস্থ্যসেবা প্রদানকারীর কাছ থেকে সম্মানজনক সেবা পাওয়ার অধিকার Right to respectful care from health service providers......................C	1	2	3	
		তথ্যসেবা পাওয়ার অধিকার<br>Right to information ....D	1	2	3	
		পরিবার পরিকল্পনার অধিকার<br>Right to family planning ....E	1	2	3	
		পারিবারিক/ স্বামী কর্তৃক সহিংসতা থেকে মুক্ত থাকার অধিকার Right to be free from domestic/intimate partner violence .... F	1	2	3	
		অন্যান্য Others......................................X	উল্লেখ করুন<br>Specify ________________________			

NO	Questions And Filters	Coding Categories	Skip
w03.	সাধারনত বিশেষ এই অধিকারগুলো আপনার এলাকার মহিলারা কতটা ভালভাবে ভোগ/আদায় করতে পারেন বা পেয়ে থাকেন?<br>In general how well are women in your area been able to enjoy/realize/receive these special rights?	খুব ভাল Very well .... 1<br>মোটামুটি ভাল Somewhat well .... 2<br>একদম ভাল নয় Not well at all... 3<br>জানি না/ মনে নাই Don't know/Can't remember.................... 9	
w04.	সাধারনত বিশেষ এই অধিকারগুলো আপনি কতটা ভালভাবে ভোগ/আদায় করতে পারেন বা পেয়ে থাকেন?<br>In general, how well have you been able to enjoy/realize/receive these special rights?	খুব ভাল Very well .... 1<br>মোটামুটি ভাল Somewhat well .... 2<br>একদম ভাল নয় Not well at all... 3<br>জানি না/ মনে নাই Don't know/Can't remember.................... 9	

NO	Questions And Filters		Unprompted Yes	Prompted Yes	No	Skip
w05.	আপনার মতে মহিলাদের বিশেষ এই অধিকারগুলো নিশ্চিত করতে কার কার ভূমিকা পালন করা উচিৎ?<br><u>[জিজ্ঞেস করুন, আরও কিছু? উত্তরদাতার নিজে থেকে দেয়া সবগুলো উত্তর প্রথমেই বৃত্তায়িত করুন। উত্তরগুলো পড়ে শুনাবেন না। একাধিক উত্তর হতে পারে। এরপর বাকি উত্তরগুলো পড়ে শুনান]</u><br>In your opinion, who do you think should play a role in assuring that women are able to realize these rights?<br><u>[Do not read out the options. Keep asking what else. Record all the answers first. Then read out the remaining options].</u>	সরকার/স্বাস্থ্য মন্ত্রনালয়<br>Government/Ministry of Health ....A	1	2	3	
		স্বামী<br>Husbands...B	1	2	3	
		পরিবারের অন্যান্য সদস্য<br>Household members...........................C	1	2	3	
		এলাকার লোকজন<br>Community ....D	1	2	3	
		অন্যান্য Others......................................X	উল্লেখ করুন<br>Specify ________________________			

NO	Questions And Filters	Coding Categories	Skip
w06.	বিশেষ এই অধিকারগুলো নিশ্চিত করতে বর্তমানে সরকার/স্বাস্থ্য মন্ত্রনালয়ের ভূমিকা কেমন বলে আপনি মনে করেন?<br>What is your opinion regarding the role currently played by the Government or MOH regarding realizing these special rights?	খুব ভাল Very well .... 1<br>মোটামুটি ভাল Somewhat well .... 2<br>একদম ভাল নয় Not well at all .... 3<br>জানি না/ মনে নাই Don't know/Can't remember 9	
w07.	বিশেষ এই অধিকারগুলো নিশ্চিত করতে বর্তমানে স্বামীদের ভূমিকা কেমন বলে আপনি মনে করেন?<br>What is your opinion regarding the role currently played by the husbands regarding realizing these special rights?	খুব ভাল Very well .... 1<br>মোটামুটি ভাল Somewhat well .... 2<br>একদম ভাল নয় Not well at all .... 3<br>জানি না/ মনে নাই Don't know/Can't remember 9	
w08.	বিশেষ এই অধিকারগুলো নিশ্চিত করতে বর্তমানে পরিবারের সদস্যদের ভূমিকা কেমন বলে আপনি মনে করেন?<br>What is your opinion regarding the role currently played by the family members regarding realizing these special rights?	খুব ভাল Very well .... 1<br>মোটামুটি ভাল Somewhat well .... 2<br>একদম ভাল নয় Not well at all .... 3<br>জানি না/ মনে নাই Don't know/Can't remember 9	
w09.	বিশেষ এই অধিকারগুলো নিশ্চিত করতে বর্তমানে এলাকার লোকজনের ভূমিকা কেমন বলে আপনি মনে করেন?<br>What is your opinion regarding the role currently played by the community regarding realizing these rights?	খুব ভাল Very well .... 1<br>মোটামুটি ভাল Somewhat well .... 2<br>একদম ভাল নয় Not well at all .... 3<br>জানি না/ মনে নাই Don't know/Can't remember 9	

NO	Questions And Filters		Unprompted Yes	Prompted Yes	No	Skip
w10.	বিশেষ এ অধিকারগুলো সম্পর্কে আপনি কিভাবে বা কোন উৎস থেকে জানতে পেরেছেন?<br>[জিজ্ঞেস করুন, আরও কিছু? উত্তরদাতার নিজে থেকে দেয়া সবগুলো উত্তর প্রথমেই বৃত্তায়িত করুন । উত্তরগুলো পড়ে শুনাবেন না। একাধিক উত্তর হতে পারে। এরপর বাকি উত্তরগুলো পড়ে শুনান]<br>How or from which source/sources have you come to know about these special rights?<br>[Do not read out the options. Keep asking what else. Record all the answers first. Then read out the remaining options]	a. বই/খবরের কাগজ Book/Newspaper .....A	1	2	3	
		b. রেডিও Radio .... B	1	2	3	
		c. টেলিভিশন Television C	1	2	3	
		d. বিলবোর্ড Billboard .... D	1	2	3	
		e. পোস্টার Poster .... E	1	2	3	
		f. লিফলেট Leaflet .... F	1	2	3	
		g. উঠান বৈঠক Courtyard meeting .... G	1	2	3	
		h. স্বাস্থ্যসেবা কেন্দ্রে মিটিং<br>Meeting in the health facility .... H	1	2	3	
		i. বাড়িতে সরকারি স্বাস্থ্যকর্মীর পরিদর্শন<br>Home visits by Govt Health Workers . I	1	2	3	
		j. বাড়িতে এনজিও স্বাস্থ্যকর্মীর পরিদর্শন<br>Home visits by NGO Health Workers . J	1	2	3	
		x. অন্যান্য Others X	উল্লেখ করুন<br>Specify ________________________			

NO	Questions And Filters	Coding Categories	Skip
w11.	বিশেষ এ অধিকারগুলো সম্পর্কে আপনি কার-কার কাছ থেকে জানতে পেরেছেন?<br>(কোড লিস্ট থেকে কোড লিখুন)<br>From whom did you come to know about these special rights?<br>(Fill out from the codebook)	ব্যক্তির কোড Person code<br>|__|__| |__|__| |__|__| |__|__| 1 2 3 4	
w12.	বিশেষ এ অধিকারগুলো সম্পর্কে আপনি কোথা থেকে জানতে পেরেছেন?<br>(কোড লিস্ট থেকে কোড লিখুন)<br>From where did you come to know about these special rights?<br>(Fill out from the codebook)	স্থানের কোড Place code<br>|__|__| |__|__| |__|__| |__|__| 1 2 3 4	
w13.	একজন দক্ষ স্বাস্থ্যকর্মীর কাছ থেকে মা ও নবজাতকের জন্য নিয়মিত স্বাস্থ্যসেবা গ্রহণ করার গুরুত্ব কতটুকু বলে আপনি মনে করেন?<br>In your opinion, how important it is to seek care from trained providers for routine maternal and newborn services?	খুব গুরুত্বপূর্ন Very important .... 1<br>মোটামুটি গুরুত্বপূর্ন Somewhat important .... 2<br>একদম গুরুত্বপূর্ন নয় Not important at all .... 3<br>জানি না/ মনে নাই Don't know/Can't remember 9	
w14.	জরুরী অবস্থায় একজন দক্ষ স্বাস্থ্যকর্মীর কাছ থেকে মা ও নবজাতকের জন্য স্বাস্থ্যসেবা গ্রহণ করার গুরুত্ব কতটুকু বলে আপনি মনে করেন?<br>In your opinion, how important it is to seek care from trained providers for emergency maternal and newborn services?	খুব গুরুত্বপূর্ন Very important .... 1<br>মোটামুটি গুরুত্বপূর্ন Somewhat important .... 2<br>একদম গুরুত্বপূর্ন নয় Not important at all .... 3<br>জানি না/ মনে নাই Don't know/Can't remember 9	
w15.	গর্ভকালীন সময়ে একজন মহিলার মেডিকেল চেকআপ (ANC) করা দরকার কি?<br>Do you think a pregnant woman should receive routine medical check-up (ANC) during pregnancy?	হ্যাঁ Yes .... 1<br>না No .... 2<br>জানি না/ মনে নাই Don't know/Can't remember 9	**2/9 → w21**
w16.	গর্ভকালীন সময়ে একজন মহিলার কমপক্ষে কত বার মেডিকেল চেকআপ (ANC) করা দরকার?<br>How many times a pregnant woman should receive routine medical check-up (ANC) during pregnancy?	বার (গর্ভকালীন সময়ে) .... |__|__| Times (during pregnancy)<br>জানি না/ মনে নাই Don't know/Can't remember 99	

NO	Questions And Filters	Coding Categories	Skip
w17.	গর্ভকালীন সময়ে প্রথম কখন এরকম মেডিকেল চেক-আপ (ANC) করা দরকার বলে আপনি মনে করেন?<br>When do you think the first ANC check up should take place?	প্রথম ৩ মাসে 1st trimester (1-3 months) .... 1<br>দ্বিতীয় ৩ মাসে 2nd trimester (4-6 months) .... 2<br>তৃতীয় ৩ মাসে 3rd trimester (7-9 months)............................. 3<br>জানি না/ মনে নাই Don't know/Can't remember.................... 9	

w18.	গর্ভকালীন সময়ে মেডিকেল চেক-আপ (ANC) করা দরকার- এ বিষয়ে আপনি কিভাবে বা কোন উৎস থেকে জানতে পেরেছেন?<br>[উত্তরগুলো পড়ে শুনাবেন না। জিজ্ঞেস করুন, আরও কিছু? উত্তরদাতার নিজে থেকে দেয়া সবগুলো উত্তর প্রথমেই বৃত্তায়িত করুন। এরপর বাকি উত্তরগুলো পড়ে শুনান]<br>How or from which source/sources you have come to know about the importance of receiving ANC during the pregnancy<br>[Do not read out the options. Keep asking what else. Record all the answers first. Then read out the remaining options]	Unprompted Yes	Prompted Yes	No
	a. বই/খবরের কাগজ Book/Newspaper .....A	1	2	3
	b. রেডিও Radio ....B	1	2	3
	c. টেলিভিশন Television C	1	2	3
	d. বিলবোর্ড Billboard ....D	1	2	3
	e. পোস্টার Poster ....E	1	2	3
	f. লিফলেট Leaflet.................................. F	1	2	3
	g. উঠান বৈঠক Courtyard meeting ....G	1	2	3
	h. এলাকায় স্বাস্থ্য বিষয়ক মিটিং Health related meeting in community ....H	1	2	3
	i. বাড়িতে সরকারি স্বাস্থ্যকর্মীর পরিদর্শন Home visits by Govt Health Workers I	1	2	3
	j. বাড়িতে এনজিও স্বাস্থ্যকর্মীর পরিদর্শন Home visits by NGO Health Workers.....................J	1	2	3
	x. অন্যান্য Others X	উল্লেখ করুন<br>Specify ________________________		

NO	Questions And Filters	Coding Categories	Skip
w19.	গর্ভকালীন সময়ে মেডিকেল চেক-আপ (ANC) করা দরকার- এ বিষয়ে আপনি কার কার কাছ থেকে জানতে পেরেছেন? (কোড লিস্ট থেকে কোড লিখুন)<br>From whom did you come to know about the importance of receiving ANC during pregnancy? (Fill out from the codebook)	ব্যক্তির কোড Person code<br>|___|___| |___|___| |___|___| |___|___| 1 2 3 4	
w20.	গর্ভকালীন সময়ে মেডিকেল চেক-আপ (ANC) করা দরকার- এ বিষয়ে আপনি কোথা থেকে জানতে পেরেছেন? (কোড লিস্ট থেকে কোড লিখুন)<br>From where did you come to know about the importance of receiving ANC during pregnancy? (Fill out from the codebook)	স্থানের কোড Place code<br>|___|___| |___|___| |___|___| |___|___| 1 2 3 4	
w21.	শেষ গর্ভের সময় বাড়িতে, স্বাস্থ্যকেন্দ্রে বা অন্য কোন স্থানে কোন স্বাস্থ্যকর্মীর দ্বারা আপনি গর্ভকালীন মেডিকেল চেক-আপ (ANC) করেছিলেন কি? (শুধু মাত্র নিয়মিত চেকাপের জন্য)<br>Did you see (consult) any health care provider in home, health facility or other place for ANC during your most recent pregnancy? [for routine check-up only]	হ্যাঁ Yes.. 1<br>→কতবার How many times: |____| বার times<br>না No... 2<br>জানি না/ মনে নাই Don't know/Can't remember.................... 9	1 →<br>w 23
w22.	শেষ গর্ভের সময়, গর্ভকালীন মেডিকেল চেক-আপের (ANC) জন্য আপনি কাউকে না দেখিয়ে থাকলে তার কারনগুলো কি কি?<br>[উত্তরগুলো পড়ে শুনাবেন না। জিজ্ঞেস করুন, আরও কিছু? উত্তরদাতার নিজে থেকে দেয়া সবগুলো উত্তর প্রথমেই বৃত্তায়িত করুন। এরপর বাকি উত্তরগুলো পড়ে]<br>If not, why didn't you see (consult) anyone for ANC during your most recent pregnancy?<br>[Do not read out the options. Keep asking what else. Record all the answers first. Then read out the remaining options]	a. প্রয়োজন মনে হয়নি Didn't consider it to be important........A<br>b. অর্থের সমস্যা Lack of money...B<br>c. সাথে যাওয়ার কেউ ছিলনা No one to accompany ....C<br>d. স্বামীর অনুমতি ছিলনা Husband did not permit ....D<br>e. পরিবারের অন্য সদস্যদের অসম্মতি ছিল<br>Other family members did not permit ....E<br>f. রাস্তা খারাপ Road condition was bad .... F<br>g. স্বাস্থ্য সেবাপ্রদানকারীর সেবার মান নিয়ে সন্তুষ্ট নই<br>Not happy with service provider's service ....G<br>x.অন্যান্য, নির্দিষ্ট করুন Others..X<br>(Specify) ________________________	
w23.	শেষ গর্ভের সময়, আপনাকে দেখতে বা আপনার স্বাস্থ্যের ব্যাপারে উপদেশ দিতে আপনার বাসায় কেউ এসেছিলেন কি?<br>Did anybody visit you to discuss issues regarding your health during your most recent pregnancy?	হ্যাঁ Yes .... 1<br>না No... 2<br>জানি না/ মনে নাই Don't know/Can't remember.................... 9	2/9<br>→<br>w25
w24.	শেষ গর্ভের সময়, আপনাকে দেখতে বা আপনার স্বাস্থ্যের ব্যাপারে উপদেশ দিতে কে কে আপনার বাসায় এসেছিলেন?<br>[জিজ্ঞেস করুন, আরও কিছু? উত্তরদাতার নিজে থেকে দেয়া সবগুলো উত্তরই বৃত্তায়িত করুন । উত্তরগুলো পড়ে শুনাবেন না। একাধিক উত্তর হতে পারে।]<br>Who had visited you to discuss issues regarding your health during your most recent pregnancy?<br>[Do not read out the options. Keep asking what else. Record all the answers.]	ব্যক্তির কোড Person Code:<br>|___|___| |___|___| |___|___| |___|___| 1 2 3 4	

NO	Questions And Filters			Coding Categories		Skip
	**w21 থেকে পূরুন করুন**	**1st Visit**	**2ndVisit**	**3rd Visit**	**4th Visit**	**→**
w25.	দয়া করে আমাকে আপনার গর্ভকালীন মেডিকেল চেক-আপ (ANC) বিষয়ে নিম্নলিখিত প্রশ্নগুলোর উত্তর দিবেন:<br>শেষ গর্ভের সময়, আপনি কি গর্ভকালীন মেডিকেল চেক-আপ (ANC) নিয়েছিলেন/করিয়েছিলেন?<br>Please provide me the information regarding your ANC visits: Did you receive ANC?	হ্যাঁ Yes = 1<br>না No = 2<br>জানি না/<br>মনে নাই<br>DK/DR = 9	হ্যাঁ Yes = 1<br>না No = 2<br>জানি না/<br>মনে নাই<br>DK/DR = 9	হ্যাঁ Yes = 1<br>না No = 2<br>জানি না/<br>মনে নাই<br>DK/DR = 9	হ্যাঁ Yes = 1<br>না No = 2<br>জানি না/<br>মনে নাই<br>DK/DR = 9	**2/9 → w58**
w26.	শেষ গর্ভের সময়, গর্ভকালীন মেডিকেল চেক-আপের (ANC) জন্য আপনি কাকে দেখিয়েছিলেন?<br>(কোড লিস্ট থেকে কোড লিখুন)<br>From whom did you seek antenatal care during your most recent pregnancy?<br>(Fill out from the codebook)	ব্যাক্তির কোড<br>Person code:<br>|____||____|	ব্যাক্তির কোড<br>Person code:<br>|____||____|	ব্যাক্তির কোড<br>Person code:<br>|____||____|	ব্যাক্তির কোড<br>Person code:<br>|____||____|	
w27.	শেষ গর্ভের সময়, গর্ভকালীন মেডিকেল চেক-আপ (ANC) আপনি কোথা থেকে নিয়েছিলেন/করিয়েছিলেন?<br>(কোড লিস্ট থেকে কোড লিখুন)<br>From where did you seek antenatal care during your most recent pregnancy?<br>(Fill out from the codebook)	স্থানের কোড<br>Place code:<br>|____|____|	স্থানের কোড<br>Place code:<br>|____|____|	স্থানের কোড<br>Place code:<br>|____|____|	স্থানের কোড<br>Place code:<br>|____|____|	
w28.	শেষ গর্ভের সময়, গর্ভকালীন মেডিকেল চেক-আপ (ANC) যখন করানো হয়েছিল, তখন আপনি কত মাসের গর্ভবতী ছিলেন?<br>(কোড লিস্ট থেকে কোড লিখুন)<br>How many months pregnant were you when you received ANC visit?<br>(Fill out from the codebook provided)	|____| মাসের গর্ভবতী<br>Months pregnant<br>জানি না/<br>মনে নাই<br>DK/DR=9	|____| মাসের গর্ভবতী<br>Months pregnant<br>জানি না/<br>মনে নাই<br>DK/DR=9	|____| মাসের গর্ভবতী<br>Months pregnant<br>জানি না/<br>মনে নাই<br>DK/DR=9	|____| মাসের গর্ভবতী<br>Months pregnant<br>জানি না/<br>মনে নাই<br>DK/DR=9	
w29.	গর্ভকালীন চেক আপের (ANC) সময় অন্যান্যরা (স্বাস্থ্যসেবা প্রদানকারী এবং আপনি যাদেরকে উপস্থিত থাকার অনুমতি দিয়েছিলেন তারা ব্যতিত অন্য কেউ) আপনাকে দেখতে পাচ্ছিল কি?<br>Were others (other than the healthcare provider and those you invited to be present during the contact) able to see you during your ANC contact?	হ্যাঁ Yes = 1<br>না No = 2<br>জানি না/<br>মনে নাই<br>DK/DR = 9	হ্যাঁ Yes = 1<br>না No = 2<br>জানি না/<br>মনে নাই<br>DK/DR = 9	হ্যাঁ Yes = 1<br>না No = 2<br>জানি না/<br>মনে নাই<br>DK/DR = 9	হ্যাঁ Yes = 1<br>না No = 2<br>জানি না/<br>মনে নাই<br>DK/DR = 9	
w30.	গর্ভকালীন চেক আপের (ANC) সময় অন্যান্যরা (স্বাস্থ্যসেবা প্রদানকারী এবং আপনি যাদেরকে উপস্থিত থাকার অনুমতি দিয়েছিলেন তারা ব্যতিত অন্য কেউ) আপনার কথা শুনতে পাচ্ছিল কি?<br>Were others able to hear (other than the healthcare provider and those you invited to be present during the contact) you during your ANC contact?	হ্যাঁ Yes = 1<br>না No = 2<br>জানি না/<br>মনে নাই<br>DK/DR = 9	হ্যাঁ Yes = 1<br>না No = 2<br>জানি না/<br>মনে নাই<br>DK/DR = 9	হ্যাঁ Yes = 1<br>না No = 2<br>জানি না/<br>মনে নাই<br>DK/DR = 9	হ্যাঁ Yes = 1<br>না No = 2<br>জানি না/<br>মনে নাই<br>DK/DR = 9	

NO	Questions And Filters					Skip
		Coding Categories				
		**1st Visit**	**2ndVisit**	**3rd Visit**	**4th Visit**	
w31.	গর্ভকালীন চেক আপের (ANC) সময় স্বাস্থ্যসেবা প্রদানকারী কি কোন ছবিওয়ালা কার্ড/ফ্লিপ চার্ট দেখিয়ে ব্যাখ্যা করেছিল?<br>Did the healthcare provider use any visual tools when explaining things to you during the ANC contact?	হ্যাঁ Yes = 1<br>না No = 2<br>জানি না/<br>মনে নাই<br>DK/DR = 9	হ্যাঁ Yes = 1<br>না No = 2<br>জানি না/<br>মনে নাই<br>DK/DR = 9	হ্যাঁ Yes = 1<br>না No = 2<br>জানি না/<br>মনে নাই<br>DK/DR = 9	হ্যাঁ Yes = 1<br>না No = 2<br>জানি না/<br>মনে নাই<br>DK/DR = 9	
w32.	গর্ভকালীন মেডিকেল চেক-আপের (ANC) সময় আপনাকে গর্ভকালীন, প্রসবকালীন এবং প্রসবপরবর্তী বিপদচিহ্ন সম্পর্কে কিছু বলা হয়েছিল কি?<br>During your ANC contact, were you told about the danger signs of pregnancy, childbirth and afterbirth?	হ্যাঁ Yes = 1<br>না No = 2<br>জানি না/<br>মনে নাই<br>DK/DR = 9	হ্যাঁ Yes = 1<br>না No = 2<br>জানি না/<br>মনে নাই<br>DK/DR = 9	হ্যাঁ Yes = 1<br>না No = 2<br>জানি না/<br>মনে নাই<br>DK/DR = 9	হ্যাঁ Yes = 1<br>না No = 2<br>জানি না/<br>মনে নাই<br>DK/DR = 9	
w33.	গর্ভকালীন চেকআপের (ANC) সময় গর্ভকালীন, প্রসবকালীন এবং প্রসবপরবর্তী বিপদচিহ্ন/ জটিলতাগুলো দেখা দিলে কোথায় যেতে হবে সে ব্যাপারে আপনাকে কিছু বলা হয়েছিল কি?<br>Were you told where to go if you had any of these pregnancy, childbirth and after birth related complications?	হ্যাঁ Yes = 1<br>না No = 2<br>জানি না/<br>মনে নাই<br>DK/DR = 9	হ্যাঁ Yes = 1<br>না No = 2<br>জানি না/<br>মনে নাই<br>DK/DR = 9	হ্যাঁ Yes = 1<br>না No = 2<br>জানি না/<br>মনে নাই<br>DK/DR = 9	হ্যাঁ Yes = 1<br>না No = 2<br>জানি না/<br>মনে নাই<br>DK/DR = 9	
w34.	গর্ভকালীন মেডিকেল চেকআপের (ANC) সময় আপনাকে নবজাতকের বিপদচিহ্ন সম্পর্কে কিছু বলা হয়েছিল কি?<br>During your ANC contact, were you told about the danger signs of newborns?	হ্যাঁ Yes = 1<br>না No = 2<br>জানি না/<br>মনে নাই<br>DK/DR = 9	হ্যাঁ Yes = 1<br>না No = 2<br>জানি না/<br>মনে নাই<br>DK/DR = 9	হ্যাঁ Yes = 1<br>না No = 2<br>জানি না/<br>মনে নাই<br>DK/DR = 9	হ্যাঁ Yes = 1<br>না No = 2<br>জানি না/<br>মনে নাই<br>DK/DR = 9	**2/9 → w36**
w35.	নবজাতকের এই বিপদচিহ্ন/ জটিলতাগুলো দেখা দিলে কোথায় যেতে হবে সেই ব্যাপারে আপনাকে কিছু বলা হয়েছিল কি?<br>Were you told where to go if the newborn had any of these newborn related complications?	হ্যাঁ Yes = 1<br>না No = 2<br>জানি না/<br>মনে নাই<br>DK/DR = 9	হ্যাঁ Yes = 1<br>না No = 2<br>জানি না/<br>মনে নাই<br>DK/DR = 9	হ্যাঁ Yes = 1<br>না No = 2<br>জানি না/<br>মনে নাই<br>DK/DR = 9	হ্যাঁ Yes = 1<br>না No = 2<br>জানি না/<br>মনে নাই<br>DK/DR = 9	
w36.	w36. a.<br>গর্ভকালীন মেডিকেল চেকআপের (ANC) সময় আপনার স্বামী আপনার সাথে গিয়েছিল কি?<br>Did your husband travel with you during the ANC contact?	হ্যাঁ Yes = 1<br>না No = 2<br>জানি না/<br>মনে নাই<br>DK/DR = 9	হ্যাঁ Yes = 1<br>না No = 2<br>জানি না/<br>মনে নাই<br>DK/DR = 9	হ্যাঁ Yes = 1<br>না No = 2<br>জানি না/<br>মনে নাই<br>DK/DR = 9	হ্যাঁ Yes = 1<br>না No = 2<br>জানি না/<br>মনে নাই<br>DK/DR = 9	
	w36. b.<br>গর্ভকালীন মেডিকেল চেকআপের (ANC) সময় আপনার স্বামী আপনার সাথে উপস্থিত ছিল কি?<br>Was your husband present with you during the ANC contact?	হ্যাঁ Yes = 1<br>না No = 2<br>জানি না/<br>মনে নাই<br>DK/DR = 9	হ্যাঁ Yes = 1<br>না No = 2<br>জানি না/<br>মনে নাই<br>DK/DR = 9	হ্যাঁ Yes = 1<br>না No = 2<br>জানি না/<br>মনে নাই<br>DK/DR = 9	হ্যাঁ Yes = 1<br>না No = 2<br>জানি না/<br>মনে নাই<br>DK/DR = 9	
w37.	আপনি কি গর্ভকালীন মেডিকেল চেকআপের (ANC) সময় আপনার স্বামীকে আপনার সাথে উপস্থিত রাখতে চেয়েছিলেন?<br>Did you want your husband to be present with you during the ANC contact?	হ্যাঁ Yes = 1<br>না No = 2<br>জানি না/<br>মনে নাই<br>DK/DR = 9	হ্যাঁ Yes = 1<br>না No = 2<br>জানি না/<br>মনে নাই<br>DK/DR = 9	হ্যাঁ Yes = 1<br>না No = 2<br>জানি না/<br>মনে নাই<br>DK/DR = 9	হ্যাঁ Yes = 1<br>না No = 2<br>জানি না/<br>মনে নাই<br>DK/DR = 9	
w38.	গর্ভকালীন মেডিকেল চেকআপের (ANC) সময় আপনার স্বামীকে আপনি উপস্থিত রাখতে চান কিনা সেই ব্যাপারে স্বাস্থ্যসেবা প্রদানকারী আপনাকে জিজ্ঞেস করেছিলেন কি?<br>Did the healthcare provider ask you whether you wanted your husband to be present during your ANC contact?	হ্যাঁ Yes = 1<br>না No = 2<br>জানি না/<br>মনে নাই<br>DK/DR = 9	হ্যাঁ Yes = 1<br>না No = 2<br>জানি না/<br>মনে নাই<br>DK/DR = 9	হ্যাঁ Yes = 1<br>না No = 2<br>জানি না/<br>মনে নাই<br>DK/DR = 9	হ্যাঁ Yes = 1<br>না No = 2<br>জানি না/<br>মনে নাই<br>DK/DR = 9	

NO	Questions And Filters	Coding Categories				Skip
		1st Visit	2ndVisit	3rd Visit	4th Visit	
w39.	গর্ভকালীন মেডিকেল চেকআপের (ANC) সময় পরিবারের অন্যান্য সদস্যরা (স্বামী ছাড়া) আপনার সাথে উপস্থিত ছিল কি?<br>Were any other family members (Except husband) present with you during the ANC contact?	হ্যাঁ Yes = 1<br>না No = 2<br>জানি না/<br>মনে নাই<br>DK/DR = 9	হ্যাঁ Yes = 1<br>না No = 2<br>জানি না/<br>মনে নাই<br>DK/DR = 9	হ্যাঁ Yes = 1<br>না No = 2<br>জানি না/<br>মনে নাই<br>DK/DR = 9	হ্যাঁ Yes = 1<br>না No = 2<br>জানি না/<br>মনে নাই<br>DK/DR = 9	
w40.	গর্ভকালীন মেডিকেল চেকআপের (ANC) সময় অন্যান্য আত্মীয়/প্রতিবেশী/বন্ধু (স্বামী এবং পরিবারের অন্য সদস্য ছাড়া) আপনার সাথে উপস্থিত ছিল কি?<br>Were any other relative/ neighbor/friend (Except husband and other family member) present with you during the ANC contact?	হ্যাঁ Yes = 1<br>না No = 2<br>জানি না/<br>মনে নাই<br>DK/DR = 9	হ্যাঁ Yes = 1<br>না No = 2<br>জানি না/<br>মনে নাই<br>DK/DR = 9	হ্যাঁ Yes = 1<br>না No = 2<br>জানি না/<br>মনে নাই<br>DK/DR = 9	হ্যাঁ Yes = 1<br>না No = 2<br>জানি না/<br>মনে নাই<br>DK/DR = 9	
w41.	আপনার গর্ভকালীন মেডিকেল চেকআপের (ANC) সময় স্বাস্থ্যসেবাপ্রদানকারী আপনাকে কীভাবে অভ্যর্থনা জানিয়েছিল?<br>How well did the healthcare provider greet you during the ANC contact?	খুব ভাল<br>Very well =1<br>মোটামুটি ভাল<br>Somewhat well = 2<br>একদম ভাল নয়<br>Not well at all =3<br>জানি না/<br>মনে নাই<br>DK/DR = 9	খুব ভাল<br>Very well =1<br>মোটামুটি ভাল<br>Somewhat well = 2<br>একদম ভাল নয়<br>Not well at all =3<br>জানি না/<br>মনে নাই<br>DK/DR = 9	খুব ভাল<br>Very well =1<br>মোটামুটি ভাল<br>Somewhat well = 2<br>একদম ভাল নয়<br>Not well at all =3<br>জানি না/<br>মনে নাই<br>DK/DR = 9	খুব ভাল<br>Very well =1<br>মোটামুটি ভাল<br>Somewhat well = 2<br>একদম ভাল নয়<br>Not well at all =3<br>জানি না/<br>মনে নাই<br>DK/DR = 9	
w42.	স্বাস্থ্যসেবাপ্রদানকারী আপনার সমস্যাগুলো শুনতে/ বুঝতে কতটুকু চেষ্টা করেছিল?<br>How well did the healthcare provider try to listen to your problems?	খুব ভাল<br>Very well =1<br>মোটামুটি ভাল<br>Somewhat well = 2<br>একদম ভাল নয়<br>Not well at all =3<br>জানি না/<br>মনে নাই<br>DK/DR = 9	খুব ভাল<br>Very well =1<br>মোটামুটি ভাল<br>Somewhat well = 2<br>একদম ভাল নয়<br>Not well at all =3<br>জানি না/<br>মনে নাই<br>DK/DR = 9	খুব ভাল<br>Very well =1<br>মোটামুটি ভাল<br>Somewhat well = 2<br>একদম ভাল নয়<br>Not well at all =3<br>জানি না/<br>মনে নাই<br>DK/DR = 9	খুব ভাল<br>Very well =1<br>মোটামুটি ভাল<br>Somewhat well = 2<br>একদম ভাল নয়<br>Not well at all =3<br>জানি না/<br>মনে নাই<br>DK/DR = 9	
w43.	স্বাস্থ্যসেবাপ্রদানকারী গর্ভকালীন, প্রসবকালীন এবং প্রসবপরবর্তী সময়ে আপনার এবং নবজাতকের স্বাস্থ্য এর ব্যাপারে আপনি কতটুকু জানেন তা বোঝার ব্যপারে কতটুকু চেষ্টা করেছিল?<br>How well did the healthcare provider try to assess what you already knew about pregnancy, childbirth and about taking care of your and your baby after birth?	খুব ভাল<br>Very well =1<br>মোটামুটি ভাল<br>Somewhat well = 2<br>একদম ভাল নয়<br>Not well at all =3<br>জানি না/<br>মনে নাই<br>DK/DR = 9	খুব ভাল<br>Very well =1<br>মোটামুটি ভাল<br>Somewhat well = 2<br>একদম ভাল নয়<br>Not well at all =3<br>জানি না/<br>মনে নাই<br>DK/DR = 9	খুব ভাল<br>Very well =1<br>মোটামুটি ভাল<br>Somewhat well = 2<br>একদম ভাল নয়<br>Not well at all =3<br>জানি না/<br>মনে নাই<br>DK/DR = 9	খুব ভাল<br>Very well =1<br>মোটামুটি ভাল<br>Somewhat well = 2<br>একদম ভাল নয়<br>Not well at all =3<br>জানি না/<br>মনে নাই<br>DK/DR = 9	
w44.	স্বাস্থ্যসেবাপ্রদানকারী আপনার সামগ্রিক অবস্থা (পরিবার, পারিবারিক জীবন, যেসব সমস্যার সম্মুখীন হতে পারেন ইত্যাদি) বোঝার ব্যপারে কতটুকু চেষ্টা করেছিল?<br>How well did the healthcare provider try to assess your overall situation (family, family life, problems you may be facing etc.)?	খুব ভাল<br>Very well =1<br>মোটামুটি ভাল<br>Somewhat well = 2<br>একদম ভাল নয়<br>Not well at all =3<br>জানি না/<br>মনে নাই<br>DK/DR = 9	খুব ভাল<br>Very well =1<br>মোটামুটি ভাল<br>Somewhat well = 2<br>একদম ভাল নয়<br>Not well at all =3<br>জানি না/<br>মনে নাই<br>DK/DR = 9	খুব ভাল<br>Very well =1<br>মোটামুটি ভাল<br>Somewhat well = 2<br>একদম ভাল নয়<br>Not well at all =3<br>জানি না/<br>মনে নাই<br>DK/DR = 9	খুব ভাল<br>Very well =1<br>মোটামুটি ভাল<br>Somewhat well = 2<br>একদম ভাল নয়<br>Not well at all =3<br>জানি না/<br>মনে নাই<br>DK/DR = 9	

NO	Questions And Filters	Coding Categories				Skip
		1st Visit	2ndVisit	3rd Visit	4th Visit	
w45.	আপনার সমস্যাগুলোর সমাধান খুঁজে বের করার ব্যাপারে স্বাস্থ্যসেবাপ্রদানকারী কতটুকু চেষ্টা করেছিল?<br>How well did the healthcare provider try to help you to find solutions for your problems?	খুব ভাল<br>Very well =1<br>মোটামুটি ভাল<br>Somewhat well = 2<br>একদম ভাল নয়<br>Not well at all =3<br>জানি না/ মনে নাই<br>DK/DR = 9	খুব ভাল<br>Very well =1<br>মোটামুটি ভাল<br>Somewhat well = 2<br>একদম ভাল নয়<br>Not well at all =3<br>জানি না/ মনে নাই<br>DK/DR = 9	খুব ভাল<br>Very well =1<br>মোটামুটি ভাল<br>Somewhat well = 2<br>একদম ভাল নয়<br>Not well at all =3<br>জানি না/ মনে নাই<br>DK/DR = 9	খুব ভাল<br>Very well =1<br>মোটামুটি ভাল<br>Somewhat well = 2<br>একদম ভাল নয়<br>Not well at all =3<br>জানি না/ মনে নাই<br>DK/DR = 9	
w46.	আলোচনায় অংশগ্রহন করা এবং প্রশ্ন করার ব্যপারে স্বাস্থ্যসেবাপ্রদানকারী আপনাকে কতটুকু উৎসাহিত করেছিল?<br>How well did the healthcare provider encourage you to take part in the discussion or encourage you to speak during the discussion?	খুব ভাল<br>Very well =1<br>মোটামুটি ভাল<br>Somewhat well = 2<br>একদম ভাল নয়<br>Not well at all =3<br>জানি না/ মনে নাই<br>DK/DR = 9	খুব ভাল<br>Very well =1<br>মোটামুটি ভাল<br>Somewhat well = 2<br>একদম ভাল নয়<br>Not well at all =3<br>জানি না/ মনে নাই<br>DK/DR = 9	খুব ভাল<br>Very well =1<br>মোটামুটি ভাল<br>Somewhat well = 2<br>একদম ভাল নয়<br>Not well at all =3<br>জানি না/ মনে নাই<br>DK/DR = 9	খুব ভাল<br>Very well =1<br>মোটামুটি ভাল<br>Somewhat well = 2<br>একদম ভাল নয়<br>Not well at all =3<br>জানি না/ মনে নাই<br>DK/DR = 9	
w47.	স্বাস্থ্যসেবাপ্রদানকারীর দেওয়া তথ্যগুলো আপনি কতটুকু বুঝতে পেরেছিলেন?<br>How well did you understand the information provided by the health worker?	খুব ভাল<br>Very well =1<br>মোটামুটি ভাল<br>Somewhat well = 2<br>একদম ভাল নয়<br>Not well at all =3<br>জানি না/ মনে নাই<br>DK/DR = 9	খুব ভাল<br>Very well =1<br>মোটামুটি ভাল<br>Somewhat well = 2<br>একদম ভাল নয়<br>Not well at all =3<br>জানি না/ মনে নাই<br>DK/DR = 9	খুব ভাল<br>Very well =1<br>মোটামুটি ভাল<br>Somewhat well = 2<br>একদম ভাল নয়<br>Not well at all =3<br>জানি না/ মনে নাই<br>DK/DR = 9	খুব ভাল<br>Very well =1<br>মোটামুটি ভাল<br>Somewhat well = 2<br>একদম ভাল নয়<br>Not well at all =3<br>জানি না/ মনে নাই<br>DK/DR = 9	
w48.	স্বাস্থ্যসেবাপ্রদানকারী আপনার শারিরীক পরীক্ষা করার আগে আপনার অনুমতি নিয়েছিল কি?<br>Did the health care provider ask your permission before carrying out physical examinations?	হ্যাঁ Yes = 1<br>না No = 2<br>জানি না/ মনে নাই<br>DK/DR = 9	হ্যাঁ Yes = 1<br>না No = 2<br>জানি না/ মনে নাই<br>DK/DR = 9	হ্যাঁ Yes = 1<br>না No = 2<br>জানি না/ মনে নাই<br>DK/DR = 9	হ্যাঁ Yes = 1<br>না No = 2<br>জানি না/ মনে নাই<br>DK/DR = 9	
w49.	স্বাস্থ্যসেবাপ্রদানকারী আপনার শারিরীক পরীক্ষা করার আগে কি করতে যাচ্ছে- তা আপনাকে বুঝিয়ে বলেছিল?<br>Did the healthcare provider explain to you what she was going to do before conducting physical examinations?	হ্যাঁ Yes = 1<br>না No = 2<br>জানি না/ মনে নাই<br>DK/DR = 9	হ্যাঁ Yes = 1<br>না No = 2<br>জানি না/ মনে নাই<br>DK/DR = 9	হ্যাঁ Yes = 1<br>না No = 2<br>জানি না/ মনে নাই<br>DK/DR = 9	হ্যাঁ Yes = 1<br>না No = 2<br>জানি না/ মনে নাই<br>DK/DR = 9	
w50.	গর্ভকালীন মেডিকেল চেকআপের (ANC) সময় আপনার সাথে এমন কোন কিছু/আচরন করা হয়েছে যেটিতে আপনার গোপনীয়তা ভঙ্গ হয়েছে হয়েছে বলে মনে করেন কি?<br>At any point during the ANC were you treated in a way that violated your privacy?	হ্যাঁ Yes = 1<br>না No = 2<br>জানি না/ মনে নাই<br>DK/DR = 9	হ্যাঁ Yes = 1<br>না No = 2<br>জানি না/ মনে নাই<br>DK/DR = 9	হ্যাঁ Yes = 1<br>না No = 2<br>জানি না/ মনে নাই<br>DK/DR = 9	হ্যাঁ Yes = 1<br>না No = 2<br>জানি না/ মনে নাই<br>DK/DR = 9	

NO	Questions And Filters	Coding Categories				Skip
		**1st Visit**	**2nd Visit**	**3rd Visit**	**4th Visit**	
w51.	এই মেডিকেল চেকআপের (ANC) সময় স্বাস্থ্যসেবাপ্রদানকারী এমন কোন শব্দ বা আচরন করেছিল যা আপনাকে অস্বস্তিতে ফেলেছিল অথবা এমন কোন আচরন করেছিল যাতে আপনি অপমানিত বা অসম্মানিত বোধ করেছিলেন?<br>At any point during your ANC contact did the healthcare provider talk or use a tone or facial expression that made feel uncomfortable or any other kind of behavior that made you feel humiliated or disrespected?	হ্যাঁ Yes = 1<br>না No = 2<br>জানি না/<br>মনে নাই<br>DK/DR = 9	হ্যাঁ Yes = 1<br>না No = 2<br>জানি না/<br>মনে নাই<br>DK/DR = 9	হ্যাঁ Yes = 1<br>না No = 2<br>জানি না/<br>মনে নাই<br>DK/DR = 9	হ্যাঁ Yes = 1<br>না No = 2<br>জানি না/<br>মনে নাই<br>DK/DR = 9	**2/9 → w58**
	আপনার মতে, স্বাস্থ্যসেবাপ্রদানকারী কেন আপনার সাথে এমন আচরন করেছিল? নিম্নোক্ত প্রশ্নগুলো পড়ুন<br>In your opinion, why did the healthcare provider treat you like this? [Read out the followings]					
w52.	আপনার বয়স<br>Age	হ্যাঁ Yes = 1<br>না No = 2<br>জানি না/<br>মনে নাই<br>DK/DR = 9	হ্যাঁ Yes = 1<br>না No = 2<br>জানি না/<br>মনে নাই<br>DK/DR = 9	হ্যাঁ Yes = 1<br>না No = 2<br>জানি না/<br>মনে নাই<br>DK/DR = 9	হ্যাঁ Yes = 1<br>না No = 2<br>জানি না/<br>মনে নাই<br>DK/DR = 9	
w53.	আপনার লিঙ্গ<br>Sex	হ্যাঁ Yes = 1<br>না No = 2<br>জানি না/<br>মনে নাই<br>DK/DR = 9	হ্যাঁ Yes = 1<br>না No = 2<br>জানি না/<br>মনে নাই<br>DK/DR = 9	হ্যাঁ Yes = 1<br>না No = 2<br>জানি না/<br>মনে নাই<br>DK/DR = 9	হ্যাঁ Yes = 1<br>না No = 2<br>জানি না/<br>মনে নাই<br>DK/DR = 9	
w54.	আপনার ধর্ম<br>Religion	হ্যাঁ Yes = 1<br>না No = 2<br>জানি না/<br>মনে নাই<br>DK/DR = 9	হ্যাঁ Yes = 1<br>না No = 2<br>জানি না/<br>মনে নাই<br>DK/DR = 9	হ্যাঁ Yes = 1<br>না No = 2<br>জানি না/<br>মনে নাই<br>DK/DR = 9	হ্যাঁ Yes = 1<br>না No = 2<br>জানি না/<br>মনে নাই<br>DK/DR = 9	
w55.	আপনার আর্থসামাজিক অবস্থান<br>Socio-economic status	হ্যাঁ Yes = 1<br>না No = 2<br>জানি না/<br>মনে নাই<br>DK/DR = 9	হ্যাঁ Yes = 1<br>না No = 2<br>জানি না/<br>মনে নাই<br>DK/DR = 9	হ্যাঁ Yes = 1<br>না No = 2<br>জানি না/<br>মনে নাই<br>DK/DR = 9	হ্যাঁ Yes = 1<br>না No = 2<br>জানি না/<br>মনে নাই<br>DK/DR = 9	
w56.	শিক্ষাগত যোগ্যতা<br>Educational status	হ্যাঁ Yes = 1<br>না No = 2<br>জানি না/<br>মনে নাই<br>DK/DR = 9	হ্যাঁ Yes = 1<br>না No = 2<br>জানি না/<br>মনে নাই<br>DK/DR = 9	হ্যাঁ Yes = 1<br>না No = 2<br>জানি না/<br>মনে নাই<br>DK/DR = 9	হ্যাঁ Yes = 1<br>না No = 2<br>জানি না/<br>মনে নাই<br>DK/DR = 9	
w57.	অন্যান্য, নির্দিষ্ট করুন<br>Others, specify					

NO	Question	Item	Unprompted Yes	Prompted Yes	No	Skip
w58.	গর্ভকালীন ও প্রসবকালীন সময়ে কোন কোন ক্ষেত্রে আপনি আপনার স্বামীর অংশগ্রহণ প্রত্যাশা করেন?<br>[উত্তরগুলো পড়ে শুনাবেন না। জিজ্ঞেস করুন, আরও কিছু? উত্তরদাতার নিজে থেকে দেয়া সবগুলো উত্তর প্রথমেই বৃত্তায়িত করুন। এরপর বাকি উত্তরগুলো পড়ে শুনান]<br>In which aspect/s of your pregnancy, childbirth and after birth do you want your husband to be involved?<br>[Do not read out the options. Keep asking what else.	a. গর্ভকালীন সময়ে আপনার নিজের যত্ন নিতে সহায়তা করতে<br>Supporting in self care during pregnancy ....A	1	2	3	
		b. প্রসব পরিকল্পনা গ্রহনে অংশগ্রহন করতে<br>Participation in BPCR........................B	1	2	3	
		c. গর্ভকালীন চেকআপের সময় উপস্থিত থাকতে Being present during ANC visit C	1	2	3	
		d. প্রসবকালীন সময়ে উপস্থিত থাকতে<br>Being present during delivery ....D	1	2	3	
		e. প্রসব-পরবর্তী চেকআপের সময় উপস্থিত থাকতে<br>Being present during PNC visit .... E	1	2	3	

NO	Questions And Filters	Coding Categories	Skip
	Record all the answers first. Then read out the remaining options]<br>f. নবজাতকের যত্নে অংশগ্রহন করতে<br>Participation in newborn care .... F<br>x. অন্যান্য Others X	1 | 2 | 3<br>উল্লেখ করুন<br>Specify ________________________	
w59.	শেষ গর্ভের সময় আপনার নিজের যত্ন নিতে আপনার স্বামী আপনাকে কতটুকু সহায়তা করেছিল?<br>How supportive was your husband in assisting you to take care of yourself during pregnancy?	খুব সহায়ক Very supportive.................................... 1<br>মোটামুটি সহায়ক Somewhat supportive .... 2<br>একদম সহায়ক নয় Not supportive at all.................................... 3<br>জানি না/ মনে নাই Don’t know/Can’t remember.................... 9	3/9 → w65
	শেষ গর্ভের সময় আপনার নিজের যত্ন নিতে আপনার স্বামী আপনাকে কি ধরনের সহায়তা করেছিল? (নিচের প্রশ্নগুলো পড়ে শোনান: w60- w64)<br>What kind of support did your husband provide during your most recent pregnancy? (Read out the following questions w60- w64)		
w60.	ভালো খাবার খেতে<br>Eating well	হ্যাঁ Yes .... 1<br>না No.................................... 2<br>জানি না/ মনে নাই Don’t know/Can’t remember.................... 9	
w61.	কাজের পরিমান কমাতে<br>Reducing workload	হ্যাঁ Yes .... 1<br>না No.................................... 2<br>জানি না/ মনে নাই Don’t know/Can’t remember.................... 9	
w62.	সঠিকভাবে বিশ্রাম নিতে<br>Taking proper rest	হ্যাঁ Yes .... 1<br>না No.................................... 2<br>জানি না/ মনে নাই Don’t know/Can’t remember.................... 9	
w63.	স্বাস্থ্যসেবা নিতে<br>Seeking health services	হ্যাঁ Yes .... 1<br>না No.................................... 2<br>জানি না/ মনে নাই Don’t know/Can’t remember.................... 9	
w64.	প্রসব পরিকল্পনা করতে<br>Preparing BPCR	হ্যাঁ Yes .... 1<br>না No.................................... 2<br>জানি না/ মনে নাই Don’t know/Can’t remember.................... 9	
w65.	শেষ গর্ভের সময় আপনার নিজের যত্ন নিতে পরিবারের সদস্যরা আপনাকে কতটুক সহায়তা করেছিল?<br>How supportive were the other family members in assisting you to take care of yourself during pregnancy?	খুব সহায়ক Very supportive.................................... 1<br>মোটামুটি সহায়ক Somewhat supportive .... 2<br>একদম সহায়ক নয় Not supportive at all.................................... 3<br>জানি না/ মনে নাই Don’t know/Can’t remember.................... 9	3/9 → w71
	শেষ গর্ভের সময় আপনার নিজের যত্ন নিতে আপনার পরিবারের সদস্যরা আপনাকে কি ধরনের সহায়তা করেছিল? (নিচের প্রশ্নগুলো পড়ে শোনান: w66-w70)<br>What kind of support did they provide during your most recent pregnancy? (read out the following questions: w66-w70)		
w66.	ভালো খাবার খেতে<br>Eating well	হ্যাঁ Yes .... 1<br>না No.................................... 2<br>জানি না/ মনে নাই Don’t know/Can’t remember.................... 9	
w67.	কাজের পরিমান কমাতে<br>Reducing workload	হ্যাঁ Yes .... 1<br>না No.................................... 2<br>জানি না/ মনে নাই Don’t know/Can’t remember.................... 9	
w68.	পরিমিত বিশ্রাম নিতে<br>Taking proper rest	হ্যাঁ Yes .... 1<br>না No.................................... 2<br>জানি না/ মনে নাই Don’t know/Can’t remember.................... 9	
w69.	স্বাস্থ্য সেবা নিতে<br>Seeking health services	হ্যাঁ Yes .... 1<br>না No.................................... 2<br>জানি না/ মনে নাই Don’t know/Can’t remember.................... 9	
w70.	প্রসব পরিকল্পনা করতে<br>Preparing BPCR	হ্যাঁ Yes .... 1<br>না No.................................... 2<br>জানি না/ মনে নাই Don’t know/Can’t remember.................... 9	
w71.	শেষ গর্ভের সময়, আপনি ৭.১% ক্লোরহেক্সিডিন পেয়েছিলেন কি?<br>*[প্রয়োজনে মহিলাকে ক্লোরহেক্সিডিন এর বোতল দেখান]*<br>During your last pregnancy, were you given 7.1% chlorhexidine solution? [If necessary then show the chlorhexidine and then ask]	হ্যাঁ Yes .... 1<br>না No.................................... 2<br>জানি না/ মনে নাই Don’t know/Can’t remember.................... 9	
w72.	শেষ গর্ভের সময় প্রসব পরিকল্পনা করার জন্য আপনি কোন স্বাস্থ্যসেবাপ্রদানকারী সাথে আলাপ/পরামর্শ করেছিলেন কি?<br>Did you discuss about birth preparedness and complication readiness with a healthcare provider?	হ্যাঁ Yes .... 1<br>না No.................................... 2<br>জানি না/ মনে নাই Don’t know/Can’t remember.................... 9	2/9 → w75

NO	Questions And Filters	Coding Categories					Skip
w73.	কার কার সাথে এই প্রসব পরিকল্পনার বিষয়ে আলাপ/পরামর্শ করেছিলেন?<br>(কোড লিস্ট থেকে কোড লিখুন)<br>With whom did you discuss about birth and emergency preparedness?<br>(Fill out from the codebook provided-Person)	ব্যাক্তির কোড<br>Person code<br>|____||____| |____||____| |____||____| |___||____| 1 2 3 4					
w74.	প্রসব পরিকল্পনার কোন কোন বিষয়ে আপনি স্বাস্থ্যসেবপ্রদানকারীর সাথে আলোচনা করেছিলেন?<br>[উত্তরগুলো পড়ে শুনাবেন না। জিজ্ঞেস করুন, আরও কিছু? উত্তরদাতার নিজে থেকে দেয়া সবগুলো উত্তরই প্রথমেই বৃত্তায়িত করুন। এরপর বাকি উত্তরগুলো পড়ে শুনান]<br>What aspects of birth preparedness and complication readiness did you discuss with the health worker?<br>[Do not read out the options. Keep asking what else. Record all the answers first. Then read out the remaining options]			U-Yes	P-Yes	No	
		ডেলিভারীর স্থান নির্বাচনে<br>Identifying a place for delivery	A	1	2	3	
		প্রসব পরিচালনাকারী নির্বাচনে<br>Identifying a birth attendant	B	1	2	3	
		টাকা জমাতে Saving Money	C	1	2	3	
		জরুরী পরিবহন পেতে<br>Arranging transportation	D	1	2	3	
		সম্ভাব্য রক্তদাতা খুঁজতে<br>Arranging for a potential blood donor	E	1	2	3	
		গর্ভাবস্থায় সমস্যা হলে কোন হাসপাতালে যেতে হবে<br>Identifying a hospital for seeking care during delivery complication	F	1	2	3	
		সমস্যা হলে হাসপাতালে আপনার সঙ্গে কে যাবে<br>Identifying a companion during seeking care in hospital for delivery complication	G	1	2	3	
		অন্যান্য Others	X	উল্লেখ করুন<br>Specify<br>____________________			
w75.	শেষ গর্ভের সময় প্রসব পরিকল্পনার জন্য আপনি আপনার স্বামীর সাথে আলাপ/পরামর্শ করেছিলেন কি?<br>Did you discuss about birth preparedness and complication readiness with your husband during your most recent pregnancy?	হ্যাঁ Yes .... 1<br>না No .... 2<br>জানি না/ মনে নাই Don't know/Can't remember 9					**2/9 → w78**
w76.	আপনার স্বামীর সাথে আপনি প্রসব পরিকল্পনার কোন কোন বিষয়ে আলাপ/পরামর্শ করেছিলেন?<br>[উত্তরগুলো পড়ে শুনাবেন না। জিজ্ঞেস করুন, আরও কিছু? উত্তরদাতার নিজে থেকে দেয়া সবগুলো উত্তরই প্রথমেই বৃত্তায়িত করুন। এরপর বাকি উত্তরগুলো পড়ে শুনান]<br>What aspects of birth preparedness and complication readiness did you discuss with your husband?<br>[Do not read out the options. Keep asking what else. Record all the answers first. Then read out the remaining options]			U-Yes	P-Yes	No	
		ডেলিভারীর স্থান নির্বাচনে<br>Identifying a place for delivery	A	1	2	3	
		প্রসব পরিচালনাকারী নির্বাচনে<br>Identifying a birth attendant	B	1	2	3	
		টাকা জমাতে Saving Money	C	1	2	3	
		জরুরী পরিবহন পেতে<br>Arranging transportation	D	1	2	3	
		সম্ভাব্য রক্তদাতা খুঁজতে<br>Arranging for a potential blood donor	E	1	2	3	
		গর্ভাবস্থায় সমস্যা হলে কোন হাসপাতালে যেতে হবে<br>Identifying a hospital for seeking care during delivery complication	F	1	2	3	

<table>
<tr><th>NO</th><th>Questions And Filters</th><th colspan="5">Coding Categories</th><th>Skip</th></tr>
<tr><td></td><td></td><td>সমস্যা হলে হাসপাতালে আপনার সঙ্গে কে যাবে
Identifying a companion during seeking care in hospital for delivery complication</td><td>G</td><td>1</td><td>2</td><td>3</td><td></td></tr>
<tr><td></td><td></td><td>অন্যান্য Others</td><td>X</td><td colspan="4">উল্লেখ করুন
Specify
________________________</td></tr>
<tr><td>w77.</td><td>শেষ গর্ভের সময় প্রসব পরিকল্পনার ক্ষেত্রে আপনার স্বামী সক্রিয় ভূমিকা রেখেছিলেন কি?
Was your husband actively involved in birth preparedness and complication readiness during your most recent pregnancy?</td><td colspan="5">হ্যাঁ Yes 1
না No 2
জানি না/ মনে নাই Don't know/Can't remember .... 9</td><td></td></tr>
<tr><td>w78.</td><td>শেষ গর্ভের সময় প্রসব পরিকল্পনার জন্য আপনি স্বামী ছাড়া পরিবারের অন্য কারো সাথে আলাপ/পরামর্শ করেছিলেন কি?
Did you discuss about birth preparedness and complication readiness with any of your family members other than your husband?</td><td colspan="5">হ্যাঁ Yes 1
না No 2
জানি না/ মনে নাই Don't know/Can't remember .... 9</td><td>2/9 → w81</td></tr>
<tr><td>w79.</td><td>কার কার সাথে পরামর্শ করেছিলেন
<u>[জিজ্ঞেস করুন, আরও কিছু? উত্তরদাতার নিজে থেকে দেয়া সবগুলো উত্তরই বৃত্তায়িত করুন । উত্তরগুলো পড়ে শুনাবেন না। একাধিক উত্তর হতে পারে।]</u>
With whom did you discuss this?
<u>[Do not read out the options. Keep asking what else. Record all the answers.]</u></td><td colspan="5">শ্বশুর Father in law .... A
শ্বাশুড়ী Mother in law .... B
বাবা Father C
মা Mother .... D
ভাসুর Brother in law .... E
ননদ / Sister in law .... F
অন্যান্য Others ____________________ .... X</td><td></td></tr>
<tr><td>w80.</td><td>প্রসব পরিকল্পনার কোন কোন বিষয়ে আপনি পরিবারের অন্যান্য সদস্যদের (স্বামী ব্যতীত) সাথে আলোচনা করেছিলেন?
<u>[উত্তরগুলো পড়ে শুনাবেন না। জিজ্ঞেস করুন, আরও কিছু? উত্তরদাতার নিজে থেকে দেয়া সবগুলো উত্তরই প্রথমেই বৃত্তায়িত করুন। এরপর বাকি উত্তরগুলো পড়ে শুনান]</u>
What aspects of birth and emergency planning did you discuss with the other family members (except husband)?
<u>[Do not read out the options. Keep asking what else. Record all the answers first. Then read out the remaining options]</u></td><td></td><td></td><td>U-Yes</td><td>P-Yes</td><td>No</td><td></td></tr>
<tr><td></td><td></td><td>ডেলিভারীর স্থান নির্বাচনে
Identifying a place for delivery</td><td>A</td><td>1</td><td>2</td><td>3</td><td></td></tr>
<tr><td></td><td></td><td>প্রসব পরিচালনাকারী নির্বাচনে
Identifying a birth attendant</td><td>B</td><td>1</td><td>2</td><td>3</td><td></td></tr>
<tr><td></td><td></td><td>টাকা জমাতে Saving Money</td><td>C</td><td>1</td><td>2</td><td>3</td><td></td></tr>
<tr><td></td><td></td><td>জরুরী পরিবহন পেতে Arranging transportation</td><td>D</td><td>1</td><td>2</td><td>3</td><td></td></tr>
<tr><td></td><td></td><td>সম্ভাব্য রক্তদাতা খুঁজতে Arranging for a potential blood donor</td><td>E</td><td>1</td><td>2</td><td>3</td><td></td></tr>
<tr><td></td><td></td><td>গর্ভাবস্থায় সমস্যা হলে কোন হাসপাতালে যেতে হবে
Identifying a hospital for seeking care during delivery complication</td><td>F</td><td>1</td><td>2</td><td>3</td><td></td></tr>
<tr><td></td><td></td><td>সমস্যা হলে হাসপাতালে আপনার সঙ্গে কে যাবে
Identifying a companion during seeking care in hospital for delivery complication</td><td>G</td><td>1</td><td>2</td><td>3</td><td></td></tr>
<tr><td></td><td></td><td>অন্যান্য Others</td><td>X</td><td colspan="3">উল্লেখ করুন
Specify
__________________</td><td></td></tr>
<tr><td>w81.</td><td>শেষ গর্ভের সময় আপনি কোন এএনসি/পিএনসি/বিপিসিআর কার্ড পেয়েছিলেন কি?
<u>(নমুনা কার্ডটি দেখান। মায়ের কার্ডটি দেখাতে অনুরোধ করুন এবং নিচের প্রশ্নগুলো করুন)</u>
Did you receive any ANC/PNC/BPCR card during your pregnancy?
<u>(Request to show the Card and ask the following questions)</u></td><td colspan="5">হ্যাঁ Yes 1
না No 2
জানি না/ মনে নাই Don't know/Can't remember .... 9</td><td>2/9 → w90</td></tr>
</table>

NO	Questions And Filters	Coding Categories	Skip
w82.	কার কার কাছ থেকে আপনি কার্ডটি পেয়েছিলেন?<br>(কোড লিস্ট থেকে কোড লিখুন)<br>From whom did you receive the Card?<br>(Fill out from the codebook provided-Person)	ব্যাক্তির কোড Person code<br>|____||____| |____||____| |____||____| |____||____| 1 2 3 4	
w83.	আপনি কার্ডটি কোথা থেকে পেয়েছিলেন?<br>(কোড লিস্ট থেকে কোড লিখুন)<br>From where did you receive the Card?<br>(Fill out from the codebook provided-Place)	স্থানের কোড Place code<br>|____|____| |____|____| |____|____| |____|____| 1 2 3 4	
w84.	আপনি কার্ডটি প্রথম কখন পেয়েছিলেন?<br>When did you first get the card?	প্রথম ৩ মাসে 1st trimester (1-3 months) .... 1<br>দ্বিতীয় ৩ মাসে 2nd trimester (4-6 months) .... 2<br>তৃতীয় ৩ মাসে 3rd trimester (7-9 months).......... 3<br>জানি না/ মনে নাই Don't know/Can't remember 9	
w85.	গর্ভাবস্থায় কার্ডটি আপনি ব্যবহার করেছিলেন কি?<br>Did you use the card during your pregnancy?	হ্যাঁ Yes .... 1<br>না No .... 2<br>জানি না/ মনে নাই Don't know/Can't remember 9	**2/9 → w90**
w86.	কার্ডটি আপনার কাছে উপকারী ছিল বলে মনে হয়েছে কি?<br>Was the card useful/helpful?	হ্যাঁ Yes .... 1<br>না No .... 2<br>জানি না/ মনে নাই Don't know/Can't remember 9	
w87.	আপনি কি কার্ডটি প্রসব পরিকল্পনার জন্য ব্যবহার করেছিলেন?<br>Did you use the card for BPCR planning?	হ্যাঁ Yes .... 1<br>না No .... 2<br>জানি না/ মনে নাই Don't know/Can't remember 9	
w88.	কার্ডটির ব্যবহার সহজ ছিল বলে আপনি মনে করেন কি?<br>Was the card easy to use?	হ্যাঁ Yes .... 1<br>না No .... 2<br>জানি না/ মনে নাই Don't know/Can't remember 9	
w89.	কার্ডটি ব্যবহার করতে আপনি অন্য কোন গর্ভবতী মাকে পরামর্শ দিবেন?<br>Would you recommend other pregnant women to use this card?	হ্যাঁ Yes .... 1<br>না No .... 2<br>জানি না/ মনে নাই Don't know/Can't remember 9	
w90.	সন্তান জন্মদানের জন্য অথবা গর্ভাবস্থা/প্রসবকালীন জরুরী অবস্থা মোকাবিলায় স্বাস্থ্যকেন্দ্র যাওয়ার জন্য আগে থেকে যানবাহনের ব্যবস্থা করে রাখা হয়েছিল কি?<br>Was transport arranged in advance for going the place of birth or for emergency complications during pregnancy or childbirth?	হ্যাঁ Yes .... 1<br>না No .... 2<br>জানি না/ মনে নাই Don't know/Can't remember 9	**2/9 → w93**
w91.	ডেলিভারী অথবা জরুরী অবস্থায় স্বাস্থ্যকেন্দ্রে পৌছানোর জন্য যানবাহনের ব্যবস্থা কে করে রেখেছিলেন?<br>Who arranged the transport to reach health facility for birth or emergencies?	আমি নিজে Me .... 1<br>আমার স্বামী My husband.......... 2<br>উভয়ে Both of us .... 3<br>অন্য কেউ (উল্লেখ করুন)<br>Others (specify) ____________________ .... 7<br>জানা নাই/মনে নাই Don't know/can't remember.......... 9	
w92.	ডেলিভারী অথবা জরুরী অবস্থায় স্বাস্থ্যকেন্দ্রে পৌছানোর জন্য যানবাহনের ব্যবস্থা করে রাখার ব্যপারে কে সিদ্ধান্ত নিয়েছিলেন?<br>Who took the decision of arranging transport to reach health facility for birth or emergencies?	আমি নিজে Me .... 1<br>আমার স্বামী My husband.......... 2<br>উভয়ে Both of us .... 3<br>অন্য কেউ (উল্লেখ করুন)<br>Others (specify) ____________________ .... 7<br>জানা নাই/মনে নাই Don't know/can't remember.......... 9	
w93.	ডেলিভারী অথবা জরুরী অবস্থায় স্বাস্থ্যসেবার সম্ভাব্য খরচ মেটানোর জন্য আপনি অথবা অন্য কেউ কি আগে থেকেই টাকা পয়সা জমা করে রেখেছিলেন?<br>Did you or anybody else save money for potential cost associated with birth or treatment for emergencies?	হ্যাঁ Yes .... 1<br>না No .... 2<br>জানি না/ মনে নাই Don't know/Can't remember 9	**2/9 → w96**
w94.	ডেলিভারী অথবা জরুরী অবস্থায় স্বাস্থ্যসেবার সম্ভাব্য খরচ মেটানোর জন্য কে আগে থেকেই টাকা পয়সা জমা করে রেখেছিলেন?<br>Who arranged/saved the money in advance?	আমি নিজে Me .... 1<br>আমার স্বামী My husband.......... 2<br>উভয়ে Both of us .... 3<br>অন্য কেউ (উল্লেখ করুন)<br>Others (specify) ____________________ .... 7<br>জানা নাই/মনে নাই Don't know/can't remember.......... 9	
w95.	ডেলিভারী অথবা জরুরী অবস্থায় স্বাস্থ্যসেবার সম্ভাব্য খরচ মেটানোর জন্য টাকা সঞ্চয়ের ব্যপারে মূলত কে সিদ্ধান্ত নিয়েছিলেন?<br>Who took decision about saving money?	আমি নিজে Me .... 1<br>আমার স্বামী My husband.......... 2<br>উভয়ে Both of us .... 3<br>অন্য কেউ (উল্লেখ করুন)<br>Others (specify) ____________________ .... 7<br>জানা নাই/মনে নাই Don't know/can't remember.......... 9	

NO	Questions And Filters	Coding Categories	Skip
w96.	ডেলিভারী অথবা জরুরী অবস্থার সময় যদি রক্তের প্রয়োজন হয়, সে জন্য আগে থেকেই একজন রক্তদাতা নির্বাচন করে করে রাখা হয়েছিল কি?<br>Did you or anybody else identify a potential blood donor before delivery?	হ্যাঁ Yes .... 1<br>না No.. 2<br>জানি না/ মনে নাই Don't know/Can't remember.................... 9	**2/9 →w 101**
w97.	কে আগে থেকেই রক্তদাতা নির্বাচন করে রেখেছিলেন?<br>Who arranged the blood donor in advance for birth and emergencies?	আমি নিজে Me .... 1<br>আমার স্বামী My husband.. 2<br>উভয়ে Both of us... 3<br>অন্য কেউ (উল্লেখ করুন)<br>Others (specify) ______________________ .... 7<br>জানা নাই/মনে নাই Don't know/can't remember.................... 9	
w98.	রক্তদাতা নির্বাচন করার ক্ষেত্রে মূলত কে সিদ্ধান্ত নিয়েছিলেন?<br>Who took the decision about identifying potential blood donor?	আমি নিজে Me .... 1<br>আমার স্বামী My husband.. 2<br>উভয়ে Both of us... 3<br>অন্য কেউ (উল্লেখ করুন)<br>Others (specify) ______________________ .... 7<br>জানা নাই/মনে নাই Don't know/can't remember.................... 9	
w99.	কতজন রক্তদাতা আগে থেকেই নির্বাচন করে রেখেছিলেন?<br>How many potential blood donors did you identify in advance?	|_____||_____| জন persons	
w100.	রক্তদাতা হিসেবে কাকে কাকে নির্বাচন করে করে রাখা হয়েছিল?<br>[জিজ্ঞেস করুন, আরও কিছু? উত্তরদাতার নিজে থেকে দেয়া সবগুলো উত্তরই বৃত্তায়িত করুন । উত্তরগুলো পড়ে শুনাবেন না। একাধিক উত্তর হতে পারে।]<br>Whom did you identify?<br>[Do not read out the options. Keep asking what else. Record all the answers.]	একই খানার/পরিবারের লোকজন Household Member ....A<br>অন্যান্য রক্ত সম্পর্কের আত্মীয় Other blood relative.....................B<br>প্রতিবেশী Neighbour...C<br>অপরিচিত ব্যক্তি Unknown person...D<br>অন্যান্য Others (specify)____________________................X<br>জানি না/ মনে নাই Don't know/Can't remember....................Z	
w101.	আপনার শেষ গর্ভের সময়, কোন বিপদচিহ্ন দেখা দিলে কোথায় দেখাবেন/কোথা থেকে সেবা নিবেন, তা আগে থেকেই ঠিক করে রাখা হয়েছিল কি?<br>Did you or anybody else select a health facility in advance where you would have gone in case of any complication during pregnancy and delivery?	হ্যাঁ Yes .... 1<br>না No.. 2<br>জানি না/ মনে নাই Don't know/Can't remember.................... 9	**2/9 →w 104**
w102.	w102.a.<br>গর্ভকালীন/প্রসবকালীন জটিলতা দেখা দিলে কোথায় দেখাবেন/কোথা থেকে সেবা নিবেন তা কে আগে থেকেই ঠিক করে রেখেছিল?<br>Who selected the health facility in advance where you would have gone in case of any complication during pregnancy and childbirth?	আমি নিজে Me .... 1<br>আমার স্বামী My husband.. 2<br>উভয়ে Both of us... 3<br>অন্য কেউ (উল্লেখ করুন)<br>Others (Please mention) ______________________..... 7<br>জানা নাই/মনে নাই Don't know/can't remember.................... 9	
	w102.b.<br>গর্ভকালীন/প্রসবকালীন জটিলতা দেখা দিলে কোথায় দেখাবেন তার সিদ্ধান্ত মূলত কে নিয়েছিলেন?<br>Who took the decision of selecting the health facility in advance where you would have gone in case of any complication during pregnancy and childbirth?	আমি নিজে Me .... 1<br>আমার স্বামী My husband.. 2<br>উভয়ে Both of us... 3<br>অন্য কেউ (উল্লেখ করুন)<br>Others (Please mention) ______________________..... 7<br>জানা নাই/মনে নাই Don't know/can't remember.................... 9	
w103.	কোথায় দেখাবেন বলে ঠিক করে রাখা হয়েছিল?<br>(কোড লিস্ট থেকে কোড লিখুন)<br>Which health facility did you select to go to in the case of complications during pregnancy and childbirth?<br>(Fill out from the codebook provided-Place)	স্থানের কোড<br>Place code<br>|____|____| |____|____| |____|____| |____|____| 1 2 3 4	
w104.	সন্তান প্রসব মূলত কে করাবে - তা আপনি অথবা অন্য কেউ আগে থেকে ঠিক করে রেখেছিলেন কি?<br>Did you or anybody else select the person in advance to conduct the childbirth?	হ্যাঁ Yes .... 1<br>না No.. 2<br>জানি না/ মনে নাই Don't know/Can't remember.................... 9	**2/9 →w 107**
w105.	w105.a.<br>সন্তান প্রসব মূলত কে করাবে তা আগে থেকে কে ঠিক করে রেখেছিল?<br>Who selected the person in advance to conduct the childbirth?	আমি নিজে Me .... 1<br>আমার স্বামী My husband.. 2<br>উভয়ে Both of us... 3<br>অন্য কেউ (উল্লেখ করুন)<br>Others (Please mention) ______________________..... 7<br>জানা নাই/মনে নাই Don't know/can't remember.................... 9	

NO	Questions And Filters	Coding Categories	Skip
	w105.b.<br>সন্তান প্রসব মূলত কে করাবে - আগে থেকে তার সিদ্ধান্ত কে নিয়েছিলেন?<br>Who primarily took the decision of selecting the person to conduct the childbirth?	আমি নিজে Me .... 1<br>আমার স্বামী My husband........ 2<br>উভয়ে Both of us........ 3<br>অন্য কেউ (উল্লেখ করুন)<br>Others (Please mention) ____________ ..... 7<br>জানা নাই/মনে নাই Don't know/can't remember........ 9	
w106.	মূলত কাকে কাকে সন্তান প্রসব করানোর জন্য ঠিক করে রাখা হয়েছিল?<br>(কোড লিস্ট থেকে কোড লিখুন)<br>Whom did you identify to conduct the childbirth?<br>(Fill out from the codebook provided)	ব্যাক্তির কোড Person Code:<br>|___|___| |___|___| |___|___| |___|___| 1 2 3 4	
w107.	আপনার সন্তান জন্মদানের সময় আপনাকে মানসিক এবং সামাজিক সমর্থন যোগানোর জন্য কাউকে ঠিক করে রাখা হয়েছিল কি (সন্তান প্রসব করা অথবা সন্তান প্রসবে সহায়তা করা ছাড়া)?<br>Did you or some body else select someone as a companion of choice who can provide you emotional and social support during childbirth?	হ্যাঁ Yes .... 1<br>না No........ 2<br>জানি না/ মনে নাই Don't know/Can't remember........ 9	**2/9 →w 110**
w108.	আপনার সন্তান জন্মদানের সময় আপনাকে মানসিক এবং সামাজিক সমর্থন যোগানোর জন্য কে উপস্থিত থাকবে তার সিদ্ধান্ত কে নিয়েছিলেন?<br>Who primarily took the decision of selecting a companion of choice who can provide you emotional and social support during childbirth?	আমি নিজে Me .... 1<br>আমার স্বামী My husband........ 2<br>উভয়ে Both of us........ 3<br>শ্বশুর Father in law........ 4<br>শ্বাশুড়ী Mother in law .... 5<br>অন্য কেউ (উল্লেখ করুন)<br>Others (Please mention) ____________ ..... 7<br>জানা নাই/মনে নাই Don't know/can't remember........ 9	
w109.	কাকে কাকে ঠিক করে রাখা হয়েছিল?<br>[জিজ্ঞেস করুন, আরও কিছু? উত্তরদাতার নিজে থেকে দেয়া সবগুলো উত্তরই বৃত্তায়িত করুন । উত্তরগুলো পড়ে শুনাবেন না । একাধিক উত্তর হতে পারে ।]<br>Whom did you identify?<br>[Do not read out the options. Keep asking what else. Record all the answers.]	মা Mother ....A<br>শ্বাশুড়ী Mother-in-law........B<br>বোন/ননদ/জা Sister ....C<br>চাচী/মামী/খালা/ফুফুAunt ....D<br>দাদী/নানী Grandmother ....E<br>ভাগনি/ভাতিজী Niece........ F<br>স্বামী Husband ....G<br>অন্য কোন মহিলা আত্মীয় other female relative ....H<br>অন্য কোন পুরুষ আত্মীয় other male relative........ I<br>সনাতন দাই Traditional birth attendant........ J<br>অন্যান্য, নির্দিষ্ট করুন Others........X<br>(Specify)____________<br>জানি না/ মনে নাই Don't know/can't remember........ ....Z	
w110.	শেষ যখন আপনি গর্ভবতী ছিলেন, তখন (গর্ভবতী থাকাকালীন সময়ে) আপনার এই ডেলিভারীটি কোথায় হবে, তা আগে থেকেই ঠিক করে রাখা হয়েছিল কি?<br>Did you or anybody else select the place in advance where you will give the birth?	হ্যাঁ Yes .... 1<br>না No........ 2<br>জানি না/ মনে নাই Don't know/Can't remember........ 9	**2/9 →w 113**
w111.	w111a.<br>এই ডেলিভারীটি কোথায় হবে, তা আগে থেকেই কে ঠিক করে রেখেছিল?<br>Who primarily selected the place in advance where you would give birth	আমি নিজে Me .... 1<br>আমার স্বামী My husband........ 2<br>উভয়ে Both of us........ 3<br>অন্য কেউ (উল্লেখ করুন)<br>Others (Please mention) ____________ ..... 7<br>জানা নাই/মনে নাই Don't know/can't remember........ 9	
	w111b.<br>সন্তান কোথায় প্রসব করানো হবে তার সিদ্ধান্ত মূলত কে নিয়েছিলেন?<br>Who primarily took the decision of selecting the place in advance where you would give birth?	আমি নিজে Me .... 1<br>আমার স্বামী My husband........ 2<br>উভয়ে Both of us........ 3<br>অন্য কেউ (উল্লেখ করুন)<br>Others (Please mention) ____________ ..... 7<br>জানা নাই/মনে নাই Don't know/can't remember........ 9	
w112.	কোথায় সন্তান প্রসব হবে বলে ঠিক করে রাখা হয়েছিল?<br>Which place was selected?	স্থানের কোড<br>Place code<br>|___|___|	

NO	Questions And Filters	Coding Categories	Skip
w113.	শেষ গর্ভের সন্তান কোথায় প্রসব হয়েছিল?<br>(কোড লিস্ট থেকে কোড লিখুন)<br><br>Where did the most recent birth take place?<br>(Fill out from the codebook provoided)	স্থানের কোড<br>Place code<br><br>|____|____|	**Other than home delivery → w115**
w114.	[বাড়িতে সন্তান প্রসবের ক্ষেত্রে]<br><br>আপনি স্বাস্থ্যকেন্দ্রে প্রসব করাননি কেনো?<br><br>[জিজ্ঞেস করুন, আরও কিছু? উত্তরদাতার নিজে থেকে দেয়া সবগুলো উত্তরই বৃত্তায়িত করুন । উত্তরগুলো পড়ে শুনাবেন না। একাধিক উত্তর হতে পারে।]<br><br>[FOR HOME BASED CHILDBIRTH]<br><br>Why did you decide to give childbirth at home?<br><br>[Do not read out the options. Keep asking what else. Record all the answers.]	a. স্বাস্থ্যকেন্দ্রে সন্তান প্রসব কে গুরুত্বপূর্ন মনে হয়নি Didn't consider facility birth to be important........A<br>b. অর্থের সমস্যা Lack of money........B<br>c. সাথে যাওয়ার কেউ ছিলনা<br>No one to accompany to facility.. ....C<br>d. স্বামীর অনুমতি ছিলনা Husband did not permit to go to facility........D<br>e. পরিবারের অন্য সদস্যদের অসম্মতি ছিল<br>Other family members did not permit to go to facility....E<br>f. রাস্তা খারাপ Road condition was bad ....F<br>g. স্বাস্থ্সেবারদানকারীর সেবার মান নিয়ে আমি সন্তুষ্ট নই<br>Not happy with service provider's service ....G<br>h. ধর্মীয় কারন Religious reasons ....H<br>i. সামাজিক কারন Social reasons ....I<br>j. যানবাহনের অভাব Lack of transportation ....J<br>k. পেট কেটে বাচ্চা বের করার ভয় Fear of C-Section ....K<br>l. কোথায় যেতে হবে জানতাম না<br>Did not know where to go ....L<br>m. স্বাস্থ্য কেন্দ্রে মহিলা স্বাস্থ্যসেবাপ্রদানকারীর অভাব<br>Lack of female health service provider in the health facility ....M<br>x.অন্যান্য, নির্দিষ্ট করুন Others........X<br>(Specify)____________________	w 117

NO	Questions And Filters	Coding Categories	Skip
w115.	[স্বাস্থ্যসেবা কেন্দ্রে সন্তান প্রসবের ক্ষেত্রে]<br><br>সন্তান জন্মদানের জন্য কেন আপনি এই স্বাস্থ্যসেবা কেন্দ্র বেছে নিয়েছিলেন?<br><br>[জিজ্ঞেস করুন, আরও কিছু? উত্তরদাতার নিজে থেকে দেয়া সবগুলো উত্তরই বৃত্তায়িত করুন । উত্তরগুলো পড়ে শুনাবেন না। একাধিক উত্তর হতে পারে।]<br><br>[FOR FACILY BASED CHILDBIRTH]<br><br>Why did you choose this facilty for childbirth?<br><br>[Do not read out the options. Keep asking what else. Record all the answers.]	a. নিকটতম স্বাস্থ্যসেবা কেন্দ্র<br>Nearest facility ....A<br>b. সর্বোৎকৃষ্ট স্বাস্থ্যসেবাকেন্দ্র/পরিচ্ছন্ন পরিবেশ/ ভাল সেবা<br>Best facility/clean environment/goodservices ....B<br>c. একই স্বাস্থ্যকেন্দ্র যেখান থেকে গর্ভকালীন মেডিকেল চেকআপ করা হয়েছে<br>Same facility used for ANC ....C<br>d. অন্য কোথাও পাওয়া যায় না এমন সেবা<br>Similar services are not available elsewhere ....D<br>e. প্রসবের জন্য বাছাইকৃত হাসপাতাল<br>Hospital of choice for delivery ....E<br>f. সাশ্রয়ী মূল্যে সেবা<br>Affordable services .... F<br>g. আত্মীয়/বন্ধুদের সুপারিশকৃত<br>Recommended by relative/friend ....G<br>h. অন্য স্বাস্থ্য সেবাকেন্দ্রের ভীড় এড়ানোর জন্য<br>Avoid congestion in other facilities................................H<br>i. অন্য স্বাস্থ্য সেবাকেন্দ্র থেকে রেফারকৃত<br>Referred from another facility .... I<br>j. উত্তম সরঞ্জাম<br>Well equipped facility with supplies ....J<br>k. একমাত্র উপায়<br>only option ....K<br>l. বিশেষায়িত সেবাপ্রদানকারী স্বাস্থ্যকেন্দ্র<br>Facility offer specialized service ....L<br>m. জটিলতার জন্য<br>Because of complications...M<br>n. স্বাস্থ্য সেবাপ্রদানকারী ভাল সেবা দেয়<br>Providers treat clients well ....N<br>o. স্বামীর সিদ্ধান্ত<br>Decision made by spouse ....O<br>p. পছন্দের স্বাস্থ্যসেবা কেন্দ্র<br>Like the facility .... P<br>x. অন্যান্য, নির্দিষ্ট করুন others<br>specify____________________________.....................X<br>z. জানিনা/ মনে নাই DK/DR..Z	
w116.	<নাম> এর জন্য কি সিজার মানে পেট কেটে বাচ্চা বের করে, এভাবে হয়েছিল?<br>Did you have a caesarean section druing your last childbirth?	হ্যাঁ Yes .... 1<br>না No... 2<br>জানি না/ মনে নাই Don’t know/Can’t remember.................... 9	
w117.	শেষ গর্ভের ডেলিভারীটি মূলত কে করিয়েছিল?<br>(কোড লিস্ট থেকে কোড লিখুন)<br>Who conducted the childbirth?<br>(Only one answer. Fill out from the codebook provided)	ব্যাক্তির কোড Person Code:<br><br>|____|____|	
w118.	শেষ গর্ভের ডেলিভারীতে মূলত কে কে সাহায্য করেছিল?<br>(কোড লিস্ট থেকে কোড লিখুন)<br>Who primarily assisted you during your childbirth?<br>(Only one answer. Fill out from the codebook provided)	ব্যাক্তির কোড Person Code:<br><br>|____|____| |____|____| |____|____| |____|____| 1 2 3 4	
w119.	সন্তান জন্মদানের সময় আপনাকে মানসিক ও সামাজিক সমর্থন দেওয়ার জন্য আপনার পছন্দের কোন সঙ্গি সাথে ছিল কি?<br>Did you have a companion of choice who provided you emotional and social support during childbirth?	হ্যাঁ Yes .... 1<br>না No... 2<br>জানি না/ মনে নাই Don’t know/Can’t remember.................... 9	**2/9 →w 121**

NO	Questions And Filters	Coding Categories	Skip
w120.	সন্তান জন্মদানের সময় আপনাকে মানসিক ও সামাজিক সমর্থন দেওয়ার জন্য কে কে আপনার সাথে ছিল (সন্তান প্রসব করা অথবা সন্তান প্রসবে সহায়তা করা ছাড়া)?<br><br>[জিজ্ঞেস করুন, আরও কিছু? উত্তরদাতার নিজে থেকে দেয়া সবগুলো উত্তরই বৃত্তায়িত করুন । উত্তরগুলো পড়ে শুনাবেন না। একাধিক উত্তর হতে পারে।]<br>Who were present during the childbirth as a companion of choice who provided you emotional and social support (did not conduct the delivery or did not assist)?<br><br>[Do not read out the options. Keep asking what else. Record all the answers.]	মা Mother ....A<br>শ্বাশুড়ী Mother-in-law........B<br>বোন/ননদ/জা Sister ....C<br>চাচী/মামী/খালা/ফুফুAunt ....D<br>দাদী/নানী Grandmother ....E<br>ভাগনি/ভাতিজী Niece........F<br>স্বামী Husband ....G<br>অন্য কোন মহিলা আত্মীয় other female relative ....H<br>অন্য কোন পুরুষ আত্মীয় other alerelative ....I<br>অন্যান্য, নির্দিষ্ট করুন Others........X<br>(Specify)____________________<br>জানি না/ মনে নাই Don't know/Can't remember........Z	w 122
w121.	সন্তান জন্মদানের সময় আপনার সাথে কেন কোন পছন্দের সঙ্গি ছিল না?<br><br>[জিজ্ঞেস করুন, আরও কিছু? উত্তরদাতার নিজে থেকে দেয়া সবগুলো উত্তরই বৃত্তায়িত করুন । উত্তরগুলো পড়ে শুনাবেন না। একাধিক উত্তর হতে পারে।]<br><br>Why did you not have a companion of choice (apart from who conducted the childbirth and who assisted)?<br><br>[Do not read out the options. Keep asking what else. Record all the answers.]	a. প্রয়োজন মনে হয়নি Didn't consider it to be important.......A<br>b. স্বামীর অনুমতি ছিলনা Husband did not permit ....B<br>c. পরিবারের অন্য সদস্যদের অসম্মতি ছিল<br>Other family members did not permit ....C<br>d. স্বাস্থ্যসেবাকেন্দ্র/ স্বাস্থ্যসেবা প্রদানকারীর অসম্মতি ছিল Health facility/health care provider did ot permit ....D<br>e. পছন্দের সঙ্গি ছিলনা The companion of choice was not available ....E<br>f. ধর্মীয় কারন Religious reasons ....F<br>g. সামাজিক কারন Social reasons ....G<br>x.অন্যান্য, নির্দিষ্ট করুন Others........X<br>(Specify)__________________________	
w122.	w122a.<br>সন্তান জন্মের সময় আপনার স্বামী আপনার সাথে গিয়েছিলেন কি?<br>Did your husband travel with you during the childbirth?	হ্যাঁ Yes ....1<br>না No........2<br>জানি না/ মনে নাই Don't know/Can't remember........9	
	**w122b.**<br>সন্তান জন্মের সময় আপনার স্বামী উপস্থিত ছিলেন কি?<br>Was your husband present during the childbirth?	হ্যাঁ Yes ....1<br>না No........2<br>জানি না/ মনে নাই Don't know/Can't remember........9	**1→ w 124**
w123.	যদি উপস্থিত না থেকে থাকেন তবে কেন ছিলেন না?<br>If no, why not?<br>[Do not read out the options. Keep asking what else. Record all the answers.]	a. প্রয়োজন মনে হয়নি<br>Didn't consider it to be important ....A<br>b. স্বামীর ইচ্ছা ছিলনা<br>Husband did not want to be present ....B<br>c. আমি চাইনি আমার স্বামী উপস্থিত থাকুক<br>I did not want my husband to be present........C<br>d. পরিবারের অন্য সদস্যদের অসম্মতি ছিল<br>Other family members did not permit ....D<br>e.স্বাস্থ্যসেবাকেন্দ্র/ স্বাস্থ্যসেবা প্রদানকারীর অসম্মতি ছিল<br>Health facility/health care provider did ot permit ....E<br>f. স্বামী অন্য কাজে ব্যস্ত ছিল<br>Husband was busy for oher work ....F<br>g. ধর্মীয় কারন Religious reasons ....G<br>h. সামাজিক কারন Social reasons ....H<br>x.অন্যান্য Others ....X<br>নির্দিষ্ট করুন (specify) ____________________________	
w124.	প্রসব বেদনার সময় আপনাকে ওঠা বসা এবং হাঁটাচলা করতে দেওয়া হয়েছিল কি?<br>Were you allowed to get up and walk around while you were in labour?	হ্যাঁ Yes ....1<br>না No........2<br>জানি না/ মনে নাই Don't know/Can't remember........9	
w125.	সন্তান জন্মদানের সময় আপনাকে কোন পানিয় বা অন্য কিছু খাওয়ার অনুমতি দেওয়া হয়েছিল কি?<br>Were you allowed to drink liquids or eat any food while you were in labour?	হ্যাঁ Yes ....1<br>না No........2<br>জানি না/ মনে নাই Don't know/Can't remember........9	
w126.	সন্তান জন্মদানের সময় আপনি কোন অবস্থানে থাকতে চান সে ব্যপারে স্বাস্থ্যসেবাপ্রদানকারী আপনার কাছে জানতে চেয়েছিলেন কি?<br>Did a health care provider ask you what position you wanted to choose during your labour or for the childbirth?	হ্যাঁ Yes ....1<br>না No........2<br>জানি না/ মনে নাই Don't know/Can't remember........9	

NO	Questions And Filters	Coding Categories	Skip
w127.	সন্তান জন্মদানের সময় আপনি কোন অবস্থানে ছিলেন? চিৎ হয়ে, পাশ ফিরে, উবু হয়ে না কি অন্য ভাবে?<br>What position were you actually in when you delivered your baby? That is, were you on your back, on your hands and knees, squatting or in another position?	চিৎ হয়ে On back (lithotomy) .... 1<br>অর্ধ বসা Upright half sitting... 2<br>বাম কাত left lateral... 3<br>পাশ ফিরে in hands and knees....................................... 4<br>উবু হয়ে Squatting .... 5<br>দাঁড়িয়ে Standing .... 6<br>অন্যান্য, নির্দিষ্ট করুন other .... 7<br>(specify) _____________________	
w128.	এই অবস্থান কি আপনি ঠিক করে ছিলেন?<br>Was this position chosen by you?	হ্যাঁ Yes .... 1<br>না No.. 2<br>জানি না/ মনে নাই Don't know/Can't remember.................... 9	
w129.	প্রসব বেদনার সময় স্বাস্থ্যসেবাপ্রদানকারী আপনার শারিরীক পরীক্ষা করার আগে আপনার অনুমতি নিয়েছিলেন কি?<br>During labour did the healthcare provider ask your permission before carrying out physical examinations?	হ্যাঁ Yes .... 1<br>না No.. 2<br>জানি না/ মনে নাই Don't know/Can't remember.................... 9	
w130.	প্রসব বেদনার সময় স্বাস্থ্যসেবাপ্রদানকারী আপনার শারিরীক পরীক্ষা করার আগে কি করতে যাচ্ছে- তা আপনাকে বুঝিয়ে বলেছিলেন কি?<br>During labour did the healthcare provider explain what she was going to do before conducting the physical examinations?	হ্যাঁ Yes .... 1<br>না No.. 2<br>জানি না/ মনে নাই Don't know/Can't remember.................... 9	
w131.	এই সন্তান জন্মদানের কোন পর্যায়ে আপনার অনুমতি ছাড়া স্বাস্থ্যসেবাপ্রদানকারী কোন কিছু করেছিল কি?<br>At any point during your stay for this delivery did the healthcare provider perform any procedure without your permission?	হ্যাঁ Yes .... 1<br>না No.. 2<br>জানি না/ মনে নাই Don't know/Can't remember.................... 9	**2/9 → w 133**
w132.	কি করা হয়েছিল?<br>(উত্তরগুলো পড়ে শুনাবেন না। উত্তরদাতার নিজে থেকে দেয়া সবগুলো উত্তরই বৃত্তায়িত করুন। জিজ্ঞেস করুন, আরও কিছু?)<br>What was done? [Do not read out the options. Keep asking what else. Record all the answers.]	বন্ধ্যাত্ব করন Tubal ligation ....A<br>জরায়ু অপসারন Hysterectomy ....B<br>প্রসবের রাস্তার পার্শ্ব কেটে দেয়া Episiotomy ....C<br>সিজার মানে পেট কেটে বাচ্চা বের Caesarean section ....D<br>অন্যান্য, নির্দিষ্ট করুন Other , ...X<br>Specify________________________	
w133.	এই সন্তান জন্মদানের যে কোন পর্যায়ে যখন আপনার সেবার প্রয়োজন ছিল স্বাস্থ্যসেবাপ্রদানকারী কি তখন আপনাকে একা রেখে চলে গিয়েছিল?<br>At any point during your stay for this delivery were you left un attended by health providers when you needed care?	হ্যাঁ Yes .... 1<br>না No.. 2<br>জানি না/ মনে নাই Don't know/Can't remember.................... 9	
w134.	এই সন্তান জন্মদানের যে কোন পর্যায়ে আপনার সাথে এমন কোন কিছু/আচরন করা হয়েছে যেটিতে আপনার গোপনীয়তা ভঙ্গ হয়েছে?<br>At any point during your stay for this delivery were you treated in a way that violated your privacy?	হ্যাঁ Yes .... 1<br>না No.. 2<br>জানি না/ মনে নাই Don't know/Can't remember.................... 9	
w135.	এই সন্তান জন্মদানের যে কোন পর্যায়ে স্বাস্থ্যসেবাপ্রদানকারী এমন কোন শব্দ বা আচরন করেছিল যা আপনাকে অস্বস্তিতে ফেলেছিল অথবা এমন কোন আচরন করেছিল যাতে আপনি অপমানিত বা অসম্মানিত বোধ করেছিলেন?<br>At any point during your stay for this delivery did any health care provider talk or use a tone or facial expression that made you feel uncomfortable or any other kind of behavior that made you feel humiliated or disrespected?	হ্যাঁ Yes .... 1<br>না No.. 2<br>জানি না/ মনে নাই Don't know/Can't remember.................... 9	**2/9 → w 149**
w136.	কি করেছিল?<br>(উত্তরগুলো পড়ে শুনাবেন না। উত্তরদাতার নিজে থেকে দেয়া সবগুলো উত্তরই বৃত্তায়িত করুন। জিজ্ঞেস করুন, আরও কিছু?)<br>What exactly happened?<br>(DO NOT READ, Circle all that apply, prompt for any more)	চিৎকার করেছিল Shouted .... 1<br>ধমক দিয়েছিল Scolded .... 2<br>সেবা প্রদান না করার হুমকি দিয়েছিল<br>Threatened to withhold services .... 3<br>অপারেশন থিয়েটারে পাঠিয়ে দেওয়ার হুমকি দিয়েছিল<br>Threatened with going to theatre.................................... 4<br>অপমানজনক নাম ধরে ডেকেছিল<br>Called by insulting name.. 5<br>উপহাস করেছিল Laughed at or scorned................................ 6<br>অন্যান্য Other.. 7<br>নির্দিষ্ট করুন, specify __________________________	

NO	Questions And Filters	Coding Categories	Skip
	আপনার সাথে অপমানজনক বা অসম্মানজনক আচরন করার কারন কি বলে আপনি মনে করেন?<br>Why do you think the healthcare provider treated you with humiliation or disrespect?		
w137.	আপনার বয়স<br>Age	হ্যাঁ Yes .... 1<br>না No .... 2<br>জানি না/ মনে নাই Don't know/Can't remember 9	
w138.	আপনার লিঙ্গ<br>Sex	হ্যাঁ Yes .... 1<br>না No .... 2<br>জানি না/ মনে নাই Don't know/Can't remember 9	
w139.	আপনার ধর্ম<br>Religion	হ্যাঁ Yes .... 1<br>না No .... 2<br>জানি না/ মনে নাই Don't know/Can't remember 9	
w140.	আপনার আর্থসামাজিক অবস্থান<br>Socio-economic status	হ্যাঁ Yes .... 1<br>না No .... 2<br>জানি না/ মনে নাই Don't know/Can't remember 9	
w141.	শিক্ষাগত যোগ্যতা<br>Educational status	হ্যাঁ Yes .... 1<br>না No .... 2<br>জানি না/ মনে নাই Don't know/Can't remember 9	
w142.	স্বাস্থ্যসেবাপ্রদানকারীদের কম পারিশ্রমিক<br>Health workers are underpaid	হ্যাঁ Yes .... 1<br>না No .... 2<br>জানি না/ মনে নাই Don't know/Can't remember 9	
w143.	স্বাস্থ্যসেবাপ্রদানকারীদের অতিরিক্ত কাজের চাপ<br>Health workers are overworked	হ্যাঁ Yes .... 1<br>না No .... 2<br>জানি না/ মনে নাই Don't know/Can't remember 9	
w144.	স্বাস্থ্যসেবাপ্রদানকারীরা যত্নশীল নয়<br>Health workers are not caring	হ্যাঁ Yes .... 1<br>না No .... 2<br>জানি না/ মনে নাই Don't know/Can't remember 9	
w145.	স্বাস্থ্যসেবাপ্রদানকারীদের সঠিকভাবে তত্ত্বাবধান করা হয়না<br>Health workers are not properly supervised	হ্যাঁ Yes .... 1<br>না No .... 2<br>জানি না/ মনে নাই Don't know/Can't remember 9	
w146.	স্বাস্থ্যসেবাপ্রদানকারীরা এলাকার প্রতি দায়বদ্ধ নয়<br>Health workers are not accountable to the community	হ্যাঁ Yes .... 1<br>না No .... 2<br>জানি না/ মনে নাই Don't know/Can't remember 9	
w147.	অন্যান্য, নির্দিষ্ট করুন<br>Others, specify		
w148.	প্রসব অথবা সন্তান জন্মদানের যে কোন পর্যায়ে যখন আপনি অস্বস্তি, অসম্মানিত বা অপমানিত বোধ করেছিলেন তখন আপনি কি করেছিলেন?<br>If you felt uncomfortable, disrespected or humiliated at any point during the labour or childbirth, what did you do?	আনুষ্ঠানিক ভাবে অভিযোগ দাখিল করেছিলেন<br>Formally filed a complaint .... 1<br>সুপারভাইজার বা অন্যান্য কর্মকর্তার নিকট অভিযোগ করেছিলেন<br>Complained to a supervisor or other staff person. .... 2<br>কিছুই করেননি Took no action .... 3<br>অন্যান্য, নির্দিষ্ট করুন Other .... 7<br>(specify)________________________<br>জানি না/ মনে নাই Don't know/Can't remember 9	

NO	Questions And Filters		Unprompted Yes	Prompted Yes	No	Skip
w149.	কখনও কখনও গর্ভকালীন সময় কিছু বিপদ চিহ্ন দেখা যেতে পারে এবং তখন গর্ভবতীকে তাৎক্ষনিকভাবে স্বাস্থ্যকেন্দ্রে পাঠানো দরকার হয়।<br>গর্ভকালীন সময়ে কি কি বিপদচিহ্ন দেখা দিলে একজন মহিলাকে তাৎক্ষনিকভাবে হাসপাতালে পাঠাতে হয়?<br>[জিজ্ঞেস করুন, আরও কিছু? উত্তরদাতার নিজে থেকে দেয়া সবগুলো উত্তর প্রথমেই বৃত্তায়িত করুন । উত্তরগুলো পড়ে শুনাবেন না। একাধিক উত্তর হতে পারে। এরপর বাকি উত্তরগুলো পড়ে শুনান]<br>During pregnancy, women may encounter severe problems or	যোনীপথে রক্তস্রাব Vaginal Bleeding .... A	1	2	3	
		জ্বর Fever .... B	1	2	3	
		তলপেটে তীব্র ব্যথা Severe abdominal pain. C	1	2	3	
		মাথা ব্যথা / চোখে ঝাপসা দেখা<br>Headache/ Blurred Vision .... D	1	2	3	
		খিঁচুনী/ফিট Convulsions/fits .... E	1	2	3	
		দুর্গন্ধযুক্ত স্রাব<br>Foul smelling vaginal discharge .... F	1	2	3	
		গর্ভের বাচ্চার নড়াচড়া কমে যাওয়া/বন্ধ হওয়া<br>Fetal movement reduced/absent.......... G	1	2	3	

NO	Questions And Filters		Coding Categories			Skip
	illness and should go or be taken immediately to a health facility.<br>What types of symptoms would cause you to seek care from a health facility right away?<br>[Do not read out the options. Keep asking what else. Record all the answers first. Then read out the remaining options]	যোনীপথ দিয়ে ঘোলাটে/সবুজাভ কিছু বের হওয়া<br>Leaking brownish/greenish fluid from the vagina .... H	1	2	3	
		হাতে পানি আসা/আঙ্গুল ফুলে যাওয়া<br>Edema of hand/fingers...........................I	1	2	3	
		মুখে / পায়ে পানি আসা<br>Edema of face/ leg ....J	1	2	3	
		অন্যান্য<br>Other.. X	নির্দিষ্ট করুন<br>(specify) ________________________			
w150.	প্রসব কালীন মায়েদের কখনও কখনও কিছু মারাত্নক সমস্যা অথবা অসুস্থতা দেখা দিতে পারে যখন প্রসবকারী মাকে তাৎক্ষনিকভাবে স্বাস্থ্যকেন্দ্রে পাঠানো দরকার হয়।<br>ডেলিভারীরর সময়ে কি কি বিপদচিহ্ন দেখা দিলে একজন মহিলাকে তাৎক্ষনিকভাবে হাসপাতালে পাঠাতে হয়?<br>[জিজ্ঞেস করুন, আরও কিছু? উত্তরদাতার নিজে থেকে দেয়া সবগুলো উত্তর প্রথমেই বৃত্তায়িত করুন । উত্তরগুলো পড়ে শুনাবেন না। একাধিক উত্তর হতে পারে। এরপর বাকি উত্তরগুলো পড়ে শুনান]<br>During childbirth, once contraction have started women may encounter severe problems or illness and should go or be taken immediately to a health facility.<br>While having contractions or delivering a baby, what types of symptoms would cause you to seek immediate care at a health facility right away?<br>[Do not read out the options. Keep asking what else. Record all the answers first. Then read out the remaining options]		Unprompted Yes	Prompted Yes	No	
		খিঁচুনী Convulsion .... A	1	2	3	
		তীব্র জ্বর Hight Fever............................... B	1	2	3	
		অতিরিক্ত রক্তস্রাব<br>Excessive Vaginal Bleeding C	1	2	3	
		দুর্গন্ধযুক্ত স্রাব<br>A bad smelling vaginal discharge .... D	1	2	3	
		ফুল না পড়া Retained Placenta ....E	1	2	3	
		তীব্র মাথা ব্যথা/চোখে ঝাপসা দেখা<br>Severe Headache/blurred vision ....F	1	2	3	
		দীর্ঘ / প্রলম্বিত প্রসব (12 ঘন্টার বেশী) ব্যথা থাকলে<br>prolonged labour (>12 hr)................... G	1	2	3	
		শিশুর হাত /পা আগে বের হয়ে এলে Hand or Feet came first .... H	1	2	3	
		অন্যান্য<br>Other.. X	উল্লেখ করুন<br>Specify __________________			
w151.	ডেলিভারীর পর একজন মায়ের কখনও কখনও এমন কিছু জটিলতা বা বিপদচিহ্ন দেখা যেতে পারে যে তখন তাকে অবিলম্বে চিকিৎসার জন্য হাসপাতালে/ ডাক্তারের কাছে নিয়ে যেতে হয়। ডেলিভারীর পর কি ধরনের লক্ষণ/ জটিলতা বা বিপদচিহ্ন দেখলে আপনি একজন মাকে চিকিৎসার জন্য হাসপাতালে বা স্বাস্থ্যকর্মীর কাছে নিয়ে যাবেন?<br>[জিজ্ঞেস করুন, আরও কিছু? উত্তরদাতার নিজে থেকে দেয়া সবগুলো উত্তর প্রথমেই বৃত্তায়িত করুন । উত্তরগুলো পড়ে শুনাবেন না। একাধিক উত্তর হতে পারে। এরপর বাকি উত্তরগুলো পড়ে শুনান]<br>Sometimes mothers after childbirth may have severe illness and should be taken immediately to a health facility.<br>What type of symptoms would cause you to go to a health facility right away?<br>[Do not read out the options. Keep asking what else. Record		Unprompted Yes	Prompted Yes	No	
		যোনীপথে অতিরিক্ত রক্তস্রাব<br>Excessive vaginal bleeding ....A	1	2	3	
		অতিরিক্ত জ্বর হলে High fever ....B	1	2	3	
		পেটে তীব্র ব্যথা Severe abdominal painC	1	2	3	
		তীব্র মাথা ব্যথা/ঝাপসা দেখা<br>Severe headache/blurry vision ....D	1	2	3	
		খিঁচুনী / অজ্ঞান<br>Convulsion/loss of conciousness ....E	1	2	3	
		যোনীপথে দূর্গন্ধযুক্ত স্রাব<br>Foul smelling vaginal discharge .....F	1	2	3	
		পায়ের পিছনে ব্যথা Pain in calf ....G	1	2	3	
		আচরনগত পরিবর্তন অর্থাৎ যেখানে মা নিজেকে বা বাচ্চাকে আঘাত করতে পারে<br>Behavior that indicates she may hurt herself or the baby ....H	1	2	3	
		স্তন/স্তনবৃন্তে ফোলা, লাল হওয়া বা ব্যথা<br>swollen, red, tender breasts or nipples I	1	2	3	

NO	Questions And Filters		Coding Categories			Skip
	all the answers first. Then read out the remaining options]	প্রস্রাব করায় অসুবিধা বা প্রস্রাব ঝরা<br>problems urinating, or leaking........J	1	2	3	
		পেরিনিয়াম এ ব্যাথা/ইনফেকশন<br>increased pain or infection in perineum...K	1	2	3	
		ক্ষতস্থানে ব্যাথা/লাল ভাব/ পুঁজ হওয়া<br>infection in the area of wound (redness, swelling, pain, or pus in wound site).....................L	1	2	3	
		অন্যান্য ,Other X	নির্দিষ্ট করুন<br>(specify)___________________________			
w152.	আপনি এই বিপদ চিহ্নগুলো সম্পর্কে কার কাছ থেকে জানতে পেরেছিলেন?<br>(কোড লিস্ট থেকে কোড লিখুন)<br><br>[জিজ্ঞেস করুন, আরও কিছু? উত্তরদাতার নিজে থেকে দেয়া সবগুলো উত্তরই লিখুন। উত্তরগুলো পড়ে শুনাবেন না। একাধিক উত্তর হতে পারে।]<br>From whom did you come to know about these danger signs?<br>(Fill out from the codebook provided)<br>[Do not readout the answers. Ask anything else? Record all the answers.]		ব্যাক্তির কোড<br>Person code<br><br>|____||____| |____||____| |____||____| |____||____| 1 2 3 4			
w153.	আপনি এই বিপদ চিহ্নগুলো সম্পর্কে কোথা থেকে জানতে পেরেছেন?<br>(কোড লিস্ট থেকে কোড লিখুন)<br><br>From where did you come to know about these danger signs?<br>(Fill out from the codebook provided)		স্থানের কোড<br>Place code<br><br>|____|____| |____|____| |____|____| |____|____| 1 2 3 4			

NO	Questions And Filters	P	D	AD	Skip
	আপনার শেষ গর্ভের গর্ভকালীন সময়ে, ডেলিভারীর সময় বা ডেলিভারীর পর আপনার কি নিম্নলিখিত সমস্যা গুলো হয়েছিল?<br>প্রত্যেকটি সমস্যা পড়ে শুনান:<br>Did you have any of the following problems during your recent pregnancy, childbirth or after birth? Read aloud the responses:				
		P<br>(গর্ভাবস্থা)	D<br>(ডেলিভারীর সময়)	AD<br>(ডেলিভারীর পর)	
w154.	তীব্র মাথা ব্যথাসহ চোখে ঝাপসা দেখা Blurred vision with severe headache	A	A	A	**All Y→ w 177**
w155.	খিঁচুনী/একলামশিয়া/অজ্ঞান হওয়া Convulsion/eclampsia /unconscious	B	B	B	
w156.	উচ্চ রক্তচাপ High blood pressure	C	C	C	
w157.	অতিরিক্ত রক্তস্রাব Excessive vaginal bleeding	D	D	D	
w158.	পানি ভাঙ্গার ৬ ঘন্টা পরও প্রসব না হওয়া<br>Not delivered even after 6 hours of membrane rupture	E			
w159.	বাচ্চার মাথা ছাড়া শরীরের অন্য অংশ আগে আসা<br>Delivery of parts of the baby other than head		F		
w160.	১২ ঘন্টার অধিক প্রসব ব্যথা More than 12 hours of labour pain		G		
w161.	ফুল না পড়া Retained placenta		H	H	
w162.	দুর্গন্ধযুক্ত স্রাবের সাথে তীব্র জ্বর Foul smelling discharge with fever	I		I	
w163.	হাতে/পায়ে/শরীরে পানি আসা Edema of hand/feet/body	J	J	J	
w164.	অন্যান্য, নির্দিষ্ট করুন Others, specify ____________________	X	X	X	
w165.	উপরে উল্লেখিত সমস্যাগুলোর কোনটিই হয়নি<br>None of the above mentioned problems	Y	Y	Y	
w166.	এই সমস্যা/জটিলতার জন্য আপনি কি কাউকে দেখিয়েছিলেন বা কারও সাহায্য নিয়েছিলেন?<br>Did you seek any sort of treatment for this problem/complication?	হ্যাঁ Yes = 1<br>না No = 2<br>জানি না/ মনে নাই<br>DK/DR = 9	হ্যাঁ Yes = 1<br>না No = 2<br>জানি না/ মনে নাই<br>DK/DR = 9	হ্যাঁ Yes = 1<br>না No = 2<br>জানি না/ মনে নাই<br>DK/DR = 9	**2/9 → w 177**

NO	Questions And Filters	Coding Categories			Skip
w167.	এই সমস্যা/জটিলতার জন্য আপনি কাকে কাকে দেখিয়েছিলেন বা কার কার সেবা নিয়েছিলেন?<br>(কোড লিস্ট থেকে কোড লিখুন)<br>From whom did you seek treatment for this problem/complication?<br>(Fill out from the codebook provided)	ব্যাক্তির কোড<br>Person Code:<br>1. |____||____| 2. |____||____| 3. |____||____| 4. |____||____|	ব্যাক্তির কোড<br>Person Code:<br>1. |____||____| 2. |____||____| 3. |____||____| 4. |____||____|	ব্যাক্তির কোড<br>Person Code:<br>1. |____||____| 2. |____||____| 3. |____||____| 4. |____||____|	
w168.	w168a.<br>এই সমস্যা/জটিলতার জন্য আপনি কোথায় দেখিয়েছিলেন বা কোথায় সেবা পেয়েছিলেন?<br>(কোড লিস্ট থেকে কোড লিখুন)<br>Where did you go to seek care for this problem/complication?<br>(Fill out from the codebook provided)	স্থানের কোড<br>Place code<br>1. |____||____| 2. |____||____| 3. |____||____| 4. |____||____|	স্থানের কোড<br>Place code<br>1. |____||____| 2. |____||____| 3. |____||____| 4. |____||____|	স্থানের কোড<br>Place code<br>1. |____||____| 2. |____||____| 3. |____||____| 4. |____||____|	
	w168b.<br>এই সমস্যা/জটিলতার জন্য সেবা নেওয়ার সময় আপনার স্বামী আপনার সাথে গিয়েছিলেন কি?<br>Did your husband travel with you during the care seeking for this problem/complicatione?	হ্যাঁ Yes = 1<br>না No = 2<br>জানি না/<br>মনে নাই<br>DK/DR = 9	হ্যাঁ Yes = 1<br>না No = 2<br>জানি না/<br>মনে নাই<br>DK/DR = 9	হ্যাঁ Yes = 1<br>না No = 2<br>জানি না/<br>মনে নাই<br>DK/DR = 9	
	w168c.<br>এই সমস্যা/জটিলতার জন্য সেবা নেওয়ার সময় আপনার স্বামী আপনার সাথে উপস্থিত ছিলেন কি?<br>Did your husband present with you during the care seeking for this problem/complicatione?	হ্যাঁ Yes = 1<br>না No = 2<br>জানি না/<br>মনে নাই<br>DK/DR = 9	হ্যাঁ Yes = 1<br>না No = 2<br>জানি না/<br>মনে নাই<br>DK/DR = 9	হ্যাঁ Yes = 1<br>না No = 2<br>জানি না/<br>মনে নাই<br>DK/DR = 9	
w169.	স্বাস্থ্যসেবাপ্রদানকারী আপনার শারীরিক পরীক্ষা করার আগে আপনার অনুমতি নিয়েছিলেন কি?<br>When seeking care for complication did the health care provider ask your permission before carrying out physical examinations?	হ্যাঁ Yes = 1<br>না No = 2<br>জানি না/<br>মনে নাই<br>DK/DR = 9	হ্যাঁ Yes = 1<br>না No = 2<br>জানি না/<br>মনে নাই<br>DK/DR = 9	হ্যাঁ Yes = 1<br>না No = 2<br>জানি না/<br>মনে নাই<br>DK/DR = 9	
w170.	স্বাস্থ্যসেবাপ্রদানকারী আপনার শারিরীক পরীক্ষা করার আগে কি করতে যাচ্ছে- তা আপনাকে বুঝিয়ে বলেছিলেন কি?<br>When seeking care for complication did the health care provider explain physical examinations to you before conducting them?	হ্যাঁ Yes = 1<br>না No = 2<br>জানি না/<br>মনে নাই<br>DK/DR = 9	হ্যাঁ Yes = 1<br>না No = 2<br>জানি না/<br>মনে নাই<br>DK/DR = 9	হ্যাঁ Yes = 1<br>না No = 2<br>জানি না/<br>মনে নাই<br>DK/DR = 9	
w171.	জটিলতার জন্য সেবা নেওয়ার সময় আপনার সাথে এমন কোন কিছু/আচরন করা হয়েছে যেটিতে আপনার গোপনীয়তা ভঙ্গ হয়েছে?<br>When seeking care for complication did any healthcare provider treat you in a way that violated your privacy?	হ্যাঁ Yes = 1<br>না No = 2<br>জানি না/<br>মনে নাই<br>DK/DR = 9	হ্যাঁ Yes = 1<br>না No = 2<br>জানি না/<br>মনে নাই<br>DK/DR = 9	হ্যাঁ Yes = 1<br>না No = 2<br>জানি না/<br>মনে নাই<br>DK/DR = 9	
w172.	জটিলতার জন্য সেবা নেওয়ার সময় স্বাস্থ্যসেবাপ্রদানকারী এমন কোন শব্দ বা আচরন করেছিল যা আপনাকে অস্বস্তিতে ফেলেছিল অথবা এমন কোন আচরন করেছিল যাতে আপনি অপমানিত বা অসম্মানিত বোধ করেছিলেন?<br>When seeking care for complication did any healthcare provider talk or use a tone or facial expression that made feel uncomfortable or any other kind of behavior that made you feel humiliated or disrespected?	হ্যাঁ Yes = 1<br>না No = 2<br>জানি না/<br>মনে নাই<br>DK/DR = 9	হ্যাঁ Yes = 1<br>না No = 2<br>জানি না/<br>মনে নাই<br>DK/DR = 9	হ্যাঁ Yes = 1<br>না No = 2<br>জানি না/<br>মনে নাই<br>DK/DR = 9	
w173.	গর্ভকালীন, ডেলিভারীর সময় বা ডেলিভারীর পর জটিলতার জন্য সেবা নেওয়ার সময় আপনি কখনো কোন যানবাহন ব্যবহার করেছিলেন?<br>During pregnancy, childbirth or after childbirth seeking care for complications, did you use any transportation ever?	হ্যাঁ Yes .... 1<br>না No .... 2<br>জানি না/ মনে নাই Don't know/Can't remember 9			**2/9 → w 177**

NO	Questions And Filters	Coding Categories			Skip
w174.	কি কি ধরনের যানবাহন ব্যবহার করেছিলেন?<br>[জিজ্ঞেস করুন, আরও কিছু? উত্তরদাতার নিজে থেকে দেয়া সবগুলো উত্তরই বৃত্তায়িত করুন । উত্তরগুলো পড়ে শুনাবেন না। একাধিক উত্তর হতে পারে।]<br>Which transport did you use?<br>[Do not read out the options. Keep asking what else. Record all the answers.]	a. রিক্সা/ভ্যান গাড়ি Rikshaw/Van ....A<br>b. নৌকা Boat ....B<br>c. ট্রলার/ ইঞ্জিন নৌকা<br>Trawler/Engine Boat ....C<br>d. বাস/প্রাইভেট গাড়ি<br>Bus/Private Car ....D<br>e. ট্রলী/টেম্পু/ভটভটি/ সিএনজি<br>Tempo/Bhotbhoti/CNG ....E<br>f. পায়ে হেঁটে On foot ....F<br>x. অন্যান্য Others ....X<br>নির্দিষ্ট করুন (specify)________________________<br>z.জানি না/ মনে নাই Don't know/Can't remember ....Z			
w175.	গর্ভকালীন, ডেলিভারীর সময় বা ডেলিভারীর পর জটিলতার জন্য সেবা নেওয়ার সময় যানবাহন পেতে বা যোগাড় করতে এলাকার কোন লোক সাহায্য করেছিল কি?<br>Did the community help you to arrange the transport During pregnancy, childbirth or after childbirth?	হ্যাঁ Yes .... 1<br>না No .... 2<br>জানি না/ মনে নাই Don't know/Can't remember 9			**2/9 → w 177**
w176.	কিভাবে এলাকার লোক সাহায্য করেছিল?<br>[জিজ্ঞেস করুন, আরও কিছু? উত্তরদাতার নিজে থেকে দেয়া সবগুলো উত্তরই বৃত্তায়িত করুন । উত্তরগুলো পড়ে শুনাবেন না। একাধিক উত্তর হতে পারে।]<br>How did the community help?<br>[Do not read out the options. Keep asking what else. Record all the answers.]	নিজেদের যানবাহন দিয়ে<br>Provided Community Support Group's transport ....A<br>যানবাহন যোগাড় করতে Helped to arrange transport ....B<br>যানবাহনের খরচ প্রদানে Provided money for the transport ......C<br>অন্যান্য Others ....X<br>নির্দিষ্ট করুন (specify)________________________<br>জানি না/ মনে নাই Don't know/Can't remember ....Z			
w177.	শেষ গর্ভের ডেলিভারীর পর, কোন স্বাস্থ্যকর্মী স্বাস্থ্যকেন্দ্রে বা বাড়ীতে বা অন্য কোন স্থানে আপনার স্বাস্থ্য পরীক্ষা করেছিলেন কি (৪২ দিন পর্যন্ত)?<br>Did a health care provider check on your health after the delivery of your recent pregnancy, either at a health facility, home or other location (up to 42 days)?	হ্যাঁ Yes .... 1<br>→কতবার How many times: |____||____| না No .... 2<br>জানি না/ মনে নাই Don't know/Can't remember 9			**2/9 → w 183**
	**w177 থেকে পূরুন করুন**	**1st visit**	**2nd Visit**	**3rd visit**	
w178.	আপনার শেষ গর্ভের ডেলিভারীর পর কোন মেডিকেল চেক-আপ (PNC) পেয়েছিলেন কি?<br>Did you receive PNC?	হ্যাঁ Yes = 1<br>না No = 2<br>জানি না/ মনে নাই<br>DK/DR = 9	হ্যাঁ Yes = 1<br>না No = 2<br>জানি না/ মনে নাই<br>DK/DR = 9	হ্যাঁ Yes = 1<br>না No = 2<br>জানি না/ মনে নাই<br>DK/DR = 9	**2/9 → w 183**
w179.	ডেলিভারীর কত সময় পর আপনার মেডিকেল চেক-আপ (PNC) করানো হয়েছিল?<br>*[যদি এক ঘন্টার কম হয়, তাহলে ০০ ঘন্টা রেকর্ড করুন]*<br>How long after the delivery did the health contact take place? [If less than 1 hour record 00]	|____|____| ঘন্টা/দিন<br>Hours/Days	|____|____| দিন<br>Days	|____|____| দিন<br>Days	
w180.	কে মেডিকেল চেক-আপ (PNC) করেছিলেন?<br>(কোড লিস্ট থেকে কোড লিখুন)<br>Who provided you PNC?<br>(Fill out from the codebook provided)	ব্যাক্তির কোড<br>Person Code:<br>|____|____|	ব্যাক্তির কোড<br>Person Code:<br>|____|____|	ব্যাক্তির কোড<br>Person Code:<br>|____|____|	
w181.	কোথায় মেডিকেল চেক-আপ (PNC) করা হয়েছিল?<br>(কোড লিস্ট থেকে কোড লিখুন)<br>From where (place) did you attend PNC?<br>(Fill out from the codebook provided)	স্থানের কোড<br>Place Code:<br>|____|____|	স্থানের কোড<br>Place Code:<br>|____|____|	স্থানের কোড<br>Place Code:<br>|____|____|	
w182.	I182a.<br>আপনার মেডিকেল চেক-আপ (PNC) এর সময় আপনার স্বামী আপনার সাথে গিয়েছিলেন কি?<br>Did your husband travel with you?	হ্যাঁ Yes = 1<br>না No = 2<br>জানি না/ মনে নাই<br>DK/DR = 9	হ্যাঁ Yes = 1<br>না No = 2<br>জানি না/ মনে নাই<br>DK/DR = 9	হ্যাঁ Yes = 1<br>না No = 2<br>জানি না/ মনে নাই<br>DK/DR = 9	
	I182b.<br>আপনার মেডিকেল চেক-আপ (PNC) এর সময় আপনার স্বামী আপনার সাথে উপস্থিত ছিলেন কি?<br>Was your husband present with you during PNC contact?	হ্যাঁ Yes = 1<br>না No = 2<br>জানি না/ মনে নাই<br>DK/DR = 9	হ্যাঁ Yes = 1<br>না No = 2<br>জানি না/ মনে নাই<br>DK/DR = 9	হ্যাঁ Yes = 1<br>না No = 2<br>জানি না/ মনে নাই<br>DK/DR = 9	

NO	Questions And Filters	Coding Categories	Skip
w183.	প্রসবপরবর্তী সময়ে আপনার স্বামীর নিকট থেকে আপনি কতটা সহযোগিতা পেয়েছিলেন?<br>After childbirth how much support did you receive from your husband?	পর্যাপ্ত Sufficient .... 1<br>মোটামুটি Neither sufficient nor insufficient .... 2<br>অপর্যাপ্ত Insufficient 3	
w184.	প্রসবপরবর্তী সময়ে আপনার নিজের যত্ন নিতে আপনার পরিবারের সদস্যরা আপনাকে কতটা সহায়তা করেছিল?<br>After childbirth how much support did you receive from your family members?	পর্যাপ্ত Sufficient .... 1<br>মোটামুটি Neither sufficient nor insufficient .... 2<br>অপর্যাপ্ত Insufficient 3	

Section B: Newborn care

নবজাতকের যত্ন

১ লা জানুয়ারী, ২০১৭ থেকে ৩১ অক্টোবার ২০১৭ এর মধ্যে গর্ভ ফলাফল হয়েছে এমন মহিলাকে এই প্রশ্নগুলো করতে হবে।

All questions are to be addressed to woman with pregnancy outcome from 01 January 2017 to 31 October 2017.

	Questions and Filters	Coding Categories	Skip
w185.	w185a.<br>**১ লা জানুয়ারী, ২০১৭ থেকে ৩১ অক্টোবার ২০১৭ এর মধ্যে** গর্ভের ফলাফল কি ছিল?<br>What was the outcome of your pregnancy from 01 January 2017 to 31 October 2017?	জীবিত জন্ম Live Birth ....1<br>মৃত জন্ম Still Birth (>28 week)....................2<br>গর্ভপাত Abortion (<28 week) 3	2/3→ w212
	w185.b.<br>শেষ গর্ভধারনের সিদ্ধান্ত কে নিয়েছিল?<br>Who took the decision about your last conception?	আমি নিজে Me ....1<br>আমার স্বামী My husband ....2<br>উভয়ে Both of us....................3<br>অন্য কেউ (উল্লেখ করুন)<br>Others (Please mention)__________________...............7<br>জানা নাই/মনে নাই Don't know/can't remember 9	
w186.	w186a.<br>১ লা জানুয়ারী, ২০১৭ থেকে ৩১ অক্টোবার ২০১৭ এর মধ্যে জন্ম হয়েছে এমন শিশুর নাম, লিঙ্গ এবং তার জন্ম তারিখ লিখুন?<br>What is the date of the live birth that you had from 01 January 2017 to 31 October 2017? What is the name and sex of the child?<br>নাম Name ___________________________________	জন্ম তারিখ |____|____|/|____|____|/|____|____| Date of birth দিন মাস বছর<br>ছেলে Male .... 1<br>মেয়ে Female .... 2<br>অন্যান্য others_________________________....................3	
	w186b.<br>গর্ভের ক্রমানুসারে আপনার শেষ গর্ভটি কততম গর্ভ ছিল?<br>What was the order of your last pregnancy?	|____|____| তম	
	w186c.<br>শিশুর বর্তমান অবস্থা<br>Present status of the child	জীবিত Live .... 1<br>মৃত Dead.................... 2<br>মৃত হলে, মৃত্যুর তারিখঃ |____|____|/|____|____|/|____|____|	
w187.	নাড়ি কাটার আগে বা পরে (নাম)-এর নাড়িতে কোন কিছু দেয়া হয়েছিল কি?<br>Was anything applied on the umbilical cord of <Name> either before or after it was cut?	হ্যাঁ (Yes) ....1<br>না (No) ....2<br>জানা নাই/মনে নাই DK/can't remember....................9	2/9→ w189
w188.	নাড়ি কাটার পর (নাম)-এর নাড়িতে কি দেয়া হয়েছিল?<br>[জিজ্ঞেস করুন, আরও কিছু? [উত্তরদাতার নিজে থেকে দেয়া সবগুলো উত্তরই বৃত্তায়িত করুন । উত্তরগুলো পড়ে শুনাবেন না। একাধিক উত্তর হতে পারে।]<br>What was applied on the cut cord?<br>[Do not read out the options. Keep asking what else. Record all the answers.]	গোবর Cowdung.................... A<br>যেকোন ধরনের তেল Any kind of oil.................... B<br>অ্যান্টিসেপটিক (ডেটল/স্যাভলন/হেক্সিসল)<br>Antiseptic (Detol/savlon/hexisol) .... C<br>ছাই Ash.................... D<br>অ্যান্টিবায়োটিক (পাউডার/মলম) Antibiotics<br>(Powder / Ointment)....................E<br>চিবানো চাল Chewed rice....................F<br>হলুদের রস/গুড়া Turmeric juice/powder .... G<br>আদার রস Ginger juice.................... H<br>সিঁদুর Shidur ....I<br>বরিক পাউডার Boric powder ....J<br>জেনসিয়ান ভায়োলেট/নীল কালি Gentian violet/Blue ink .... K<br>ট্যালকম পাউডার Talcom Powder....................L<br>চুলার পোড়া মাটি Dust of earth-burner ....M<br>অন্যান্য Other.................... X<br>নির্দিষ্ট করুন (specify)___________________________<br>জানা নাই/মনে নাই Don't know/can't remember .... Z	
w189.	(নাম)-এর নাড়িতে কখনও ক্লোরহেক্সিডিন দেয়া হয়েছিল কি?<br>(বোতলটি দেখান)<br>Was chlorhexidine applied to the stump at any time?<br>(Show the bottle)	হ্যাঁ (Yes) ....1<br>না (No) ....2<br>জানা নাই/মনে নাই DK/can't remember....................9	**2/9→** w192
w190.	(নাম)-এর নাড়ি কাটার কতক্ষন পর প্রথম ক্লোরহেক্সিডিন দেয়া হয়েছিল?<br>How long after the cord was cut was chlorhexidine first applied?	মিনিট Minutes.................... 1 |____|____| ঘন্টা Hours .... 2 |____|____| জানি না Don't know/can't remember....................99	

	Questions and Filters	Coding Categories	Skip
w191.	কতদিন ক্লোরহেক্সিডিন দেয়া হয়েছিল?<br>For how many days was chlorhexidine applied to the stump?	১ দিন 1 Day ....1<br>২-৭ দিন 2-7 Days ....2<br>৭ দিনের বেশী More than 7 Days ....3<br>জানি না Don't know/can't remember.................................9	
w192.	(নাম) এর ডেলিভারীর পর কোন স্বাস্থ্যকর্মী বা কোন প্রশিক্ষনহীন দাই বাড়িতে, স্বাস্থ্যকেন্দ্রে বা অন্য কোন স্থানে (নাম) এর স্বাস্থ্য পরীক্ষা করেছিলেন কি? (৪২ দিন পর্যন্ত)<br>After (Name) was born, did any health care provider or traditional birth attendant check on (Name's) health at home, health facility or other place (up to 42 days)?	হ্যাঁ (Yes) ....1<br>না (No) ....2<br>জানা নাই/মনে নাই DK/can't remember.................................9	**2/9→**<br>w200
w193.	জন্মের কত সময় পর প্রথমবার (নাম) এর স্বাস্থ্য পরীক্ষা করানো হয়েছিল?<br>*[যদি এক দিনের কম হয়, তাহলে 1বৃত্তায়িত করে ঘন্টায় রেকর্ড করুন, যদি এক থেকে ছয় দিন হয়, তাহলে 2 বৃত্তায়িত করে দিনে রেকর্ড করুন, যদি ছয় দিনের বেশী হয়, তাহলে 3 বৃত্তায়িত করে সপ্তাহে রেকর্ড করুন]*<br>How many hours, days or weeks after the birth of (Name) did the first health contact take place?<br>If less than one day, circle 1 and record hours; if one to six days circle 2 and record days; if more than 6 days circle 3 and record weeks.	ঘন্টা Hours .... 1 |____|____| দিন Days... 2 |____|____| সপ্তাহ Weeks .... 3 |____|____| জানি না Don't know/can't remember...............................99	
w194.	ডেলিভারীর পর প্রথমবার কে (নাম) এর স্বাস্থ্য পরীক্ষা করেছিল?<br><u>(কোড লিস্ট থেকে কোড লিখুন)</u><br>Who checked on (Name's) first health check at that time? (Fill out from the codebook provoided)	ব্যাক্তির কোড Person Code:<br>|____|____|	
w195.	ডেলিভারীর পর প্রথমবার কোথায় (নাম) এর স্বাস্থ্য পরীক্ষা করিয়েছিলেন?<br><u>(কোড লিস্ট থেকে কোড লিখুন)</u><br>From where did you receive first health checkup for name? <u>(Fill out from the codebook provoided)</u>	স্থানের কোড Place Code:<br>|____|____|	
w196.	(নাম) এর ডেলিভারীর পর দ্বিতীয়বার (নাম) এর কোন স্বাস্থ্যকর্মী বা কোন প্রশিক্ষনহীন দাই দ্বিতীয়বার (নাম) এর স্বাস্থ্য পরীক্ষা করেছিলেন কি?<br>After (Name) was born, did any health care provider or traditional birth attendant check on (Name's) health second time?	হ্যাঁ (Yes) ....1<br>না (No) ....2<br>জানা নাই/মনে নাই DK/can't remember.................................9	2/9→<br>w200
w197.	জন্মের কত সময় পর দ্বিতীয়বার (নাম) এর স্বাস্থ্য পরীক্ষা করানো হয়েছিল?<br>*[যদি এক দিনের কম হয়, তাহলে 1বৃত্তায়িত করে ঘন্টায় রেকর্ড করুন, যদি এক থেকে ছয় দিন হয়, তাহলে 2 বৃত্তায়িত করে দিনে রেকর্ড করুন, যদি ছয় দিনের বেশী হয়, তাহলে 3 বৃত্তায়িত করে সপ্তাহে রেকর্ড করুন]*<br>How many hours, days or weeks after the birth of (Name) did the second health check take place?<br>[If less than one day, circle 1 and record hours; if one to six days circle 2 and record days; if more than 6 days circle 3 and record weeks.]	ঘন্টা Hours .... 1 |____|____| দিন Days... 2 |____|____| সপ্তাহ Weeks .... 3 |____|____| জানি না Don't know/can't remember...............................99	
w198.	ডেলিভারীর পর কে দ্বিতীয়বার (নাম) এর স্বাস্থ্য পরীক্ষা করেছিল?<br><u>(কোড লিস্ট থেকে কোড লিখুন)</u><br>Who checked on (Name's) health at that time?<br><u>(Fill out from the codebook provoided)</u>	ব্যাক্তির কোড Person Code:<br>|____|____|	
w199.	ডেলিভারীর পর দ্বিতীয়বার কোথায় (নাম) এর স্বাস্থ্য পরীক্ষা করিয়েছিলেন?<br><u>(কোড লিস্ট থেকে কোড লিখুন)</u><br>From where did you receive second health checkup for name? (Fill out from the codebook provoided)	স্থানের কোড Place Code:<br>|____|____|	
w200.	(নাম) এর জন্মের প্রথম দুই দিনের মধ্যে স্বাস্থ্যকর্মী নিম্মলিখিত কাজ গুলো করেছিল?<br>ক. নবজাতকের বিপদ চিহ্ন বিষয়ক পরামর্শ করা<br>খ. বুকের দুধ খাওয়ানো বিষয়ক পরামর্শ করা<br>During the first two days after (NAME)'s birth, did any health care provider do the following?<br>a. Counsel you on danger signs for newborns?<br>b. Counsel you on breastfedding?<br>**(DK = Don't know)**	| | **YES** | **NO** | **DK** | | নবজাতকের বিপদ চিহ্ন বিষয়ক পরামর্শ করা Counsel you on danger signs for newborns | 1 | 2 | 3 | | বুকের দুধ খাওয়ানো বিষয়ক পরামর্শ করা Counsel you on breastfeeding | 1 | 2 | 3 |	
w201.	নবজাতকের স্বাস্থ্যপরিচর্যার জন্য স্বামীর নিকট থেকে আপনি কতটা সহযোগিতা পেয়েছিলেন?<br>How much support did you recieve from your husband to take care of the newborn?	পর্যাপ্ত Sufficient ....1<br>মোটামুটি Neither sufficient nor insufficient ....2<br>অপর্যাপ্ত Insufficient 3	

	Questions and Filters	Coding Categories	Skip
w202.	নবজাতকের স্বাস্থ্যপরিচর্যার জন্য পরিবারের অন্যান্য সদস্যরা (স্বামী বাদে) আপনাকে সহায়তা করেছিলেন কি?<br>How much support did you recieve from other family members (except husband) to take care of the newborn?	পর্যাপ্ত Sufficient ....1<br>মোটামুটি Neither sufficient nor insufficient ....2<br>অপর্যাপ্ত Insufficient 3	

	Questions and Filters		Unprompted Yes	Prompted Yes	No	Skip
w203.	w203a.<br>কখনও কখনও নবজাতক বাচ্চা একমাস বয়সের মধ্যেই খুব অসুস্থ হয়ে যায় এবং তখন তাদেরকে অবিলম্বে চিকিৎসার জন্য হাসপাতালে/ ডাক্তারের কাছে নিয়ে যেতে হয়। কি ধরনের লক্ষণ দেখলে আপনি আপনার নবজাতককে সাথে সাথেই চিকিৎসার জন্য হাসপাতালে বা ডাক্তারের কাছে নিয়ে যাবেন?<br>[জিজ্ঞেস করুন, আরও কিছু? উত্তরদাতার নিজে থেকে দেয়া সবগুলো উত্তর প্রথমেই বৃত্তায়িত করুন । উত্তরগুলো পড়ে শুনাবেন না। একাধিক উত্তর হতে পারে। এরপর বাকি উত্তরগুলো পড়ে শুনান]<br><br>Sometimes newborns, within the first month of life, have severe illness and should be taken immediately to a health facility.<br>What types of symptoms would cause you to take your newborn to a health facility right away?<br>[Do not read out the options. Keep asking what else. Record all the answers first. Then read out the remaining options]	খাওয়া দাওয়া বন্ধ / হঠাৎ খাওয়া দাওয়ায় অরুচি<br>(Not feeding well) ....A	1	2	3	
		জ্বর fever ....B	1	2	3	
		মারাত্মক কম তাপমাত্রা/বগলের তাপমাত্রা ৯৬^{0}ফাঃ এর কম<br>(Low temperature/ Hypothermia)...... C	1	2	3	
		শ্বাস প্রশ্বাসের হার ১ মিনিটে ৬০ এর বেশী বা দ্রুত শ্বাস<br>(Very fast breathing) ....D	1	2	3	
		বুকের নিচের অংশ তীব্রভাবে ভিতরে ডেবে যাওয়া<br>(Severe chest in-drawing) .... E	1	2	3	
		নেতিয়ে পড়া / শুধুমাত্র উদ্দীপনায় নড়াচড়া করা<br>(Less movement than normal/ movement only when stimulate ....F	1	2	3	
		খিঁচুনী বা খিঁচুনীর ইতিহাস<br>(Convulsion or h/o convulsions ....G	1	2	3	
		নাভি থেকে পুঁজ বা দুর্গন্ধযুক্ত কিছু বের হওয়া/ লাল এবং ফুলে যাওয়া<br>(Pus/foul smelling discharge from umbilicus/<br>Red and swollen) ....H	1	2	3	
		অন্যান্য,<br>Others..X	নির্দিষ্ট করুন<br>Specify________________________________			
	w203b.<br>নবজাতকের অসুস্থতার এই লক্ষণ সম্পর্কে আপনি কার-কার কাছ থেকে জানতে পেরেছেন? (কোড লিস্ট থেকে কোড লিখুন)<br>From whom did you come to know about these special rights? (Fill out from the codebook)		ব্যক্তির কোড Person code<br>|___|___| |___|___| |___|___| |___|___| 1 2 3 4			
	w203c.<br>নবজাতকের অসুস্থতার এই লক্ষণ সম্পর্কে আপনি কোথা থেকে জানতে পেরেছেন? (কোড লিস্ট থেকে কোড লিখুন)<br>From where did you come to know about these special rights? (Fill out from the codebook)		স্থানের কোড Place Code:<br>|___|___| |___|___| |___|___| |___|___| 1 2 3 4			

	Questions and Filters		UY	PY	No	Skip
w204.	জন্মের প্রথম এক মাসের মধ্যে আপনার বাচ্চার কি কি সমস্যা হয়েছিল?<br>[উত্তরগুলো পড়ে শুনাবেন না। জিজ্ঞেস করুন, আরও কিছু? উত্তরদাতার নিজে থেকে দেয়া সবগুলো উত্তরই প্রথমেই বৃত্তায়িত করুন। এরপর বাকি উত্তরগুলো পড়ে শুনান]<br><br>Which kind of problems did your newborn suffer during the first one month?<br>[Do not read out the options. Keep asking what else. Record all the answers first. Then read out the remaining options]	খাওয়া দাওয়া বন্ধ / হঠাৎ খাওয়া দাওয়ায় অরুচি<br>(Not feeding well) ....A	1	2	3	All 3→ w212
		জ্বর fever ....B	1	2	3	
		মারাত্মক কম তাপমাত্রা/<br>বগলের তাপমাত্রা ৯৬^{0}ফাঃ এর কম<br>(Low temperature/ Hypothermia)--.........C	1	2	3	
		শ্বাস প্রশ্বাসের হার ১ মিনিটে ৬০ এর বেশী বা দ্রুত শ্বাস<br>(Very fast breathing) ....D	1	2	3	
		বুকের নিচের অংশ তীব্রভাবে ভিতরে ডেবে যাওয়া<br>(Severe chest in-drawing).........................E	1	2	3	

<table>
<tr><th></th><th>Questions and Filters</th><th colspan="4">Coding Categories</th><th>Skip</th></tr>
<tr><td></td><td></td><td>নেতিয়ে পড়া / শুধুমাত্র উদ্দীপনায় নড়াচড়া করা
(Less movement than normal/
movement only when stimulate .... F</td><td>1</td><td>2</td><td>3</td><td></td></tr>
<tr><td></td><td></td><td>খিঁচুনী বা খিচুনীর ইতিহাস
(Convulsion or h/o convulsions................G</td><td>1</td><td>2</td><td>3</td><td></td></tr>
<tr><td></td><td></td><td>নাভী থেকে পুঁজ বা দুর্গন্ধযুক্ত কিছু বের হওয়া/ লাল এবং ফুলে যাওয়া
(Pus/foul smelling discharge
from umbilicus/
Red and swollen) ....H</td><td>1</td><td>2</td><td>3</td><td></td></tr>
<tr><td></td><td></td><td>অন্যান্য, নির্দিষ্ট করুন
Others X
Specify_____________________________</td><td colspan="3"></td><td></td></tr>
<tr><td></td><td>একাধিক সমস্যার ক্ষেত্রে মায়ের দৃষ্টিতে সবচেয়ে মারাত্মক সমস্যার জন্য নিম্নের প্রশ্নগুলো প্রযোজ্য
Incase of multiple illness episode please consider the most complicated episode as perceived by the mother</td><td colspan="4"></td><td></td></tr>
<tr><td>w205.</td><td>বাচ্চার এই সমস্যা/জটিলতার জন্য আপনি কি কাউকে দেখিয়েছিলেন বা কারও সাহায্য নিয়েছিলেন?
Did you seek any sort of treatment for this problem/complication?</td><td colspan="4">হ্যাঁ (Yes) 1
না (No) 2
জানা নাই/মনে নাই DK/can't remember.................................9</td><td>2/9→
w212</td></tr>
<tr><td>w206.</td><td>এই সমস্যা/জটিলতার জন্য আপনি কাকে কাকে দেখিয়েছিলেন বা কার কার সাহায্য নিয়েছিলেন?
(কোড লিস্ট থেকে কোড লিখুন)
From whom did you seek treatment for this problem/complication?
(Fill out from the codebook provided)</td><td colspan="4">ব্যক্তির কোড Person code
|____||____| |____||____| |____||____| |____||____|
1 2 3 4</td><td></td></tr>
<tr><td>w207.</td><td>এই সমস্যা/জটিলতার জন্য আপনি কোথায় দেখিয়েছিলেন বা কোথায় সেবা পেয়েছিলেন?
(কোড লিস্ট থেকে কোড লিখুন)
Where did you go to seek care for this problem/complication?
(Fill out from the codebook provided)</td><td colspan="4">স্থানের কোড Place code
|____|____| |____|____| |____|____| |____|____|
1 2 3 4</td><td></td></tr>
<tr><td>w208.</td><td>এই সমস্যা বা জটিলতার জন্য সেবা গ্রহনের সিদ্ধান্ত নেওয়ার ক্ষেত্রে কে কে জড়িত ছিলেন?
(সব সম্পর্কগুলো নবজাতকের মায়ের দিক থেকে বিবেচিত)
Who was involved in taking decision regarding seeking care for this problem?
[All relations are in respect to the mother of the newborn]</td><td colspan="4">মহিলা নিজে Self..A
স্বামী Husband..B
শ্বাশুড়ী Mother in law C
শ্বশুর Father in law..D
বাবা Father .... E
মা Mother .... F
ননদ Sister in law ....G
দেবর Brother in law ....H
ভাসুর Brother in law... I
ভাই Brother .... J
বোন Sister ....K
অন্যান্য আত্নীয় Other relatives L
প্রতিবেশি Neighbours .... M
কমিউনিটি স্বাস্থ্যসেবা প্রদানকারী
Community health care provider....................................N
অন্য কেউ Others________________________....................X</td><td></td></tr>
<tr><td>w209.</td><td>এই সমস্যা বা জটিলতার জন্য সেবা গ্রহনের সিদ্ধান্ত নেওয়ার ক্ষেত্রে কার সিদ্ধান্ত চূড়ান্ত বলে গন্য করা হয়েছিল?
(সব সম্পর্কগুলো নবজাতকের মায়ের দিক থেকে বিবেচিত)
Who had the final say in taking decision regarding seeking care for this problem?
[All relations are in respect to the mother of the newborn]</td><td colspan="4">ব্যক্তির কোড Person Code:
|____|____|</td><td></td></tr>
<tr><td>w210.</td><td>এই সমস্যা বা জটিলতার জন্য সেবা গ্রহনের সিদ্ধান্ত নেওয়ার ক্ষেত্রে আপনি কি আপনার মতামত স্বাধীনভাবে প্রকাশ করতে পেরেছিলেন?
Were you allowed to share your opinion freely in taking decision regarding seeking care for this problem?</td><td colspan="4">হ্যাঁ (Yes) 1
না (No) 2
জানা নাই/মনে নাই DK/can't remember.................................9</td><td></td></tr>
</table>

	Questions and Filters	Coding Categories	Skip
w211.	এই সমস্যা বা জটিলতার জন্য সেবা গ্রহনের সিদ্ধান্ত নেওয়ার ক্ষেত্রে আপনার মতামত কতটুকু গুরুত্বপূর্ন ছিল?<br>How important was your opinion in taking decision regarding seeking care for this problem?	গুরুত্বপূর্ন Important ....1<br>মোটামুটি গুরুত্বপূর্ন Somewhat important................................2<br>গুরুত্বপূর্ন নয় Not important..3<br>জানা নাই/মনে নাই DK/can't remember..................................9	

Section C: Women's empowerment and health seeking behaviour
নারীর ক্ষমতায়ন এবং স্বাস্থ্যসেবা গ্রহণের আচরণ

	Questions and Filters	Coding Categories	Skip
w212.	আপনি কোন সালের কোন মাসে জন্ম গ্রহন করেছিলেন?<br>In what month and year were you born?<br>মাস জানা না *না থাকলে "99" লিখুন*<br>(If don't know month, write '99')	মাস (Month) ....|____|____| সাল (Year)..................................... |____|____||____|____|	
w213.	বর্তমানে আপনার বয়স কত?<br>How old were you at your last birthday?	বয়স (পূর্ন বছরে) Age in completed years .|____|____|	
w214.	আপনি কি কখনও স্কুলে, মাদ্রাসায় বা উপানুষ্ঠানিক শিক্ষা স্কুলে (বয়স্ক শিক্ষা কেন্দ্র বা অন্যকোথাও) লেখাপড়া করেছেন?<br>Did you ever study in school, madrasa or non formal school?	হ্যাঁ (Yes) ....1<br>না (No) 2	2→ w216
w215.	আপনি সর্বোচ্চ কোন ক্লাস/শ্রেণী পর্যন্ত লেখাপড়া করেছেন?<br>What is the highest grade/class or number of years of studies you have completed at that schooling?	সর্বোচ্চ কোন ক্লাস Highest Class/Grade ....|_____|_____|	
w216.	গত ৭ দিনে আপনি বাড়ির কাজ ব্যতিত অন্য কোন কাজ করেছেন কি?<br>Aside from your own housework, have you done any work in the last seven days?	হ্যাঁ (Yes)...1<br>না (No) 2	
w217.	আপনি জেনে থাকবেন, কিছু নারীরা পারিশ্রমিকের বিনিময়ে কাজ করেন, কেউ জিনিসপত্র বিক্রি করেন, কেউ ক্ষুদ্র ব্যবসা, অথবা পারিবারিক খামার এবং পারিবারিক ব্যবসার মাধ্যমে অর্থ উপার্জন করে থাকেন। এগুলোর মধ্যে গত ৭ দিনে আপনি কোন কাজ করেছেন কি?<br>As you know, some women take up jobs for which they are paid in cash or kind. Others sell things, have a small business or work on the family farm or in the family business.<br>In the last seven days, have you done any of these things or any other work?	হ্যাঁ (Yes)...1<br>না (No) 2	1→ w219
w218.	যদি আপনি গত ৭ দিনে কোন কাজ না করে থাকেন, তবে কি আপনার অন্য কোন চাকরী বা ব্যবসা আছে যা থেকে আপনি অসুস্থতা, অবকাশ, মাতৃত্বকালীন অথবা অন্য কোন কারনে অনুপস্থিত আছেন?<br>Although you did not work in the last seven days, do you have any job or business from which you were absent for leave, illness, vacation, maternity leave, or any other such reason?	হ্যাঁ (Yes)...1<br>না (No) 2	
w219.	গত ১২ মাসে আপনি কি কোন কাজ করেছেন যাতে অর্থ উপার্জন হয়?<br>Have you done any work in the last 12 months?	হ্যাঁ (Yes)...1<br>না (No) 2	**2**→w225
w220.	আপনার পেশা কি, মানে, আপনি প্রধানত কি ধরনের কাজ করে থাকেন?<br>What is your occupation, that is, what kind of work do you mainly do?		
w221.	আপনি কি এই কাজ আপনার পরিবারের কোন সদস্যের জন্য, অন্য কারও জন্য করে থাকেন অথবা আপনি কি স্বনিযুক্ত?<br>Do you do this work for a member of your family, for someone else, or are you self-employed?	পরিবারের সদস্যর জন্য For family member ....1<br>অন্য কারও জন্য For someone else ....2<br>স্বনিযুক্ত Self employed 3	
w222.	সাধারনত আপনি কি সারা বছরব্যাপী, মৌসুমে অথবা কদাচিৎ কাজ করে থাকেন?<br>Do you usually work throughout the year, or do you work seasonally, or only once in a while?	সারা বছরব্যাপী Throughout the year ....1<br>মৌসুমী Seasonanlly ....2<br>কদাচিৎ Once in a while 3	
w223.	আপনাকে কি এই কাজের বিনিময়ে নগদ অর্থ বা অন্য কিছু দেওয়া হয় নাকি আদৌ কিছু দেওয়া হয় না?<br>Are you paid in cash or kind for this work or are you not paid at all?	নগদ অর্থ Cash only ....1<br>নগদ অর্থ এবং অন্য কিছু Cash and kind ....2<br>শুধু অন্য কিছু In kind only ....3<br>কিছু দেওয়া হয় না Not paid...4	**4**→ w225
w224.	উপার্জিত অর্থ কিভাবে ব্যবহৃত হবে এই বিষয়ে সাধারনত কে সিদ্ধান্ত নেয়: আপনি, আপনার স্বামী, আপনি এবং আপনার স্বামী যৌথ ভাবে অথবা অন্য কেউ?<br>Who usually decides how the money you earn will be used: you, your husband, you and your husband jointly, or someone else?	আমি নিজে Me ....1<br>আমার স্বামী My husband ....2<br>উভয়ে Both of us..3<br>অন্য কেউ (উল্লেখ করুন)<br>Others (Please mention) _______________________......7<br>জানা নাই/মনে নাই Don't know/can't remember 9	
w225.	আপনার স্বাস্থ্যসেবা গ্রহনের ক্ষেত্রে কে সিদ্ধান্ত নেয়: আপনি, আপনার স্বামী, আপনি এবং আপনার স্বামী যৌথ ভাবে অথবা অন্য কেউ?<br>Who usually makes decisions about health care for yourself: you, your husband, you and your husband jointly, or someone else?	আমি নিজে Me ....1<br>আমার স্বামী My husband ....2<br>উভয়ে Both of us..3<br>অন্য কেউ (উল্লেখ করুন)<br>Others (Please mention) _______________________......7<br>জানা নাই/মনে নাই Don't know/can't remember 9	

w226.	বাড়ির গুরুত্বপূর্ন গৃহস্থালি জিনিসপত্র ক্রয়ের ক্ষেত্রে সাধারনত কে সিদ্ধান্ত নেয়?<br>Who usually makes decisions about making major household purchases?	আমি নিজে Me........1<br>আমার স্বামী My husband........2<br>উভয়ে Both of us........3<br>অন্য কেউ (উল্লেখ করুন)<br>Others (Please mention) ______......7<br>জানা নাই/মনে নাই Don't know/can't remember........9	
w227.	আপনার পরিবারের অথবা আত্নীয়স্বজনের সাথে দেখা করতে যাওয়ার ক্ষেত্রে সাধারনত কে সিদ্ধান্ত নেয়?<br>Who usually makes decisions about visits to your family or relatives?	আমি নিজে Me........1<br>আমার স্বামী My husband........2<br>উভয়ে Both of us........3<br>অন্য কেউ (উল্লেখ করুন)<br>Others (Please mention) ______......7<br>জানা নাই/মনে নাই Don't know/can't remember........9	
w228.	আপনার শিশুর স্বাস্থ্যের যত্ন নেওয়ার ক্ষেত্রে সাধারনত কে সিদ্ধান্ত নেয়?<br>Who usually makes decisions about your child health care?	আমি নিজে Me........1<br>আমার স্বামী My husband........2<br>উভয়ে Both of us........3<br>অন্য কেউ (উল্লেখ করুন)<br>Others (Please mention) ______......7<br>জানা নাই/মনে নাই Don't know/can't remember........9	
w229.	আপনি কি স্বাস্থ্যকেন্দ্রে বা হাসপাতালে একা যান (অথবা শুধু আপনার ছোট শিশু কে সাথে নিয়ে যান)?<br>Do you go to a health centre or hospital alone (or just with your young children)?	হ্যাঁ, একা Yes, Alone........1<br>হ্যাঁ, বাচ্চার সাথে Yes, with children........2<br>না No........3<br>অন্য কেউ (উল্লেখ করুন)<br>Others (Please mention) ______......7	
w230.	আপনি স্বাস্থ্যকেন্দ্রে বা হাসপাতালে একা যেতে পারবেন কি না (অথবা শুধু আপনার ছোট শিশু কে সাথে নিয়ে) সে সিদ্ধান্ত কে নেয়?<br>Who decides whethere you can go to a health centre or hospital alone (or just with your young children)?	আমি নিজে Me........1<br>আমার স্বামী My husband........2<br>উভয়ে Both of us........3<br>অন্য কেউ (উল্লেখ করুন)<br>Others (Please mention) ______......7<br>জানা নাই/মনে নাই Don't know/can't remember........9	
w231.	সাধারনত খানার/পরিবারের মধ্যে আপনার এবং আপনার নবজাতকের স্বাস্থ্য সম্পর্কিত কোন আলোচনা হয় কি?<br>In general, is there any discussion within the household regarding your health and the health of your newborn?	হ্যাঁ (Yes)........1<br>না (No)........2<br>জানা নাই/মনে নাই DK/can't remember........9	2/9→w236
w232.	সাধারনত খানার/পরিবারের মধ্যে আপনার এবং আপনার নবজাতকের স্বাস্থ্য সম্পর্কিত আলোচনায় কে কে অংশগ্রহন করে?<br>In general, who participates in these discussions regarding your health and the health of your newborn?	মহিলা নিজে Self........A<br>স্বামী Husband........B<br>শ্বাশুড়ী Mother in law........C<br>শ্বশুর Father in law........D<br>বাবা Father........E<br>মা Mother........F<br>ননদ Sister in law........G<br>দেবর Brother in law........H<br>ভাসুর Brother in law........I<br>ভাই Brother........J<br>বোন Sister........K<br>অন্যান্য আত্নীয় Other relatives........L<br>প্রতিবেশি Neighbours........M<br>কমিউনিটি স্বাস্থ্যসেবা প্রদানকারী Community health care provider........N<br>অন্য কেউ Others ______........X	

w233.	সাধারনত খানার/পরিবারের মধ্যে আপনার এবং আপনার নবজাতকের স্বাস্থ্য সম্পর্কিত আলোচনায় কার মতামত সবচেয়ে গুরুত্বপূর্ন?<br>In general, who has the final say in these discussions regarding your health and the health of your newborn?	মহিলা নিজে Self ....A<br>স্বামী Husband ....B<br>শ্বাশুড়ী Mother in law ....C<br>শ্বশুর Father in law ....D<br>বাবা Father ....E<br>মা Mother ....F<br>ননদ Sister in law ....G<br>দেবর Brother in law ....H<br>ভাসুর Brother in law ....I<br>ভাই Brother ....J<br>বোন Sister ....K<br>অন্যান্য আত্নীয় Other relatives ....L<br>প্রতিবেশি Neighbours ....M<br>কমিউনিটি স্বাস্থ্যসেবা প্রদানকারী Community health care provider ....N<br>অন্য কেউ Others__________________________.............X	
w234.	সাধারনত আপনার এবং আপনার নবজাতকের স্বাস্থ্য সম্পর্কিত এই আলোচনায় আপনাকে কি আপনার মতামত স্বাধীনভাবে প্রকাশ করার অনুমতি দেওয়া হয়?<br>In general, are you allowed to share your opinion freely in these discussions regarding your health and the health of your newborn?	হ্যাঁ Yes ....1<br>না No....................................2<br>প্রযোজ্য নয় 9	
w235.	সাধারনত আপনার এবং আপনার নবজাতকের স্বাস্থ্য সম্পর্কিত কোন সিদ্ধান্ত নেওয়ার ক্ষেত্রে আপনার মতামত কে কতটুকু মূল্যায়ন করা হয়?<br>In general, how much is your opinion valued in taking decision regarding your health and the health of your newborn?	খুব বেশি মূল্যায়ন করা হয়Valued very highly ....1<br>মোটামুটি মূল্যায়ন করা হয়Valued somewhat highly ....2<br>একেবারেই মূলায়ন করা হয় না Not valued at all....................3<br>জানা নাই/মনে নাই DK/can't remember....................9	
w236.	মাসিক সম্পর্কিত কোন তথ্যর প্রয়োজনে কোথায় যেতে হবে আপনি কি তা জানেন?<br>Do you know where to go if you needed information about menstrual periods?	হ্যাঁ (Yes)....................................1<br>না (No) ....2<br>জানা নাই/মনে নাই DK/can't remember....................9	
w237.	গর্ভ নিরোধক (জন্ম নিয়ন্ত্রন) পদ্ধতির প্রয়োজন হলে কোথায় যেতে হবে আপনি কি তা জানেন?<br>Do you know where to go if you needed contraception (birth control)?	হ্যাঁ (Yes)....................................1<br>না (No) ....2<br>জানা নাই/মনে নাই DK/can't remember....................9	
w238.	যৌন বাহিত সংক্রমনের চিকিৎসার প্রয়োজন হলে কোথায় যেতে হবে আপনি কি তা জানেন?<br>Do you know where to go if you needed treatment for a sexually transmitted infection?	হ্যাঁ (Yes)....................................1<br>না (No) ....2<br>জানা নাই/মনে নাই DK/can't remember....................9	
w239.	গর্ভ নিরোধক (জন্ম নিয়ন্ত্রন) পদ্ধতির প্রয়োজন হলে আপনি কি স্বাস্থ্য সেবাকেন্দ্রে বা ক্লিনিকে যেতে লজ্জা বা বিব্রতবোধ করেন?<br>Do you feel too shy or embarrassed to go to a clinic or center if you needed contraception (birth control)?	হ্যাঁ (Yes)....................................1<br>না (No) ....2<br>জানা নাই/মনে নাই DK/can't remember....................9	

Section D: Opinions regarding community participation

সামাজিক অংশগ্রহণ সম্পর্কিত মতামত

১ লা জানুয়ারী, ২০১৭ থেকে ৩১ অক্টোবার ২০১৭ এর মধ্যে গর্ভ ফলাফল হয়েছে এমন মহিলাকে এই প্রশ্নগুলো করতে হবে।

All questions are to be addressed to women with a delivery outcome from 01 January 2017 to 31 October 2017.

	Questions And Filters	Coding Categories	Skip
w240.	কমিউনিটি বা আপনার এলাকার মানুষ গর্ভবতী মহিলা এবং নবজাতকের সাহায্যের সাথে জড়িত- এমন কোন কার্যক্রমের ব্যাপারে অবগত ছিলেন কি?<br>Are you aware of any actions within the community to help pregnant women and newborns?	হ্যাঁ Yes ......1<br>না No ......2<br>জানি না/ মনে নাই Don't know/Can't remember 9	2/9 → W 244

	Questions And Filters		Unprompted Yes	Prompted Yes	No
w241.	কোন কার্যক্রমের ব্যাপারে অবগত ছিলেন?<br>[উত্তরগুলো পড়ে শুনাবেন না। জিজ্ঞেস করুন, আরও কিছু? উত্তরদাতার নিজে থেকে দেয়া সবগুলো উত্তরই প্রথমেই বৃত্তায়িত করুন। এরপর বাকি উত্তরগুলো পড়ে শুনান]<br>Which action(s) were you aware of?<br>[Do not read out the options. Keep asking what else. Record all the answers first. Then read out the remaining options]	মাতৃ ও শিশু স্বাস্থ্য বিষয়ে কমিউনিটিকে অবগত করা/শিক্ষা প্রদান<br>Community education/sensitization on MNH issues ...... A	1	2	3
		মাতৃ ও শিশু স্বাস্থ্য সেবা গ্রহনের জন্য কমিউনিটি কর্তৃক যানবাহনের ব্যবস্থা করা<br>Community organized transportation to reach MNH services ......B	1	2	3
		নারীদেরকে মাতৃ ও শিশু স্বাস্থ্য সেবা গ্রহনের জন্য কমিউনিটি সংগঠনের তহবিল থেকে আর্থিক সহায়তা প্রদান<br>Community organization of funds to assist women to pay for MNH services C	1	2	3
		স্বাস্থ্য সেবা সম্পর্কে পরিকল্পনা/মতামত প্রদানে কমিউনিটির সম্পৃক্ততা<br>Community involvement in planning/providing feedback to health services ...... D	1	2	3
		অন্যান্য, নির্দিষ্ট করুন<br>Other, specify ____________ X			

	Questions And Filters	Coding Categories	Skip
w242.	আপনি কি এই কার্যক্রম থেকে কোন সুবিধা পেয়েছিলেন?<br>Did you benefit from this/these actions?	হ্যাঁ Yes ......1<br>না No ......2<br>জানি না/ মনে নাই Don't know/Can't remember 9	2/9 → w 244
w243.	কোনটি থেকে<br>From which one(s)	মাতৃ ও শিশু স্বাস্থ্য বিষয়ে কমিউনিটিকে অবগত করা/শিক্ষা প্রদান<br>Community education/sensitization on MNH issues ......A<br>মাতৃ ও শিশু স্বাস্থ্য সেবা গ্রহনের জন্য কমিউনিটি কর্তৃক যানবাহনের ব্যবস্থা করা<br>Community organized transportation to reach MNH services ......B<br>নারীদেরকে মাতৃ ও শিশু স্বাস্থ্য সেবা গ্রহনের জন্য কমিউনিটি সংগঠনের তহবিল থেকে আর্থিক সহায়তা প্রদান Community organization of funds to assist women to pay for MNH services ......C<br>স্বাস্থ্য সেবা সম্পর্কে পরিকল্পনা/মতামত প্রদানে কমিউনিটির সম্পৃক্ততা<br>Community involvement in planning/providing feedback to health services ......D<br>অন্যান্য, নির্দিষ্ট করুন<br>Other, specify ____________ ......X<br>জানিনা / মনে নাই<br>Don't know/can't remember ...... Z	
	নিম্নের কথা গুলো সম্পর্কে আপনার মতামত কি?<br>What is your opinion regarding the following statements?		
w244.	খানা/ পরিবারে মা ও নবজাতকের স্বাস্থ্যের ব্যপারে আপনার এলাকার দম্পতিরা নিজেদের মধ্যে খোলামেলা আলোচনা করেন<br>Couples in your community discuss openly about the health of mothers and newborns in the household	সম্পূর্ণ একমত Strognly agree ......1<br>মোটামুটি একমত Somewhat aree ......2<br>একেবারেই একমত নয় Do not agree at all ......3<br>জানা নাই/মনে নাই DK/can't remember 9	
w245.	মা ও নবজাতকের স্বাস্থ্যে সম্পর্কে আপনার এলাকার নারীদের মতামত কে গুরুত্ব দেওয়া হয়<br>Women's opinions are considered important regarding MNH in your community	সম্পূর্ণ একমত Strognly agree ......1<br>মোটামুটি একমত Somewhat aree ......2<br>একেবারেই একমত নয় Do not agree at all ......3<br>জানা নাই/মনে নাই DK/can't remember 9	

	Questions And Filters	Coding Categories	Skip
w246.	আপনার এলাকার জনগন মা ও নবজাতকের স্বাস্থ্য সম্পর্কে সচেতন<br>People in your community are concerned about the health of mothers and newborns	সম্পূর্ণ একমত Strognly agree ....1<br>মোটামুটি একমত Somewhat aree ....2<br>একেবারেই একমত নয় Do not agree at all ....3<br>জানা নাই/মনে নাই DK/can't remember.................................9	
w247.	আপনার এলাকার জনগন মা ও নবজাতকের স্বাস্থ্য সম্পর্কে খোলামেলা আলোচনা করেন<br>People in your community talk openly about the health of women and newborns	সম্পূর্ণ একমত Strognly agree ....1<br>মোটামুটি একমত Somewhat aree ....2<br>একেবারেই একমত নয় Do not agree at all ....3<br>জানা নাই/মনে নাই DK/can't remember.................................9	
w248.	আপনার এলাকার জনগন মা ও নবজাতকের স্বাস্থ্যের উন্নয়নে একসাথে কাজ করেন<br>People in your community work together to help improve the health of mothers and newborns	সম্পূর্ণ একমত Strognly agree ....1<br>মোটামুটি একমত Somewhat aree ....2<br>একেবারেই একমত নয় Do not agree at all ....3<br>জানা নাই/মনে নাই DK/can't remember.................................9	
w249.	মা ও নবজাতকের স্বাস্থ্য সম্পর্কিত আপনার কোন উদ্বেগ/সমস্যা থাকলে এমন প্লাটফর্ম/স্থান আছে যেখানে আপনি এলাকার জনপ্রতিনিধিদের সাথে এ বিষয়ে আলোচনা করতে পারেন<br>If you have a concern related to the health of mothers and newborns, there is a platform/place where you can discuss this with community leaders	সম্পূর্ণ একমত Strognly agree ....1<br>মোটামুটি একমত Somewhat aree ....2<br>একেবারেই একমত নয় Do not agree at all ....3<br>জানা নাই/মনে নাই DK/can't remember.................................9	3/9 → W 251
w250.	যদি কোন প্লাটফর্ম / স্থান থাকে তার নাম-<br>If there is a platform, name of the platform is -		
w251.	মা ও নবজাতকের স্বাস্থ্য সম্পর্কিত আপনার কোন উদ্বেগ/সমস্যা থাকলে স্বাস্থ্য সেবাদানকারী প্রতিষ্ঠান কে আপনার সমস্যা অবহিত করার/ জানানোর একটি উপায়/পন্থা আছে<br>If you have a concern about the quality of health services for mothers and newborns, there is a way for you to share this with the health services	সম্পূর্ণ একমত Strognly agree ....1<br>মোটামুটি একমত Somewhat aree ....2<br>একেবারেই একমত নয় Do not agree at all ....3<br>জানা নাই/মনে নাই DK/can't remember.................................9	
w252.	যদি আপনি মা ও নবজাতকের স্বাস্থ্য সম্পর্কিত সমস্যা জানাতে পারেন সেক্ষেত্রে একটি সমাধান পাবার সম্ভবনা আছে<br>If you share your concerns about the health of mothers and newborns, it is likely that a solution will be found.	সম্পূর্ণ একমত Strognly agree ....1<br>মোটামুটি একমত Somewhat aree ....2<br>একেবারেই একমত নয় Do not agree at all ....3<br>জানা নাই/মনে নাই DK/can't remember.................................9	
w253.	**আপনার এলাকার মা ও নবজাতকের স্বাস্থ্যের উন্নয়ন সম্পর্কিত সমস্যা সমাধানে সিজি/সিএসজি গ্রুপের সদস্যরা সক্রিয়**<br>**The CGs/CSGs are active in solving problems to improve the health of mothers and newborns in your community.**	**সম্পূর্ণ একমত Strognly agree ....1**<br>**মোটামুটি একমত Somewhat aree ....2**<br>**একেবারেই একমত নয় Do not agree at all ....3**<br>**জানা নাই/মনে নাই DK/can't remember.................................9**	
w254.	**এলাকা থেকে স্বাস্থ্য সেবাদানকারী প্রতিষ্ঠানে তথ্য পৌছানোর ক্ষেত্রে সিজি/সিএসজি গ্রুপের সদস্যরা সক্রিয়**<br>**The CGs/CSGs are active in bringing information from the community back to the health services**	**সম্পূর্ণ একমত Strognly agree ....1**<br>**মোটামুটি একমত Somewhat aree ....2**<br>**একেবারেই একমত নয় Do not agree at all ....3**<br>**জানা নাই/মনে নাই DK/can't remember.................................9**	
w255.	**স্বাস্থ্য সেবাদানকারী প্রতিষ্ঠান থেকে এলাকায় তথ্য পৌছানোর ক্ষেত্রে সিজি/সিএসজি রা সক্রিয়**<br>**The CGs/CSGs are active in bringing information from the health services to the community**	**সম্পূর্ণ একমত Strognly agree ....1**<br>**মোটামুটি একমত Somewhat aree ....2**<br>**একেবারেই একমত নয় Do not agree at all ....3**<br>**জানা নাই/মনে নাই DK/can't remember.................................9**	
w256.	**আপনার এলাকায় সিজি/সিএসজি গ্রুপের সদস্যরা গুরুত্বপূর্ন**<br>**The CGs/CSGs are important in your community**	**সম্পূর্ণ একমত Strognly agree ....1**<br>**মোটামুটি একমত Somewhat aree ....2**<br>**একেবারেই একমত নয় Do not agree at all ....3**<br>**জানা নাই/মনে নাই DK/can't remember.................................9**	
w257.	স্বাস্থ্যসেবাপ্রদানকারীরা স্বাস্থ্যসেবা প্রদান করার ক্ষেত্রে এলাকার পছন্দকে গুরুত্ব দেয়<br>Health care providers are concerned about making sure that health services respond to community preferences?	সম্পূর্ণ একমত Strognly agree ....1<br>মোটামুটি একমত Somewhat aree ....2<br>একেবারেই একমত নয় Do not agree at all ....3<br>জানা নাই/মনে নাই DK/can't remember.................................9	

Section E: Household Information

খানা সম্পর্কিত তথ্য

This section contains some house hold information of the selected woman and other members of the same household

	**খানার গঠন (বর্তমানে জীবিত ও গৃহের নিয়মিত বাসিন্দা):**	**পুরুষ (**Male)		**মহিলা (**Female)	
	খানার মোট সদস্য সংখ্যা ________________জন	|____|____|	………..…	|____|____|	
A01	2 বছরের কম বয়সী শিশুর সংখ্যা<br>Number of U2 years children in the Household	|____|____|	………..…	|____|____|	
A02	2 - 5 বছরের কম বয়সী শিশুর সংখ্যা<br>Number of children of age 2-up to 5 years	|____|____|	………..…	|____|____|	
A03	5-15 বছরের কম বয়সী বালক / বালিকার সংখ্যা<br>Number of children/adolescents of age 5-up to 15 years	|____|____|	………..…	|____|____|	
A04	পরিবারে 15- 49 বছর বয়স্ক সদস্য সংখ্যা<br>Number of adults of age 15-49 years	|____|____|	………..…	|____|____|	
A05	15- 49 বছর বয়স্ক কখনও বিয়ে হয়েছিল এমন মহিলার সংখ্যা<br>Number of ever-married women of age 15-49 yrs		………..…	|____|____|	
A06	50 বছর বা তার উর্ধ্বে বয়সের সদস্য সংখ্যা<br>Number of adults of age 50+ years	|____|____|	………..…	|____|____|	

Section F: Socio-Economic Information
আর্থ-সামাজিক তথ্য

	Questions and Filters	Coding Categories	Skip
w258.	আপনার ঘরের/খানার সদস্যদের খাবার পানির প্রধান উৎস কি/কোথায়?<br><br>What is the main source of drinking water for members of your household?	**<u>পাইপের পানিঃ (Piped water)</u>**<br>বাড়ির ভিতরে ট্যাপের (পাইপের) পানি<br>(Piped inside dwelling) .... 11<br>বাড়ির বাহিরে ট্যাপের (পাইপের) পানি (Piped outside dwelling) 12<br>সরকারি ট্যাপের পানি Public Tap/Standpipe) .... 13<br>নলকুপের পানি(Tube well or borehole) .... 14<br>**<u>কুপের পানিঃ (Well water)</u>**<br>শ্যালো টিউবওয়েল (Shallow tubewell) .... 21<br>গভীর নলকূপ (Deep tubewell) .... 22<br>ছাউনি দেয়া কুপের পানি (Protected well)............................... 31<br>খোলা কুপের পানি (Unprotected well) .... 32<br>**<u>ঝর্নার পানি:(Water from Spring)</u>**<br>ছাউনি দেয়া ঝর্নার পানি (Protected spring) .... 41<br>খোলা ঝর্নার পানি(Unprotected spring) .... 42<br>বৃষ্টির পানি (Rain water) .... 51<br>ট্যাঙ্কার ট্রাক (Tanker Truck).. 61<br>ছোট ট্যাঙ্কের ঠেলা গাড়ি (Cart with small tank) .... 71<br>**<u>ভূ-পৃষ্ঠের পানিঃ (Surface water)</u>**<br>পুকুর/খাল/বদ্ধ জলাশয়/হ্রদ/দীঘি/বিল/হাওড়<br>(Pond/Tank/Lake) .... 72<br>নদী/বাধ/হ্রদ/পুকুর/ছরা/খাল/সেচ নাল<br>(River/Dam/ Lake/Pond/ Stream / Canal/Irrigation Channel)... 81<br>বোতলজাত পানি (BottledWater) .... 91<br>অন্যান্য (Other) _______________.................................. 97	
w259.	আপনাদের কি ধরনের পায়খানা/ল্যাট্রিন এর ব্যবস্থা আছে?<br>What kind of toilet facility does your household have?	**সেপটিক ট্যাংক/আধুনিক ল্যাট্রিন (Flush or Pour Flush Toilet)**<br>পৌরসভার লাইনে ফ্লাস করা<br>(Flush to piped sewer system) .... 11<br>সেপটিক ট্যাঙ্কে ফ্লাস করা (Flush to septic tank) .... 12<br>গর্তের(পিট) ল্যাট্রিনে ফ্লাস করা (Flush to pit latrine.................. 13<br>অন্য কোথাও ফ্লাস করা (Flush to somewhere else). .... 14<br>অন্য কোথাও ফ্লাস করা জানিনা<br>(Flush, don't know where... 15<br>**<u>গর্ত (পিট) টয়লেট/ল্যাট্রিন (Pit toilet/Latrine)</u>**<br>বায়ু চলাচলে উন্নত ব্যবস্থা<br>(Ventilated improved pit latrine)................................. 21<br>স্লাব দেয়া গর্তের(পিট) ল্যাট্রিন(Pit latrine with slab) .... 22<br>স্লাব ছাড়া গর্তের (পিট) ল্যাট্রিন<br>(Pit latrine without slab/ open pit)................................ 23<br>সার উৎপাদি ল্যাট্রিন (Composting latrine)............................ 31<br>বালতি টয়লেট(BucketToilet) .... 41<br>ঝুলন্ত ল্যাট্রিন (Hanging latrine).. 51<br>ল্যাট্রিন নাই/ঝোপ ঝাড়/মাঠ (No facility/Bush/Field).............. 52<br>অন্যান্য (Other) ______________________ 97	

	Questions and Filters	Coding Categories	Skip
w260.	আপনাদের ঘরের/খানার কোন সদস্যের এই জিনিসগুলো আছে কি?<br>[প্রত্যেকটি জিনিসের কথাই জিজ্ঞেস করুন]<br>How many of the followings are owned by your household?<br>[Ask about all listed]	**Yes No**<br>রেডিও (Radio)..1........ 2<br>টেলিভিশন (Television)..1........ 2<br>টেপ রেকর্ডার (Tape recorder) ....1........ 2<br>ভিসিপি/ভিসিআর/ভিডিভি (VCR/VCP/DVD player)........1........ 2<br>মোবাইল ফোন (Mobile phone) ....1........ 2<br>টেলিফোন (Land Telephone) ....1........ 2<br>বৈদ্যুতিক পাখা (Electric Fan)...................................1........ 2<br>কম্পিউটার (Computer) ....1........ 2<br>ফ্রিজ (Refrigerator) ....1........ 2<br>ঘড়ি (Watch)..1........ 2<br>আলমারি (Almira) ....1........ 2<br>রান্নাঘরের তাকিয়া (Kitchen cabinet) ....1........ 2<br>খাট/চৌকি (Cot/bed) ....1........ 2<br>টেবিল (Table) ....1........ 2<br>চেয়ার (Chair)...1........ 2<br>সোফা (Sofa) ....1........ 2<br>লেপ/কম্বল (Lep/Kombol/Blanket)............................1........ 2<br>তোষক/জাজিম (Toshok/Jajim/Matress) ....1........ 2<br>বাই সাইকেল (Bicycle) ....1........ 2<br>মোটর সাইকেল/স্কুটার (Motor cycle/Scooter) ....1........ 2<br>গরু/মহিষের গাড়ী (Animal drawn cart).......................1........ 2<br>গাড়ি/মাইক্রোবাস (Car/Microbus) ....1........ 2<br>মালবাহী গাড়ি/বাস (Truck/Bus)..................................1........ 2<br>ইঞ্জিন চালিত নৌকা (Motor-Boat) ....1........ 2<br>নৌকা (ইঞ্জিন ছাড়া) (Boat without motor) ....1........ 2<br>রিক্সা/ভ্যান (Rickshaw/Rickshaw Van)......................1........ 2<br>কার/ ট্রাক/ মোটর বাস (Car /truck/ motorbus) ....1........ 2<br>অটোবাইক/টেম্পু/সিএনজি (Autobike/Tempu/CNG) ......1........ 2	
w261.	আপনাদের ঘরে কি বিদ্যুৎ আছে?<br><br>Do you have Electricity in your house?	হ্যাঁ Yes.. 1<br>না No .... 2<br>প্রযোজ্য নয়.. 9	
w262.	আপনার ঘরে/খানায় সাধারনত কি ধরনের জ্বালানি ব্যবহার করা হয়?<br>What type of fuel does your household mainly use for cooking?	**Yes No**<br>বিদ্যুৎ (Electricity)..1........ 2<br>এলপি গ্যাস (LPG) ....1........ 2<br>প্রাকৃতিক গ্যাস (Natural Gas) ....1........ 2<br>বায়ো গ্যাস (Bio-Gas) ....1........ 2<br>কেরোসিন (Kerosene) ....1........ 2<br>কয়লা (Coal) ....1........ 2<br>চারকোল (Charcoal) ....1........ 2<br>কাঠ (Wood) ....1........ 2<br>খর/গুল্ম/ঘাস(Straw/Shrubs/Grass) ....1........ 2<br>ক্ষেতের ফসল (Agricultural Crop) ....1........ 2<br>গোবর (Animal dung) ....1........ 2<br>ঘরে কোন খাবার রান্না হয় না<br>(No food cooked in household) ....1........ 2<br><br>অন্যান্য (Other) ________________.........................97	

	Questions and Filters	Coding Categories	Skip
w263.	আপনারা যে ঘরে থাকেন সে ঘরের চালের/ছাদের প্রধান নির্মান-সামগ্রী কি?<br>[দেখে লিখুন, যদি থাকার জন্য একাধিক ঘর থাকে তবে সবচেয়ে বড় ঘরের নির্মান-সামগ্রী লিখুন]<br>Main Material of the Roof.<br>[Observe and write. If there are more than one house for living, write about the material of the roof of the biggest house]	প্রাকৃতিক ছাদঃ Natural Roof<br>ছাদ নেই No roof........ 11<br>খড়/ছন/তালপাতা Bamboo/Thatch/Palm leaf........ 12<br>কাঁচা ছাদঃ Rudimentary Roof<br>বাঁশ Bamboo........ 21<br>কাঠের তক্তা Wood Planks........ 22<br>কার্ডবোর্ড Cardbord........ 23<br>উন্নত ছাদঃ Finished roof (Pukka)<br>টিন Tin........ 31<br>সিমেন্ট Cement/Concrete........ 32<br>টালি Tiles........ 33<br>অন্যান্য (Other) ______________ 97	
w264.	আপনারা যে ঘরে থাকেন সে ঘরের মেঝের প্রধান নির্মাণ-সামগ্রী কি?<br>*[দেখে লিখুন, যদি থাকার জন্য একাধিক ঘর থাকে তবে সবচেয়ে বড় ঘরের নির্মান-সামগ্রী লিখুন]*<br>Main material of the Floor.<br>[Observe and write. If there are more than one house for living, write about the material of the roof of the biggest house]	কাঁচা মেঝে Natural floor<br>মাটি/বালু Earth/sand........ 11<br>প্রাথমিক পর্যায়ের মেঝেঃ Rudimentary floor<br>কাঠের তক্তা Wood Planks........ 21<br>তাল গাছ/ বাঁশ Palm/Bamboo........ 22<br>উন্নত মেঝে Finished floor<br>নকশা করা কাঠের পাটাতন/ পালিশকৃত কাঠ<br>Parquet/Polished Wood........ 31<br>সিমেন্ট/ইট বালি জমানো Cement/Concrete........ 32<br>সিরামিক টাইলস/মোজাইক Ceramic Tiles/Mosaic........ 33<br>অন্যান্য (Other) ______________........ 97	
w265.	আপনারা যে ঘরে থাকেন সে ঘরের দেয়ালের প্রধান নির্মান-সামগ্রী কি?<br>*[দেখে লিখুন, যদি থাকার জন্য একাধিক ঘর থাকে তবে সবচেয়ে বড় ঘরের নির্মান-সামগ্রী লিখুন]*<br>Main material of the Walls<br>[Observe and write. If there are more than one house for living, write about the material of the roof of the biggest house]	প্রাকৃতিক দেয়ালঃ Natural Walls<br>দেয়াল নাই No walls........ 11<br>পাটকাঠি/বেত/তাল গাছ/গাছের গুড়ি Cane/Palm/Trunks........ 12<br>মাটি Mud........ 13<br>প্রাথমিক পর্যায়ের দেয়ালঃ Rudimentary Walls<br>মাটিসহ বাঁশ Bamboo with Mud........ 21<br>মাটিসহ পাথর Stone with Mud........ 22<br>প্লাই-উড Plywood........ 23<br>কার্ডবোর্ড Cardbord........ 24<br>উন্নত দেয়ালঃ Finished Walls<br>টিন Tin........ 31<br>চুন সুড়কি দিয়ে তৈরী পাথরের দেয়াল Stone with lime........ 32<br>ইট (প্লাস্টার ছাড়া) Bricks (Without plaster)........ 33<br>ইট / সিমেন্ট (প্লাস্টার সহ) Bricks / Cement (With plaster)........ 34<br>উন্নতমানের কাঠের তক্তা Polished Wood Planks........ 35<br>অন্যান্য (Other) ______________........ 97	
w266.	আপনাদের খানায় কয়টি শোবার রুম আছে?<br>How many rooms are there in your house for household members to sleep in?	শোবার কক্ষের সংখ্যা Rooms........ |____|____|	
w267.	আপনার ঘরে/খানায় আপনাদের নিজেদের কতগুলো জীবজন্তু আছে?<br>*[যদি জানা না থাকে তাহলে '99', যদি কিছুই না থাকে তাহলে "00" বসান। যদি সংখ্যা 90 বা তার বেশী হলেও "90" লিখুন]*<br>*[কোন পশু/পাখির খামার বা বিক্রি/ব্যবসার জন্য থাকলে সেই পশু/পাখির সংখ্যা এখানে লিখবেন না]*<br>How many of the following animals are owned by your household?	গরু (Cow)........ |____|____| মহিষ (Buffalo)........ |____|____| ছাগল (Goats)........ |____|____| ভেড়া (Sheeps)........ |____|____| মুরগী (Chickens)........ |____|____| হাঁস (Ducks)........ |____|____| কবুতর (Pigeon)........ |____|____|	
w268.	আপনার স্বামী সাধারনত কোথায় থাকেন?<br>Where does your husband live usually?	আমার সাথে With me........ 1<br>অন্যত্র (দেশের ভিতর) Other place (Within the country)........ 2<br>অন্যত্র (দেশের বাইরে) Other place (Abroad)........ 3<br>অন্যান্য (Other) ______________........ 7	
w269.	আপনাদের কোন নিকট/কাছের আত্মীয় কি বিদেশে থাকেন/কাজ করেন?<br>Do you have any relative living or working outside of Bangladesh?	হ্যাঁ Yes........ 1<br>না No........ 2<br>প্রযোজ্য নয়........ 9	2→w271

	Questions and Filters	Coding Categories	Skip
w270.	তিনি বা তারা কি আপনাকে বা আপনাদেরকে টাকা পাঠান?<br>Do they send you money from abroad?	হ্যাঁ Yes .... 1<br>না No .... 2<br>প্রযোজ্য নয় 9	
w271.	আপনার খানার কোন সদস্যের কি ব্যাংক একাউনট আছে?<br>Does any member of the household have a bank account?	হ্যাঁ Yes .... 1<br>না No .... 2<br>প্রযোজ্য নয় 9	
w272.	আপনার ঘরের/খানার কেউ কি এই সংস্থা/প্রতিষ্ঠানের সদস্য?<br>Does anybody from this household belong to any of the following organizations?	YES NO<br>পিপলস ইন্সটিটিউশন (People's Institution) .... 1 .... 2<br>গ্রামীন ব্যাংক (Grameen Bank) .... 1 .... 2<br>ব্রাক (BRAC) .... 1 .... 2<br>এসএসএস (SSS) .... 1 .... 2<br>আরডিআরএস (RDRS) .... 1 .... 2<br>ব্যুরো বাংলাদেশ (Bureau Bangladesh) .... 1 .... 2<br>বি আর ডি বি (BRDB) .... 1 .... 2<br>প্রশিকা (Proshikha) .... 1 .... 2<br>আশা (ASHA) .... 1 .... 2<br>টিএমএসএস (TMSS) .... 1 .... 2<br>অন্য কোন সংস্থা (Any other organization) .... 1 .... 2<br>নির্দিষ্ট করুন (Specify) _____	
w273.	আপনার পরিবার (খানা) কি বসতবাড়ীর জমির মালিক? হ্যাঁ হলে কতটুকু?<br>*[না হলে 00 লিখুন, জানিনা হলে 99 লিখুন]*<br>Does your family own household land? If yes how much?<br>[If no write 00, if don't know write 99]	|____|____| একর |____|____| শতক<br>Acre Decimal<br>*জানিনা হলে* .... |____|____|	
w274.	আপনার পরিবারের (খানার) কোন চাষযোগ্য জমি আছে কি? হ্যাঁ হলে কতটুকু?<br>*[না হলে 00 লিখুন, জানিনা হলে 99 লিখুন]*<br>Does your family posses any cultivable land? If yes how much?<br>(If no then write 00, if doesn't know then write 99)	|____|____| একর |____|____| শতক<br>Acre Decimal<br>*জানিনা হলে* .... |____|____|	00/99→ w277
w275.	এই সম্পদের বেশীর ভাগ মালিক কে?<br>Who is the owner of most of these wealth?	আমি নিজে Me .... 1<br>আমার স্বামী My husband .... 2<br>উভয়ে Both of us .... 3<br>অন্য কেউ (উল্লেখ করুন)<br>Others (Please mention) ____________ ..... 7<br>জানা নাই/মনে নাই Don't know/can't remember 9	
w276.	বিক্রি করার ক্ষেত্রে বেশীর ভাগ সময় প্রধানত কে সিদ্ধান্ত নিতে পারে?<br>Mainly who can take the decision to sell them?	আমি নিজে Me .... 1<br>আমার স্বামী My husband .... 2<br>উভয়ে Both of us .... 3<br>অন্য কেউ (উল্লেখ করুন)<br>Others (Please mention) ____________ ..... 7<br>জানা নাই/মনে নাই Don't know/can't remember 9	
w277.	একটি নতুন সম্পদ কেনার বেশীরভাগ সময় সিদ্ধান্ত কে নেয়?<br>Most of the time who takes the decision to buy any kind of wealth?	আমি নিজে Me .... 1<br>আমার স্বামী My husband .... 2<br>উভয়ে Both of us .... 3<br>শ্বশুর Father in law .... 4<br>শ্বাশুড়ী Mother in law .... 5<br>অন্য কেউ (উল্লেখ করুন)<br>Others (Please mention) ____________ ..... 7<br>জানা নাই/মনে নাই Don't know/can't remember 9	
w278.	আপনি কোন ধর্মের অনুসারী?<br>What is your religion?	মুসলিম (Muslim) .... 1<br>হিন্দু (Hinduism) .... 2<br>বৌদ্ধ (Buddhism) .... 3<br>খ্রীস্টান (Christianity) .... 4<br>অন্যান্য (Others) ____________ 9	
w279.	আপনি কি কোন আদিবাসী গোত্র/নৃ-গোষ্ঠী/উপজাতি /সম্প্রদায়ের অন্তর্ভূক্ত?<br>Are you belonging to any ethnicity?	হ্যাঁ Yes .... 1<br>না No .... 2<br>প্রযোজ্য নয় 9	**2/9→N1**
w280.	আপনি কোন নৃ-গোষ্ঠী/সম্প্রদায়ের (উপজাতি) অন্তর্ভূক্ত?	হাজং (Hajong) 1	

	Questions and Filters	Coding Categories	Skip
	What is your ethnic group?	গারো (Garo) 2	
		খাসিয়া (Khasia) 3	
		মগ (Mog) 4	
		মুরং (Murong) 5	
		হদি (Hudi) 6	
		অন্যান্য (Others) ____________ 7	

No.	Questions And Filters	Coding Categories	Skip
**তথ্যসংগ্রহকরীর জন্য নির্দেশিকা** Instruction for interviewer**: CHECK Questions**			
N1	**১ লা জানুয়ারী, ২০১৭ থেকে ৩১ অক্টোবার ২০১৭ এর মধ্যের** গর্ভ ফলাফলের জন্য আপনাকে কি এই *মহিলার স্বামীর* নিকট হতে Husband's module (Module H) এর জন্য তথ্য সংগ্রহ করতে বলা হয়েছে?<br>Were you asked to collect information from women's husband for women's pregnancy outcome from 01 January 2017 to 31 October 2017 (Module H)?	হ্যাঁ Yes .... 1<br>না No 2	1 → Module H
N2	উত্তর দাতাকে ধন্যবাদ জানিয়ে সাক্ষাৎকার শেষ করুন।<br>Thanks the mother for providing time and complete the interview.		

Code list for Person
(ব্যক্তির কোড)

দক্ষ/প্রশিক্ষণ প্রাপ্ত স্বাস্থ্য কর্মী Medically trained

a. পাশ করা ডাক্তার MBBS doctor 01
b. নার্স/ধাত্রী Nurse/Midwife 02
c. প্যারামেডিক/মেডিকেল এসিসটেন্ট/সাকমো Paramedic/MA/SACMO 03
d. পরিবার কল্যাণ পরিদর্শক FWV 04
e. সি,এস,বি,এ CSBA 05
f. সি এইচ সি পিCHCP 06
g. স্বাস্থ্য সহকারী/ পরিবার কল্যান সহকারী HA /FWA . 07

অন্যান্য স্বাস্থ্য কর্মী Other health worker

h. পুষ্টি কর্মী CNP 08
i. এনজিও কর্মী NGO worker 09
j. কমিউনিটি স্বেচ্ছাসেবী Community volunteer (CG, CSG 10

অন্যান্য Other

k. প্রশিক্ষণ প্রাপ্ত টিবিএ (প্রশিক্ষণ প্রাপ্ত ধন্নী, চাউনী, দাই) Trained TBA 11
l. প্রশিক্ষণহীন টিবিএ (ধন্নী, চাউনী, দাই) TBA(Dai/Dhorni/Chauni) 12
m. হোমিওপ্যাথ/হোমিওপ্যাথ ওষুধের দোকান Homeopath/Homeopath drug store 13
n. আয়ুর্বেদিক চিকিৎসক / আয়ুর্বেদিক ওষুধের দোকান /হেকিম/ কবিরাজ Ayurved/ Ayurvedic drug store / Hekim/Kabiraj 14
0. গ্রাম ডাক্তার Village doctor 15
p. এলোপ্যাথী ওষুধের দোকান Allopath drug store . 16
q. ইমাম/ঝাড় ফুক/ওঝা Spiritual healer 17
r. পরিবারের অন্যান্য সদস্য/আত্নীয়/ প্রতিবেশী/বন্ধু Family/relative/Neighbor/friend 18
s. স্কুল শিক্ষকSchool teachers 19
t. কমিউনিটি গ্রুপ/ কমিউনিটি সাপোর্ট গ্রুপ CG/CSG Members 20
u. নিজে নিজে 21
x. অন্যান্য Others 97
z. জানি না/ মনে নাই Don’t know/Can’t remember99

Code list for Place
(স্থানের কোড)

বাড়ীতে ঃ

নিজ বাড়ী/ স্বামীর বাড়ী/ বাবার বাড়ী/ রাস্তায়/ নৌকায়..... 11

সরকারী স্বাস্থ্য কেন্দ্র Public Sector

মেডিকেল কলেজ হাসপাতাল Medical College Hospital.......................... 21
জেলা/সদর হাসপাতাল District /Sadar Hospital .. 22
মা ও শিশু স্বাস্থ্য কেন্দ্র MCWC 23
উপজেলা স্বাস্থ্য কমপ্লেক্স UHC............................. 24
ইউনিয়ন স্বাস্থ্য ও পরিবার কল্যাণ কেন্দ্র UH&FWC ... 25
কমিউনিটি ক্লিনিক Community clinic 26
সেটেলাইট ক্লিনিক/ ইপিআই কেন্দ্র Satellite clinic/EPI centre........................... 27
অন্যান্য সরকারী স্বাস্থ্য কেন্দ্র Other Public health facility 28

এনজিও স্বাস্থ্য কেন্দ্র NGO Sector

এনজিও হাসপাতাল/ NGO hospital 31
এনজিও স্থায়ী স্বাস্থ্য কেন্দ্র NGO static health centre 32
এনজিও সেটেলাইট ক্লিনিক NGO satellite clinic ... 33
পুষ্টি কেন্দ্র NNP centre 34
অন্যান্য বেসরকারী স্বাস্থ্য কেন্দ্র Other NGO Health facility 35

প্রাইভেট Private Medical sector

হাসপাতাল/ ক্লিনিক Private Hospital/clinic 41
স্বাস্থ্য কেন্দ্র /ডিসপেনসারী Health centre/Dispensary 42
এমবিবিএস ডাক্তারের চেম্বার MBBS doctor’s chamber............................ 43
গ্রাম ডাক্তারের চেম্বার Village doctor’s chamber.. 44
প্যারামেডিক/মেডিকেল এসিসটেন্ট/সাকমোর চেম্বার Paramedic/MA/SACMO chamber 45
এলোপ্যাথী ঔষধের দোকান Allopath drug store .. 46
অন্যান্য প্রাইভেটস্বাস্থ্য কেন্দ্র Other private Health facility...................... 47
অন্যান্য Other.. 97
জানি না/ মনে নাই Don’t know/Can’t remember99

Code list for Education
(শিক্ষার কোড)

a.No Education / Not passed Class 1 / Non-formal education only.................................. 00
b.Class 1 passed 01
c.Class 2 passed.. 02
d.Class 3 passed 03
e.Class 4 passed.. 04
f.Class 5 passed 05
g.Class 6 passed 06
h.Class 7 passed 07
i.Class 8 passed 08
j.Class 9 passed 09
k.SSC / Dakhil passed 10
l.HSC / Alim passed 12
m.BA / BSS/ BCom / BSc / Fazil/LLB (Pass course) 14
n.BA / BSS/ BCom / BSc / LLB (Hon’s) .. 15
o.MA / MSS/ MCom / MSc/ Kamil/ LLM .. 16
p.PhD/ Postgraduate/ FCPS passed 17
q.Others (nursing, Paramedics, Polytechnic/ LMF) 18
r.Don’t know/Can’t remember 99
